# Supplementary material for: N6-methyladenosine of Spi2a attenuates inflammation and sepsis-associated myocardial dysfunction in mice
Source: Nat Commun. 2023 Mar 2;14:1185. doi: 10.1038/s41467-023-36865-7 (PMC9979126; doi:10.1038/s41467-023-36865-7)
Supplement: Supplementary file 1 — Supplementary information [file 41467_2023_36865_MOESM1_ESM.pdf]

N[6]-methyladenosine of *Spi2a* attenuates inflammation and sepsis-associated myocardial dysfunction in mice

Du et al.

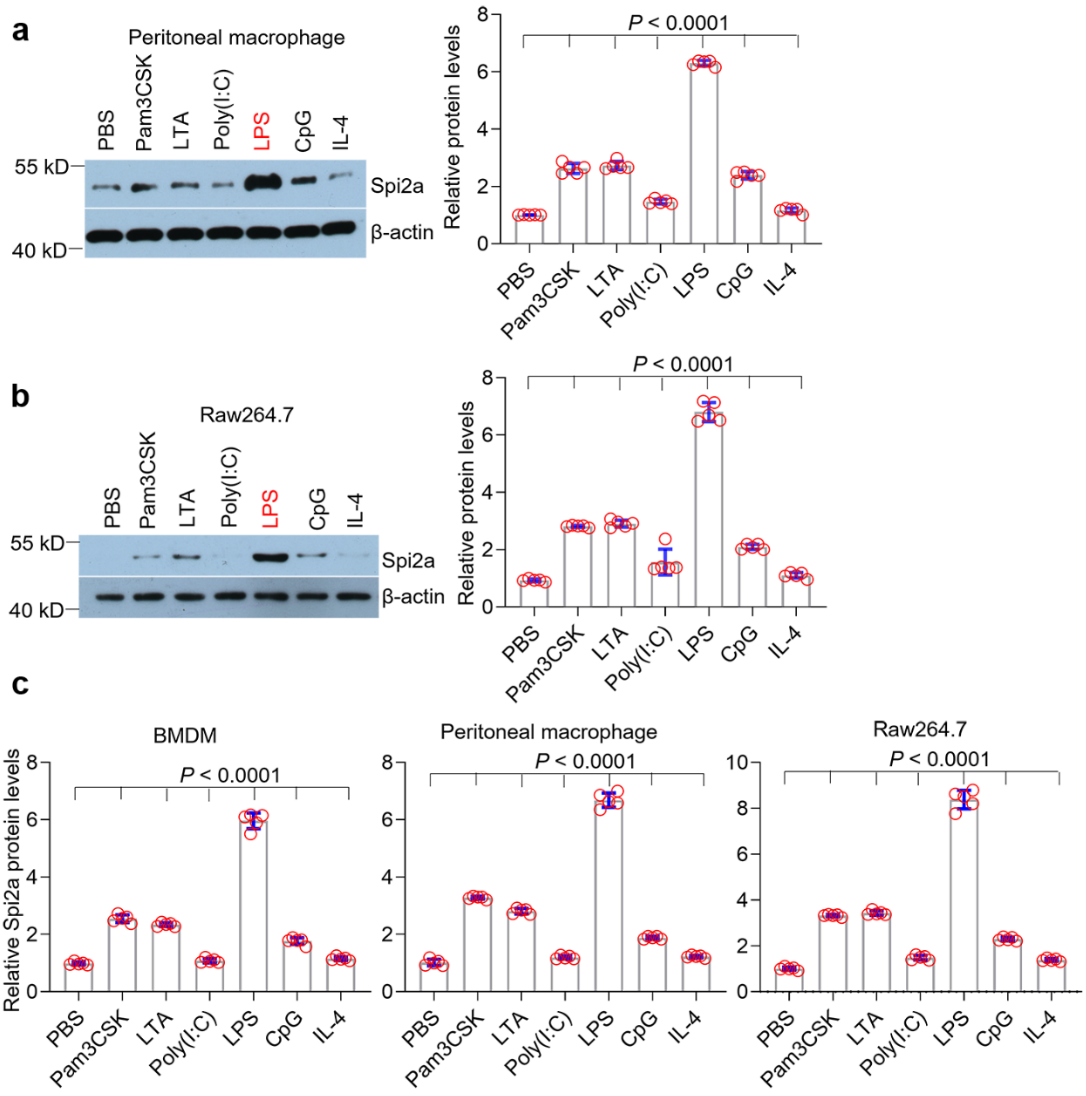

Supplementary figure 1. Spi2a expression in peritoneal macrophages and raw264.7 cells with LPS treatment, Related to Figure 1. (a-b) Western blot and quantitative analyses for Spi2a protein in peritoneal macrophages (a) and raw264.7 cells (b) with PBS, Pam3CSK, LTA, Poly(I:C), LPS, CpG and IL-4 treatment for 8 hours. (c) Quantitative analyses of Spi2a protein in BMDM, peritoneal macrophages and raw264.7 cells with PBS, Pam3CSK, LTA, Poly(I:C), Ultrapure LPS, CpG and IL-4 treatment for 8 hours. n = 5 independent experiments. BMDM, bone marrow-derived macrophage. Data are shown as mean ± SD. One-way two-sided ANOVA was performed for statistical analyses.

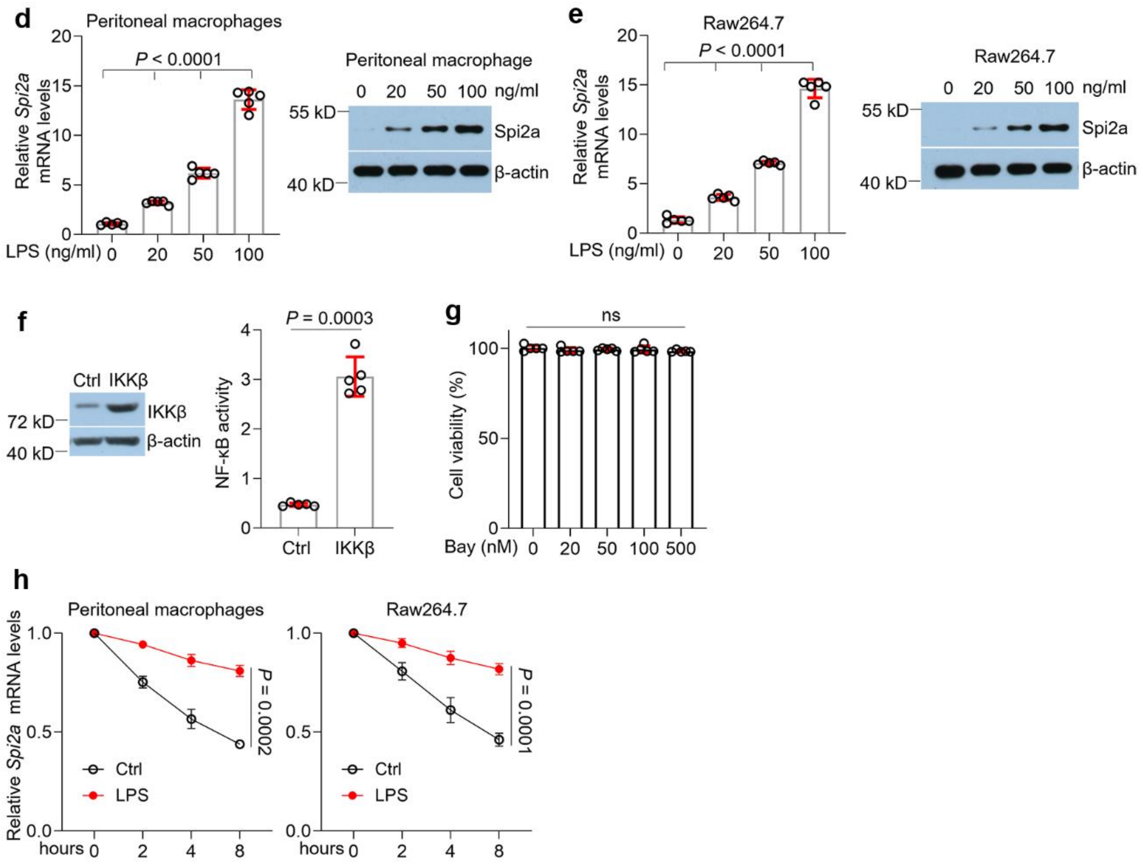

Supplementary figure 1. Spi2a expression in peritoneal macrophages and raw264.7 cells with LPS treatment, Related to Figure 1. (d-e) Real-time PCR and western blot for Spi2a in peritoneal macrophages (d) and raw264.7 cells (e) with LPS (100 ng/ml) at different doses. (f) Western blot analysis of IKKβ levels (left) and NF-κB activity (right) in BMDMs transduced with *Ikkβ*-lentivirus. (g) Cell viability of BMDMs treated with different doses of bay 11-7082 for 20 hours. (h) RNA decay assays for *Spi2a* in peritoneal macrophages (left) and raw264.7 cells (right) with or without LPS (100 ng/ml) treatment at different time points using real-time PCR. n = 5 independent experiments. Cells were all treated with LPS for 8 hours. Ctrl, Control; Bay, Bay 11-7082. Data are shown as mean ± SD. Unpaired two-tailed Student's *t* test (f), one-way (d,e,g) and two-way (h) two-sided ANOVA were performed for statistical analyses.

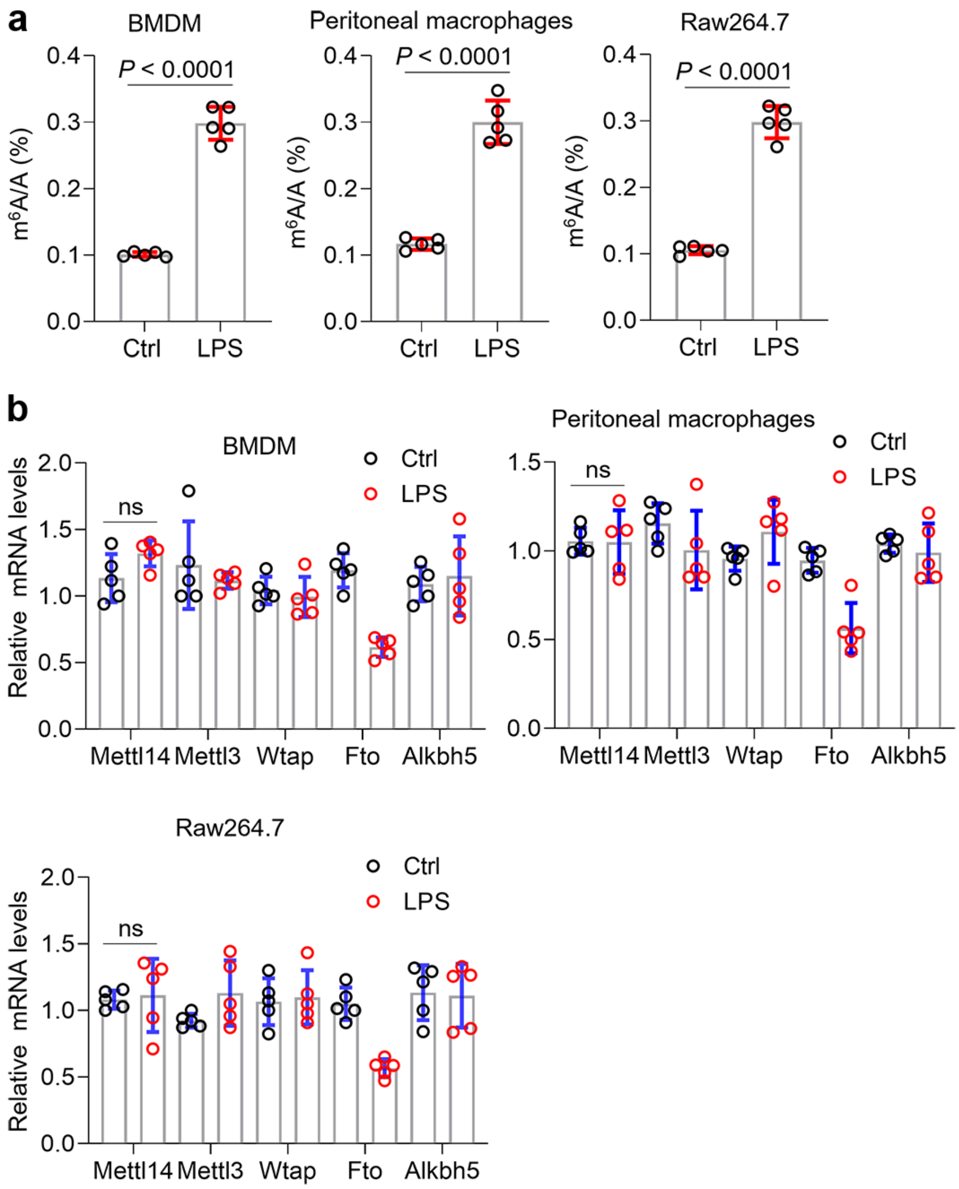

Supplementary figure 2. LPS up-regulates METTL14 protein levels in macrophages, Related to Figure 2. (a) m^{6}A quantification of mRNAs in macrophages treated with or without LPS. (b) Real-time PCR analysis showing the mRNA levels as indicated in macrophages treated with or without LPS. n = 5 independent experiments. Ctrl, Control; Data are shown as mean ± SD. Unpaired two-tailed Student's *t* test was performed for statistical analyses.

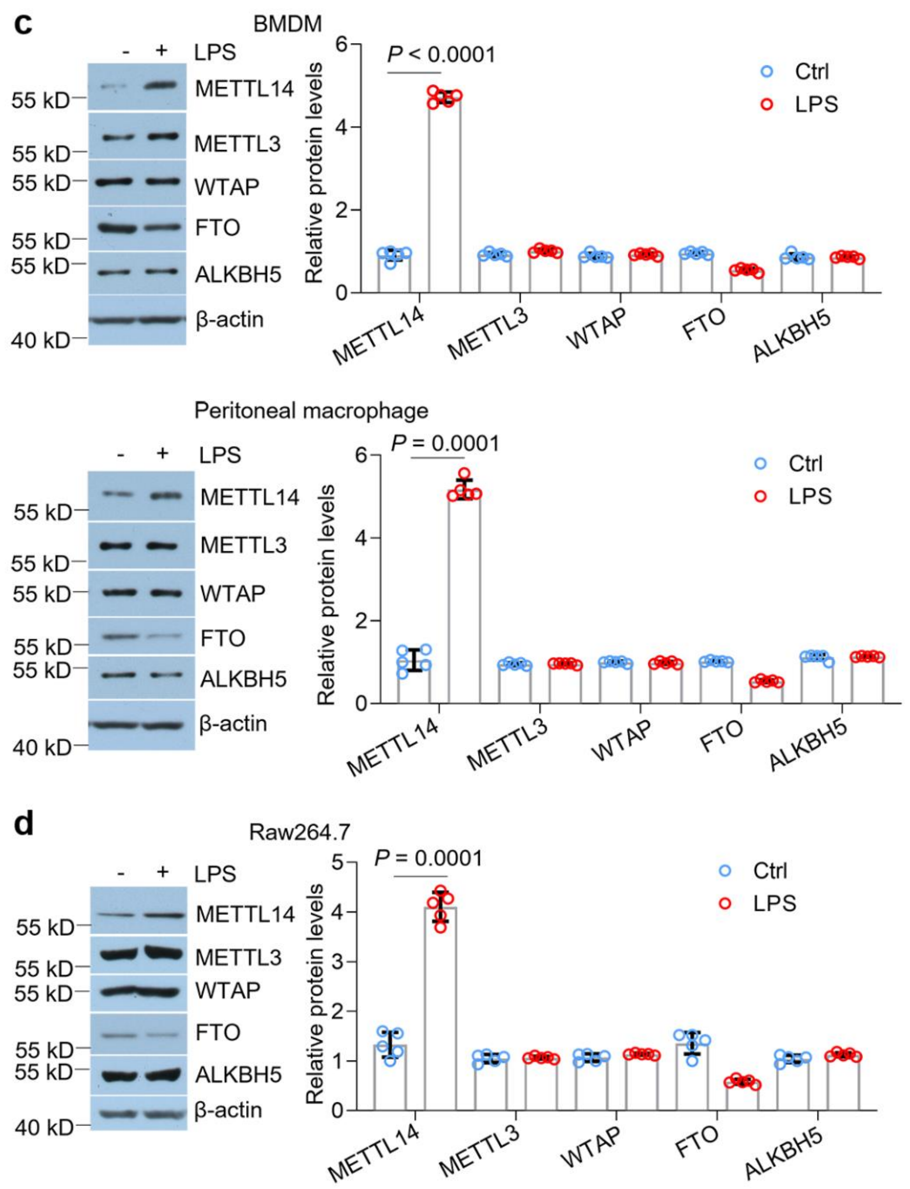

Supplementary figure 2. LPS up-regulates METTL14 protein levels in macrophages, Related to Figure 2. (c-d) Western blot and quantification of indicated proteins in BMDMs or peritoneal macrophages (c) and raw264.7 cells (d) with or without LPS treatment. n = 5 independent experiments. Unless otherwise specified, macrophages were treated with 100 ng/ml LPS for 8 hours. Ctrl, Control. Data are shown as mean ± SD. Unpaired two-tailed Student's *t* test were performed for statistical analyses.

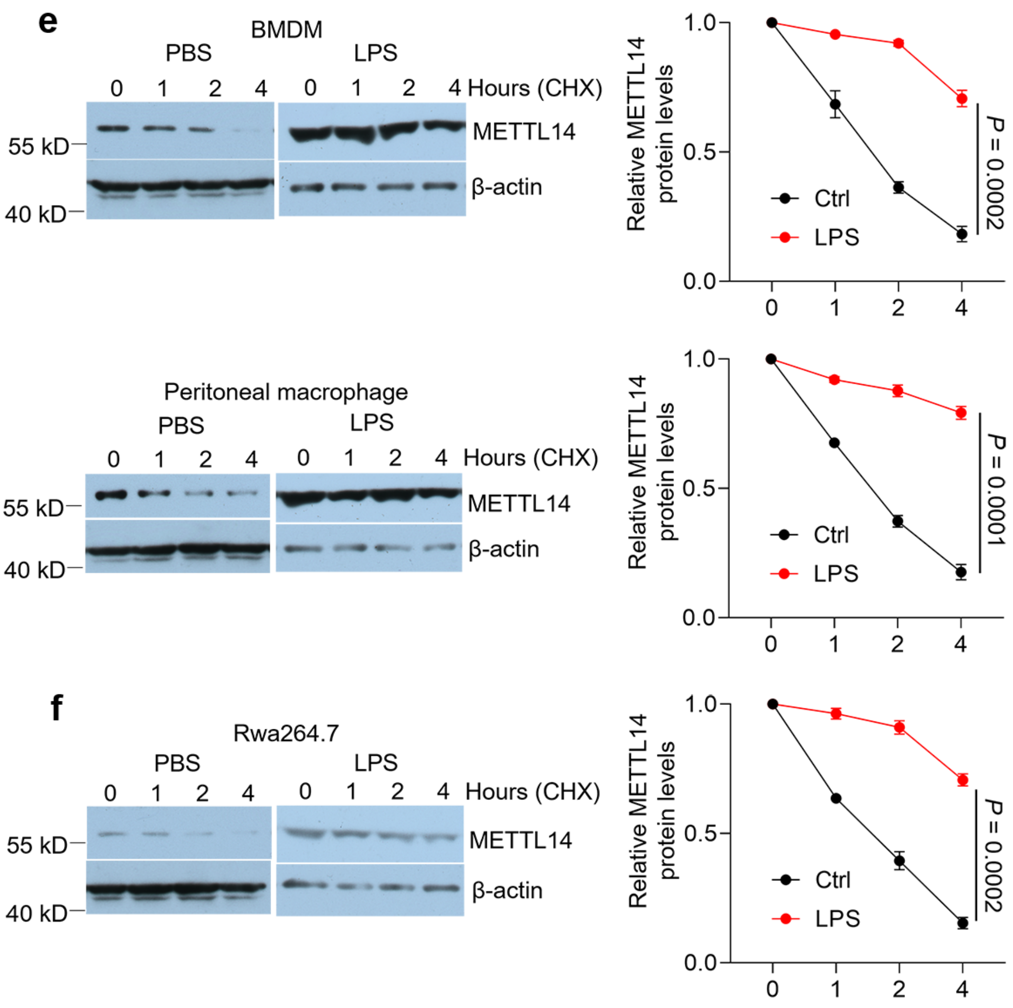

Supplementary figure 2. LPS up-regulates METTL14 protein levels in macrophages, Related to Figure 2. (e-f) Protein decay assays for METTL14 in LPS-treated BMDMs or peritoneal macrophages (e) and raw264.7 cells (f) with different time points after CHX treatment. n = 5 independent experiments. Unless otherwise specified, macrophages were treated with 100 ng/ml LPS for 8 hours. Ctrl, Control. Data are shown as mean ± SD. Two-way two-sided ANOVA was performed for statistical analyses.

g

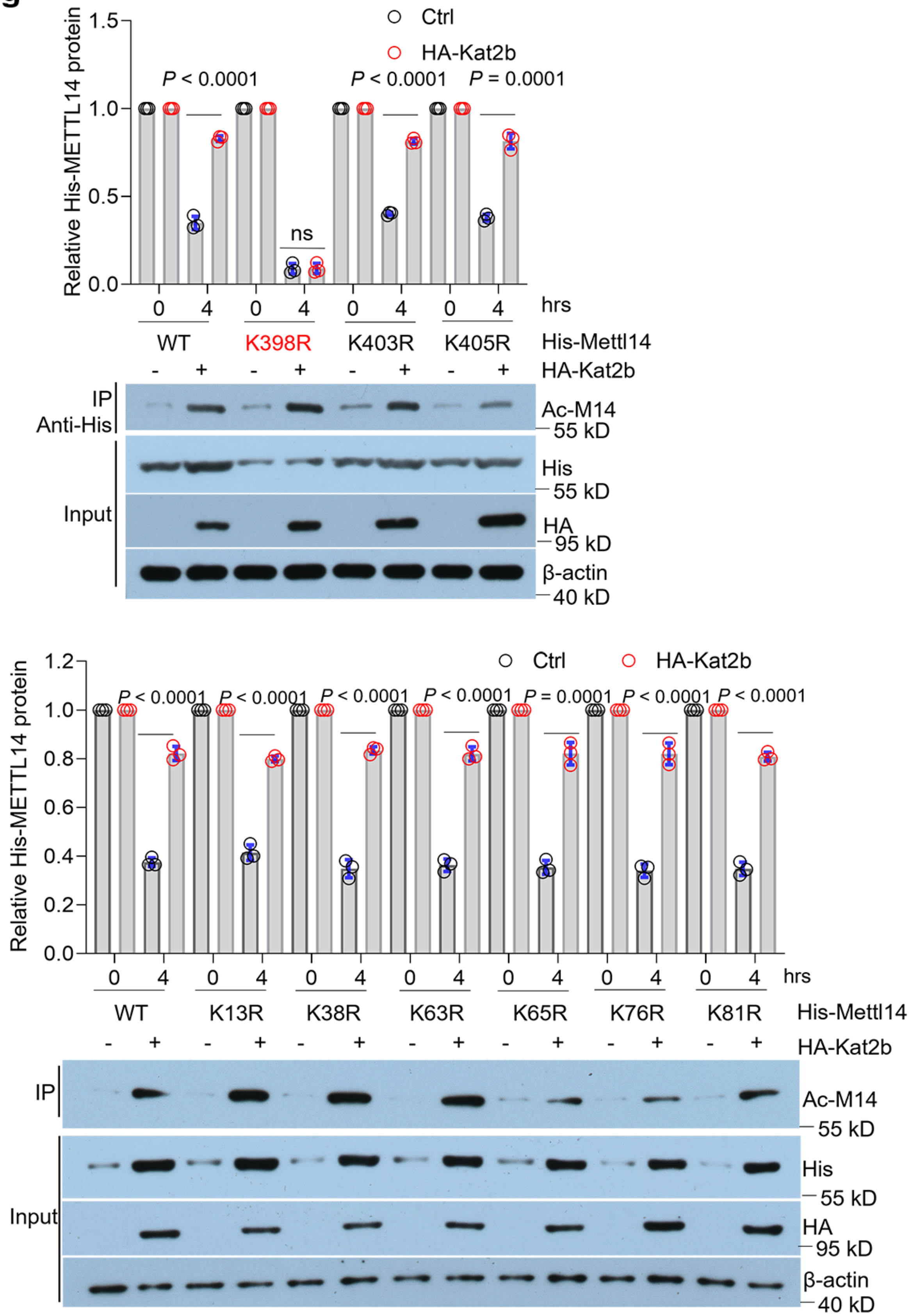

Ctrl
HA-Kat2b
Relative His-METTL14 protein
1.5
1.0
0.5
0.0
P < 0.0001
ns
P < 0.0001
P = 0.0001
0 4 0 4 0 4 0 4 hrs
WT K398R K403R K405R His-Mettl14
- + - + - + - + HA-Kat2b
IP Anti-His
Ac-M14
55 kD
Input
His
55 kD
HA
95 kD
β-actin
40 kD
Ctrl
HA-Kat2b
Relative His-METTL14 protein
1.2
1.0
0.8
0.6
0.4
0.2
0.0
P < 0.0001
P < 0.0001
P < 0.0001
P < 0.0001
P = 0.0001
P < 0.0001
P < 0.0001
0 4 0 4 0 4 0 4 0 4 0 4 0 4 hrs
WT K13R K38R K63R K65R K76R K81R His-Mettl14
- + - + - + - + - + - + - + HA-Kat2b
IP
Ac-M14
55 kD
Input
His
55 kD
HA
95 kD
β-actin
40 kD

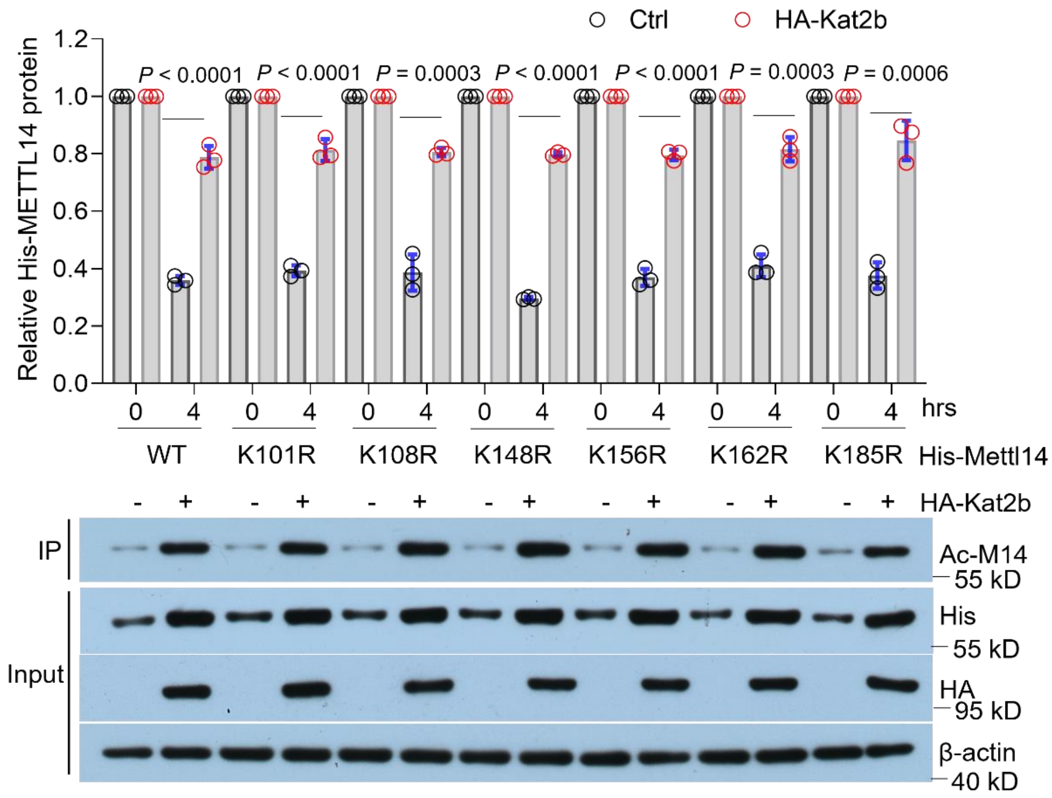
Ctrl
HA-Kat2b
Relative His-METTL14 protein
P < 0.0001
P < 0.0001
P = 0.0003
P < 0.0001
P < 0.0001
P = 0.0003
P = 0.0006
hrs
WT
K101R
K108R
K148R
K156R
K162R
K185R
His-Mettl14
HA-Kat2b
IP
Input
Ac-M14
55 kD
His
55 kD
HA
95 kD
β-actin
40 kD

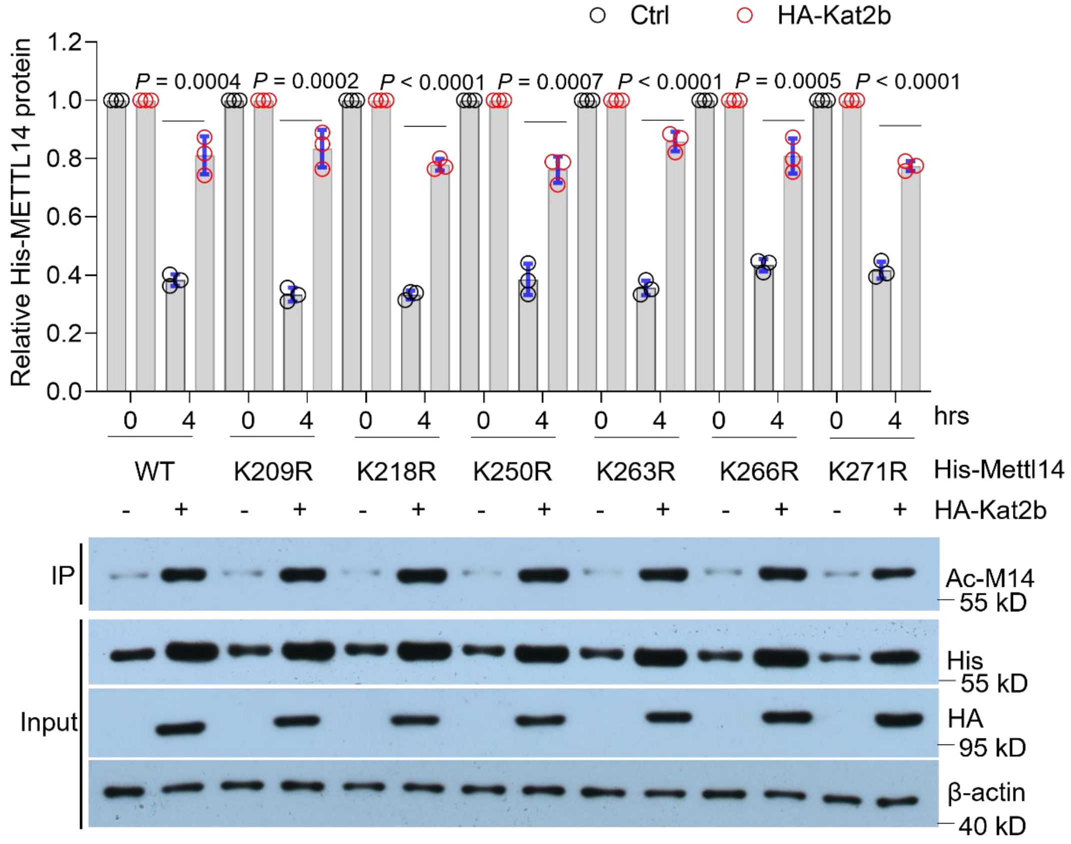
Ctrl
HA-Kat2b
Relative His-METTL14 protein
P = 0.0004
P = 0.0002
P < 0.0001
P = 0.0007
P < 0.0001
P = 0.0005
P < 0.0001
hrs
WT
K209R
K218R
K250R
K263R
K266R
K271R
His-Mettl14
HA-Kat2b
IP
Input
Ac-M14
55 kD
His
55 kD
HA
95 kD
β-actin
40 kD

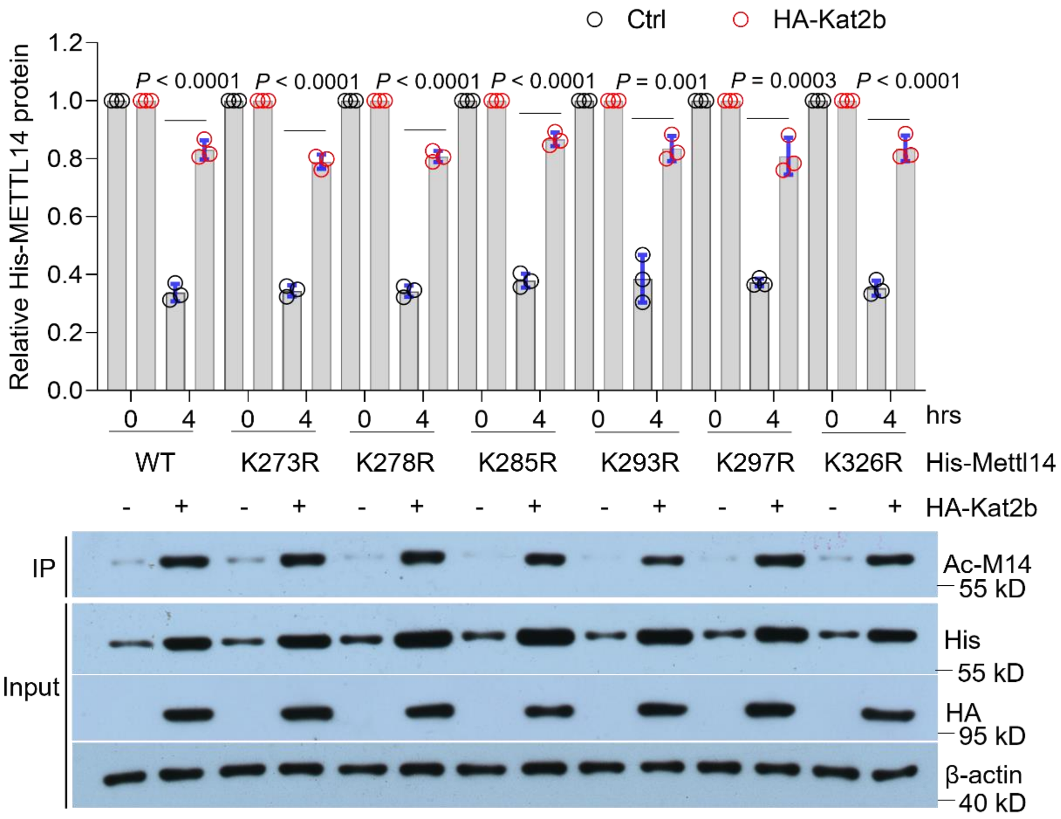

Supplementary figure 2. LPS up-regulates METTL14 protein levels in macrophages, Related to Figure 2. (g) BMDMs were co-infected by His-*Mettl14*-lentivirus variants with representative individual lysine mutation and HA-*Kat2b*-lentivirus as indicated. Protein decay assays for His-METTL14 were performed after 0 or 4 hours CHX treatment (top) and 40 mg cell lysates without CHX treatment were subjected to co-immunoprecipitation (His IP) and western blot analyses (down), n = 3 independent experiments. Unless otherwise specified, macrophages were treated with 100 ng/ml LPS for 8 hours. Ac-M14, Acetylated-METTL14. Data are shown as mean ± SD. Two-way two-sided ANOVA was performed for statistical analyses.

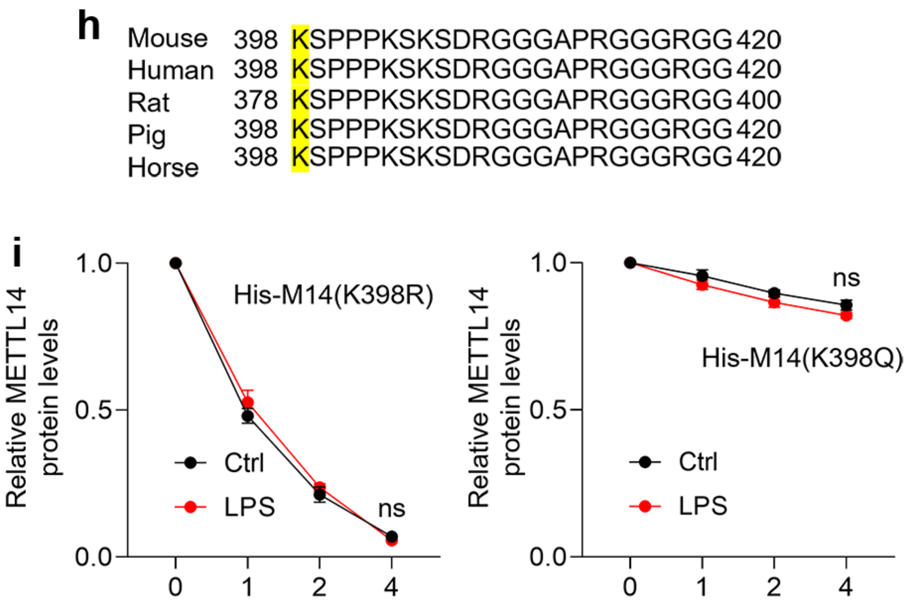

Supplementary figure 2. LPS up-regulates METTL14 protein levels in macrophages, Related to Figure 2. (h) Alignment of METTL14 sequences in different species. Acetylation sites of lysine (K398) were highlighted in yellow. (i) Protein decay assays for His-METTL14 in His-*Mettl14*-lentivirus variants-infected BMDMs with PBS or LPS treatment. n = 5 independent experiments. Unless otherwise specified, macrophages were treated with 100 ng/ml LPS for 8 hours. Ctrl, Control. Data are shown as mean ± SD. Two-way two-sided ANOVA was performed for statistical analyses.

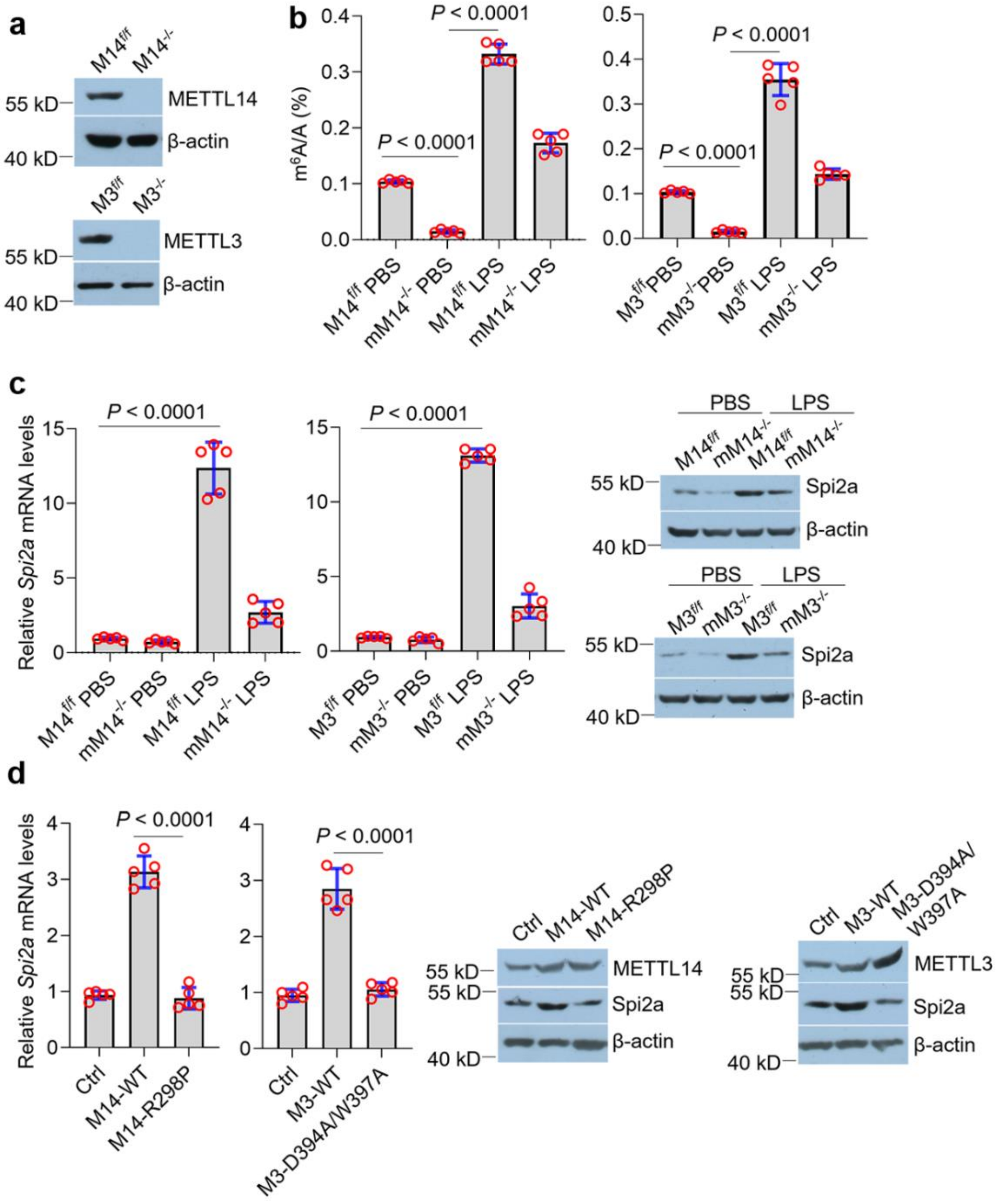

Supplementary figure 3. Effects of m^{6}A methylation on *Spi2a* transcript in BMDMs, Related to Figure 3 and Figure 4. (a) Western blot showing METTL14 protein expression in M14$^{f/f}$ and M14$^{-/-}$ BMDMs (up) and METTL3 levels in M3$^{f/f}$ and M3$^{-/-}$ BMDMs (bottom). (b) m^{6}A quantification of M14$^{f/f}$ and M14$^{-/-}$ BMDMs (left) or M3$^{f/f}$ and M3$^{-/-}$ BMDMs (right) with PBS or LPS treatment. (c) Real-time PCR and western blot showing Spi2a levels in M14$^{f/f}$ and M14$^{-/-}$ BMDMs or M3$^{f/f}$ and M3$^{-/-}$ BMDMs with PBS or LPS treatment. (d) Real-time PCR and western blot showing Spi2a levels in BMDMs infected with wild type *Mettl14*- or *Mettl3*-lentivirus or their variants. n = 5 independent experiments. Cells were all treated with 100 ng/ml LPS for 8 hours. Ctrl, Control; WT, wild type; M14, Mettl14; M3, Mettl3; mM14$^{-/-}$, M14$^{-/-}$ macrophages; mM3$^{-/-}$, M3$^{-/-}$ macrophages. Data are shown as mean ± SD. One-way (d) and two-way (b,c) two-sided ANOVA were performed for statistical analysis.

e

Spi2a-WT (1777-1986)
CTAAGAAAG**GAACC**TGGTGCCCTGGGTCTATCTGCAGCATCTGAAATGCTTGG
TGCCCAGATCTGCCTTATGCTTGCCTTCCTCTGGGCAGAATTATCCTCAGCCC
CTGCATAGTCTCCTGGCCCCACCCAGATCTGGCACAGGTGGAGATCTCAGCC
CTGCAGCTGCATGGGGCCTGTGGGTCAGAGCAGCTCCTCCCCCTGTAGCACT

Spi2a-Mut (1777-1986)
CTAAGAAAG**GATCC**TGGTGCCCTGGGTCTATCTGCAGCATCTGAAATGCTTGG
TGCCCAGATCTGCCTTATGCTTGCCTTCCTCTGGGCAGAATTATCCTCAGCCC
CTGCATAGTCTCCTGGCCCCACCCAGATCTGGCACAGGTGGAGATCTCAGCC
CTGCAGCTGCATGGGGCCTGTGGGTCAGAGCAGCTCCTCCCCCTGTAGCACT

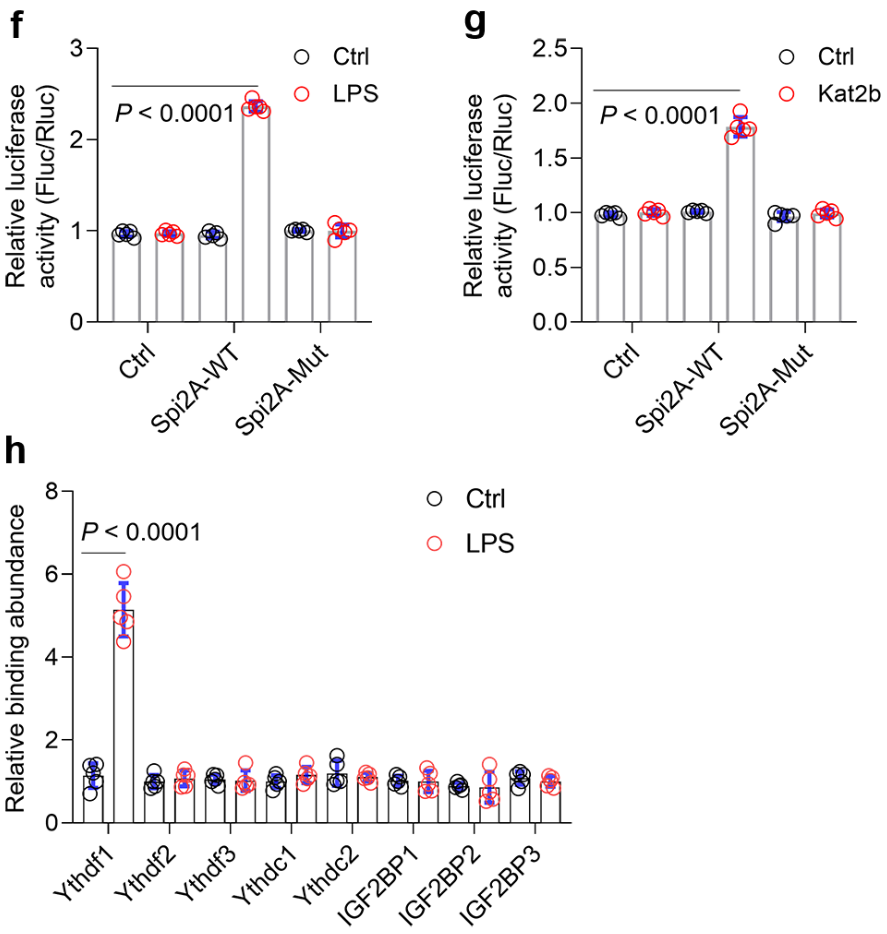

Supplementary figure 3. Effects of m^{6}A methylation on *Spi2a* transcript in BMDMs, Related to Figure 3 and Figure 4. (e) Putative m^{6}A sequences (yellow) in the wildtype (WT) or mutant (mut) cDNA of *Spi2a*. The nucleotide A was mutated into T (red). (f-g) Dual luciferase reporter assays exhibiting the role of LPS (f) or KAT2B (g) in wild-type or mutated *Spi2a* reporters. (h) CLIP assays demonstrating the binding of readers to the *Spi2a* m^{6}A site in BMDMs after LPS treatment. n = 5 independent experiments. Cells were all treated with 100 ng/ml LPS for 8 hours. Ctrl, Control. Data are shown as mean ± SD. Two-way two-sided ANOVA was performed for statistical analysis.

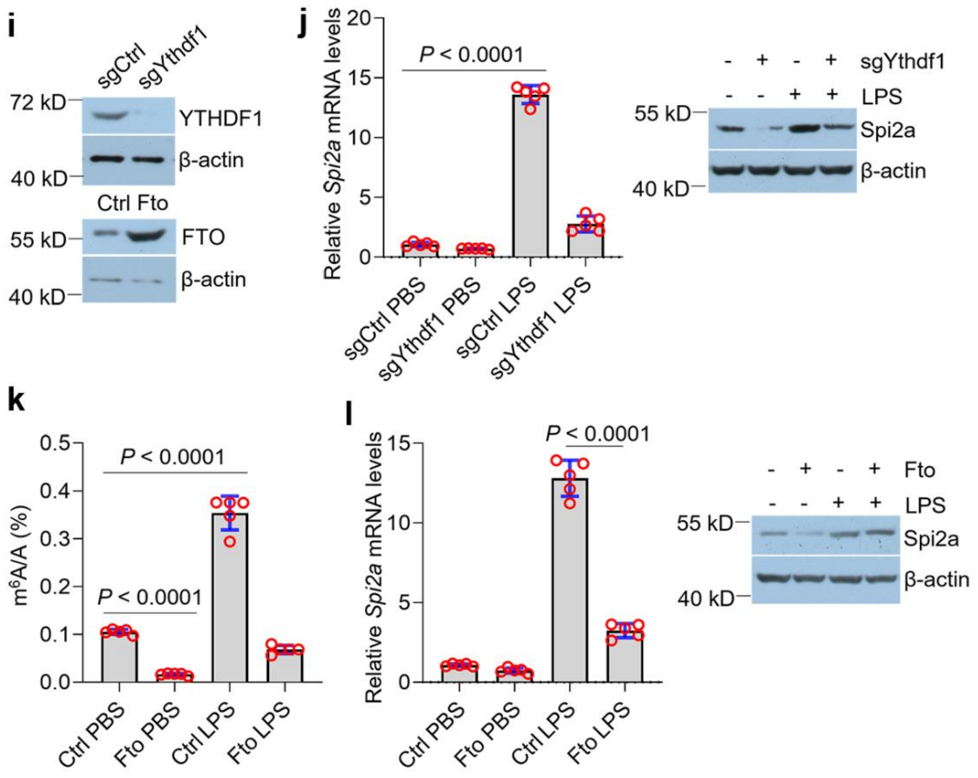

Supplementary figure 3. Effects of m^{6}A methylation on *Spi2a* transcript in BMDMs, Related to Figure 3 and Figure 4. (i) Western blot showing YTHDF1 protein expression in control and *Ythdf1*$^{-/-}$ BMDMs (top) or FTO levels in control and FTO-overexpressing BMDMs (bottom). (j) Spi2a expression in control or *Ythdf1*$^{-/-}$ BMDMs with PBS or LPS treatment tested by real-time PCR (left) or western blot (right). (k) m^{6}A quantification of control and FTO-overexpressing BMDMs with PBS or LPS treatment. (l) Spi2a expression of control and FTO-overexpressing BMDMs with PBS or LPS treatment detected by real-time PCR (left) or western blot (right). n = 5 independent experiments. Cells were all treated with 100 ng/ml LPS for 8 hours. Ctrl, Control. Data are shown as mean ± SD. Two-way two-sided ANOVA was performed for statistical analysis.

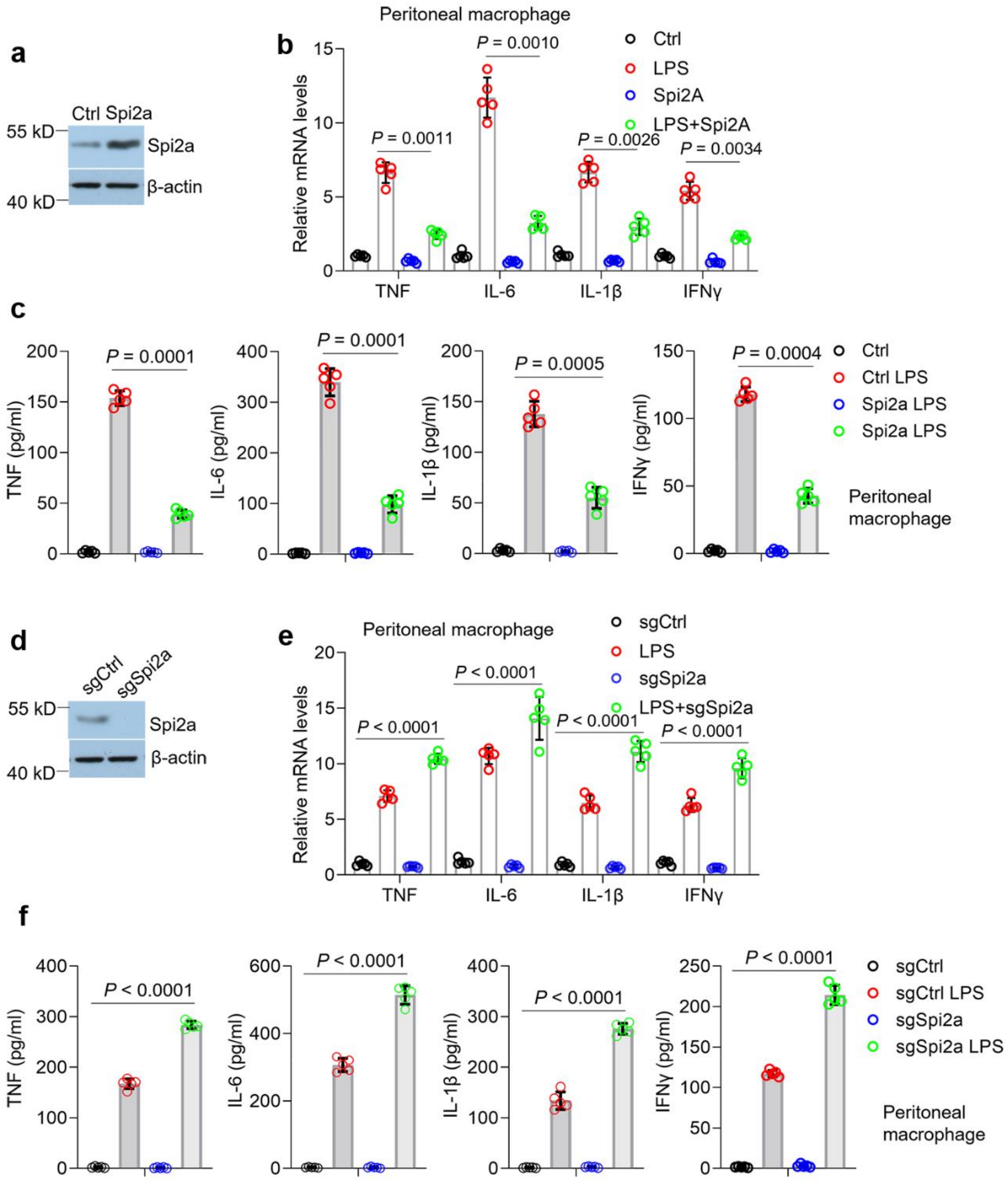

Supplementary figure 4. Spi2a binds to IKKβ in peritoneal macrophages to suppress cytokines, Related to Figure 5 and Figure 6. (a) Western blot for Spi2a expression in peritoneal macrophages infected with or without *Spi2a*-lentivirus. (b) Real-time PCR showing mRNA expression of cytokine in control- or *Spi2a*-lentivirus-infected peritoneal macrophages with or without LPS treatment. (c) Elisa detection of cytokine secretion in the culture medium of control- or *Spi2a*-lentivirus-infected peritoneal macrophages with or without LPS treatment. (d) Western blot for Spi2a expression in peritoneal macrophages infected with or without sg*Spi2a*-lentivirus. (e) Real-time PCR showing mRNA expression of cytokine in sgCtrl- or sg*Spi2a*-lentivirus-infected peritoneal macrophages with or without LPS treatment. (f) Elisa assays demonstrating

secretion of cytokine in the culture medium of sgCtrl- or sg*Spi2a*-lentivirus-infected peritoneal macrophages with or without LPS treatment. n = 5 independent experiments. Cells were all treated with 100 ng/ml LPS for 8 hours. Ctrl, Control. Data are shown as mean ± SD. Two-way two-sided ANOVA was performed for statistical analyses.

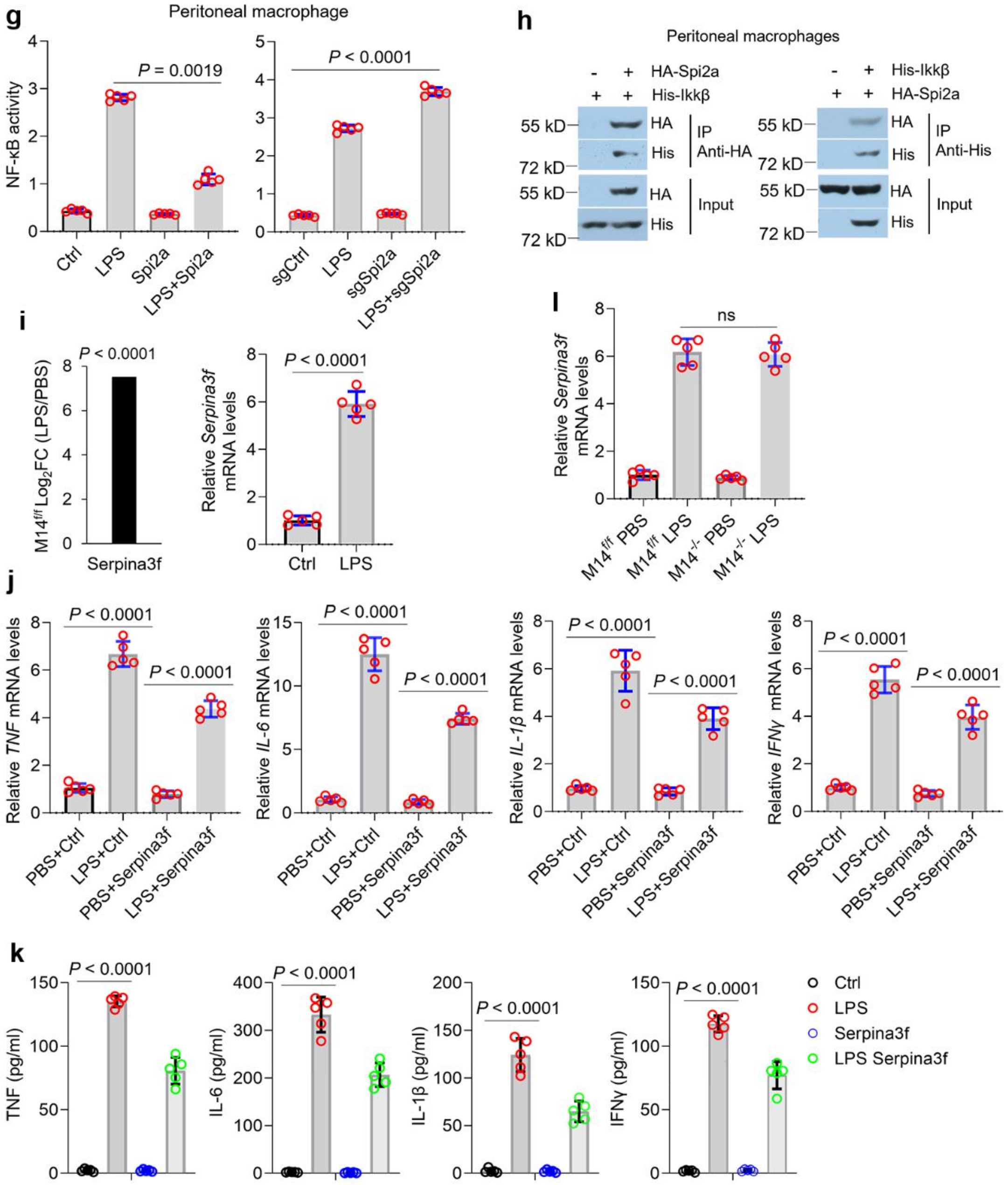

Supplementary figure 4. Spi2a binds to IKKβ in peritoneal macrophages to suppress cytokines, Related to Figure 5 and Figure 6. (g) NF-κB activity assays of control- or *Spi2a*-lentivirus-infected peritoneal macrophages (left) or sgCtrl- or sg*Spi2a*-lentivirus-infected peritoneal macrophages (right) with PBS or LPS challenge. (h) Peritoneal macrophages were infected by HA-*Spi2a*- or His-*Ikkβ*-lentivirus as indicated and immunoprecipitated by HA (left) or His (right) antibody, followed by western blot analyses. (i) *Serpina3f* levels in RNA-sequencing data (left) and real-time PCR for *Serpina3f* levels in BMDMs with or without LPS treatment (right). (j-k) Real-time PCR (j) or Elisa (k) assays for cytokine expression in control- or *Serpina3f*-lentivirus-infected BMDMs with PBS or LPS treatment. (l) Real-time PCR for *Serpina3f* in $M14^{f/f}$ and $M14^{-/-}$ BMDMs with PBS or LPS treatment. n = 5

independent experiments. Cells were all treated with 100 ng/ml LPS for 8 hours. Ctrl, Control; M14, Mettl14. Data are shown as mean ± SD. Unpaired two-tailed Student's *t* test (i) and two-way two-sided ANOVA (g,j,k,l) were performed for statistical analyses.

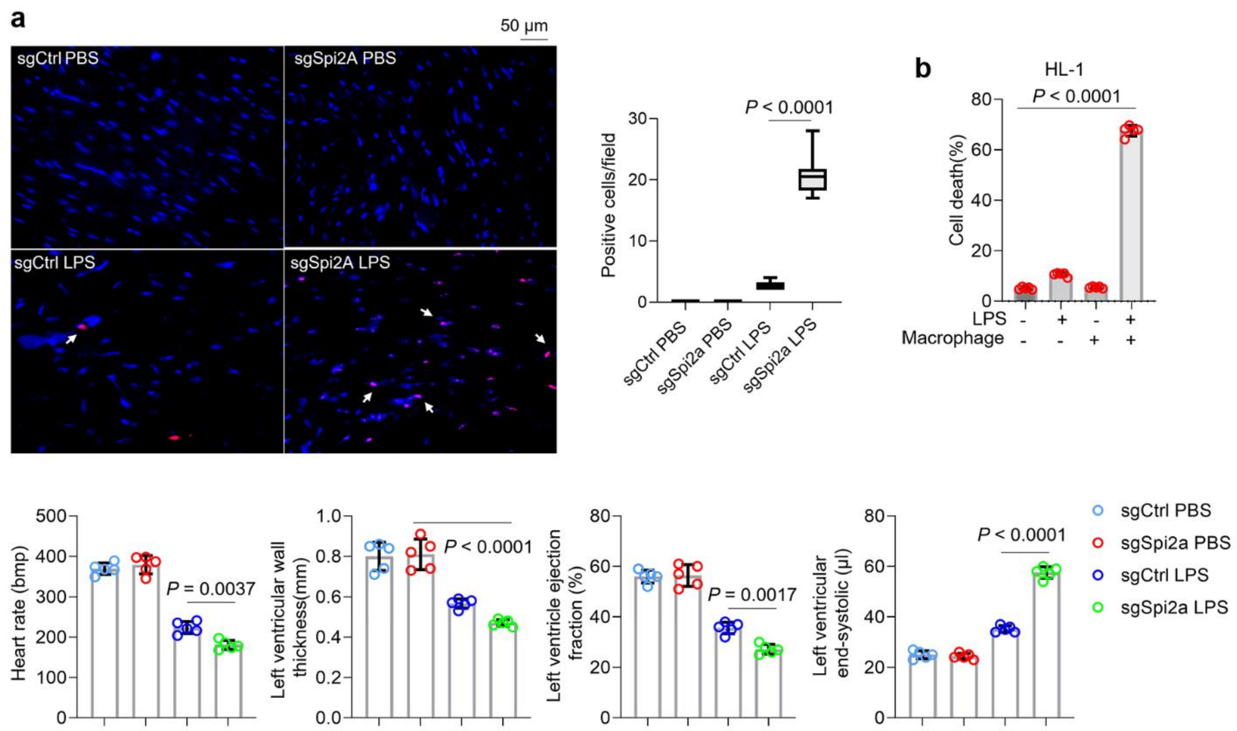

Supplementary figure 5. The roles of Spi2a in animal models, Related to Figure 7 and Figure 8. (a) TUNEL staining for dead cells (indicated by white arrows) detection in heart tissues and quantitative analysis displayed by box-and-whisker plot (top, n = 20 fields in each group) and heart functions of reconstituted mice injected with PBS or LPS (bottom, n = 5 in each group). (b) Cell death assays of HL-1 cells treated with culture medium from PBS- or LPS-challenged BMDMs for 8 hours, n = 5 in each group. For control groups, HL-1 cells were treated with or without LPS directly for 8 hours. For box-and-whisker plot, bottom line means lower quartile, middle line means median, top line means upper quartile, whiskers mean minimum and maximum. Ctrl, Control. Data are shown as mean ± SD. Two-way two-sided ANOVA was performed for statistical analyses.

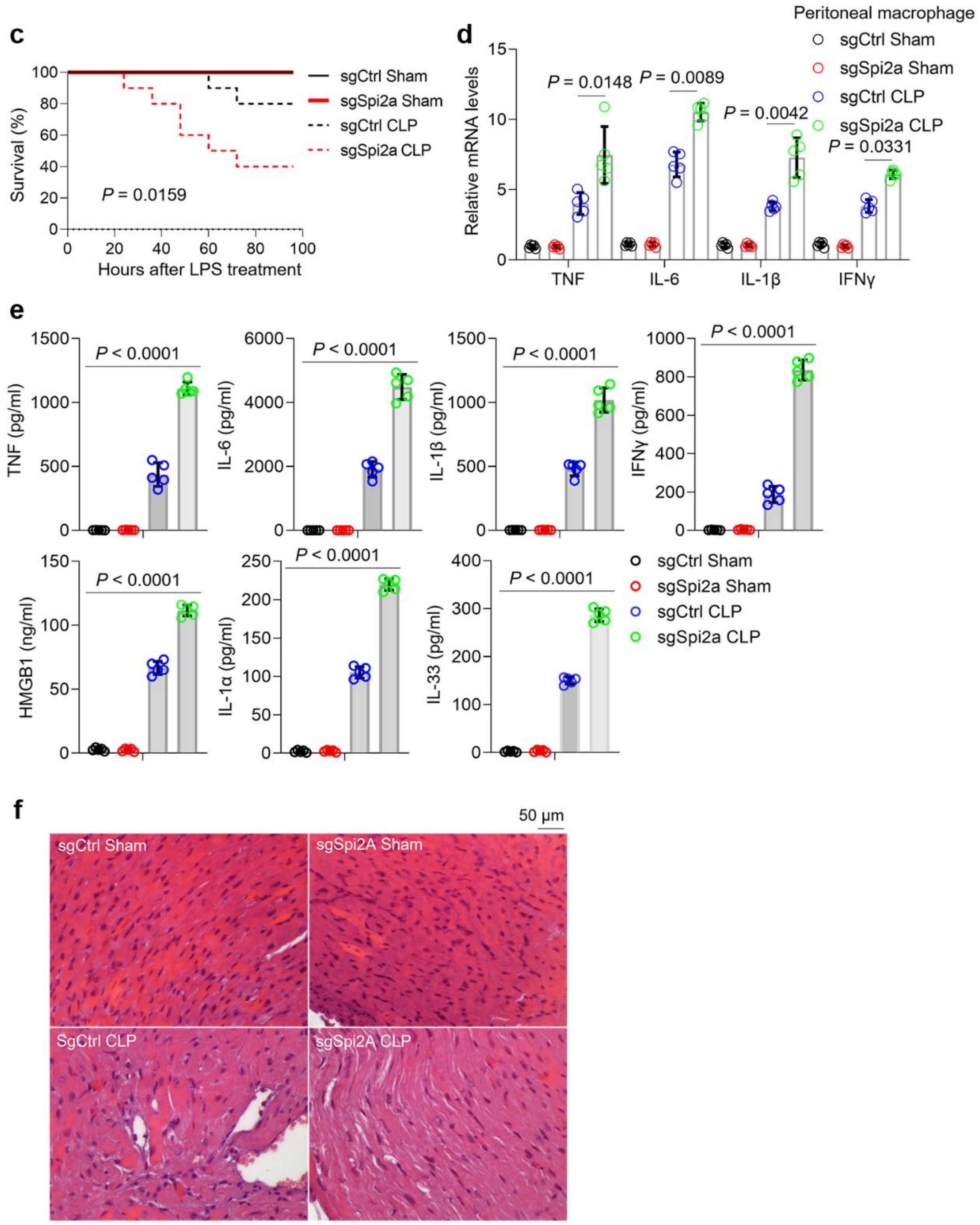

Supplementary figure 5. The roles of Spi2a in animal models, Related to Figure 7 and Figure 8. (c-j) Macrophage-depleted wild type mice were reconstituted with wild type BMDMs transduced with sgCtrl- or sg*Spi2a*-lentivirus prior to sham or CLP surgery, followed by these assays: Kaplan-Meier survival curves (c); n = 10 in each group; Real-time PCR for cytokine expression in peritoneal macrophages (d); n = 5 in each group; Elisa detection for cytokine and DAMPs in sera (e) n = 5 in each group; HE examination of heart tissues (f), images were representative of 5 independent experiments; Ctrl, Control. Data are shown as mean ± SD. Log-rank test (c) and two-way two-sided ANOVA (d,e) were performed for statistical analyses.

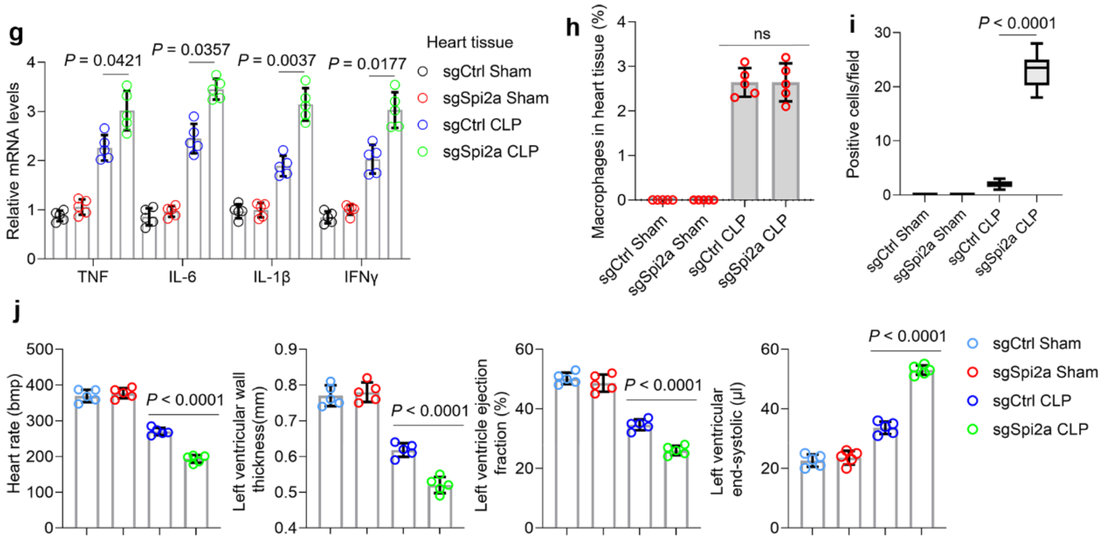

Supplementary figure 5. The roles of Spi2a in animal models, Related to Figure 7 and Figure 8. Real-time PCR for cytokine expression in heart tissues (g) n = 5 in each group; MACS for the percentage of F4/80-positive macrophages in heart tissue (h), n = 5 in each group; Quantitative analysis of TUNEL staining-positive cells in heart tissues displayed by box-and-whisker plot (i), n = 20 fields in each group; Measurements of Heart functions (j), n = 5 in each group. For box-and-whisker plot, bottom line means lower quartile, middle line means median, top line means upper quartile, whiskers mean minimum and maximum. Ctrl, Control. Data are shown as mean ± SD. Two-way two-sided ANOVA was performed for statistical analyses.

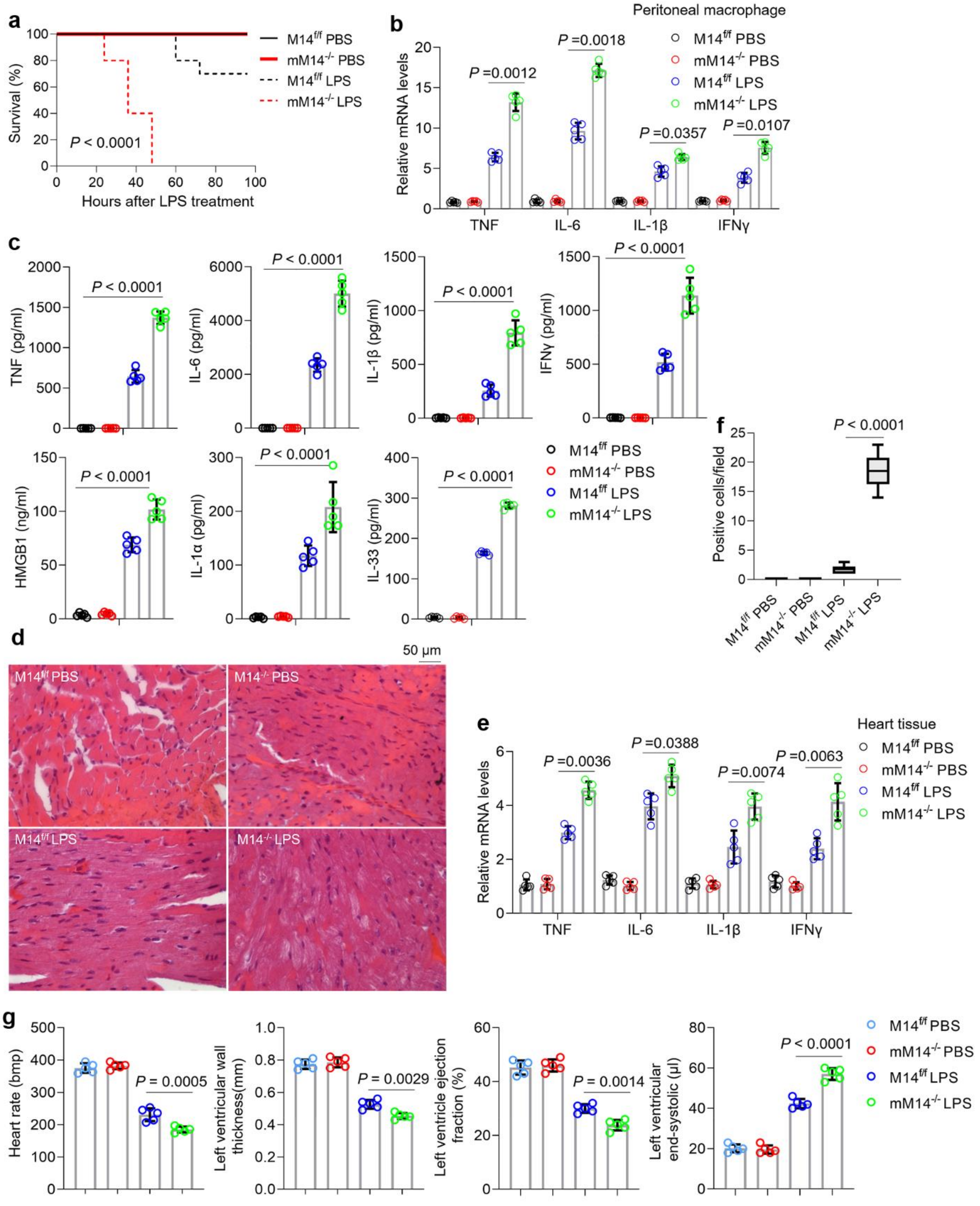

Supplementary figure 6. Roles of macrophages m^{6}A methylation in sepsis. (a-g) M14$^{f/f}$ and mM14$^{-/-}$ mice were injected with PBS or LPS for the following assays: Kaplan-Meier survival curves (a), n = 10 in each group; Real-time PCR for cytokine expression in peritoneal macrophages (b), n = 5 in each group; Elisa detection for cytokine and DAMPs in sera (c) n = 5 in each group; HE examination of heart tissues (d), images were representative of 5 independent experiments; Real-time PCR for

cytokine expression in heart tissues (e), n = 5 in each group; Quantitative analysis of TUNEL staining-positive cells in heart tissues displayed by box-and-whisker plot (f), n = 20 fields in each group; Measurements of Heart functions (g), n = 5 in each group. For box-and-whisker plot, bottom line means lower quartile, middle line means median, top line means upper quartile, whiskers mean minimum and maximum. Ctrl, Control, M14, Mettl14; M3, Mettl3; $mM14^{-/-}$, $M14^{-/-}$ macrophages; $mM3^{-/-}$, $M3^{-/-}$ macrophages. Data are shown as mean ± SD. Log-rank test (a) and two-way two-sided ANOVA (b,c,e,f,g) were performed for statistical analyses.

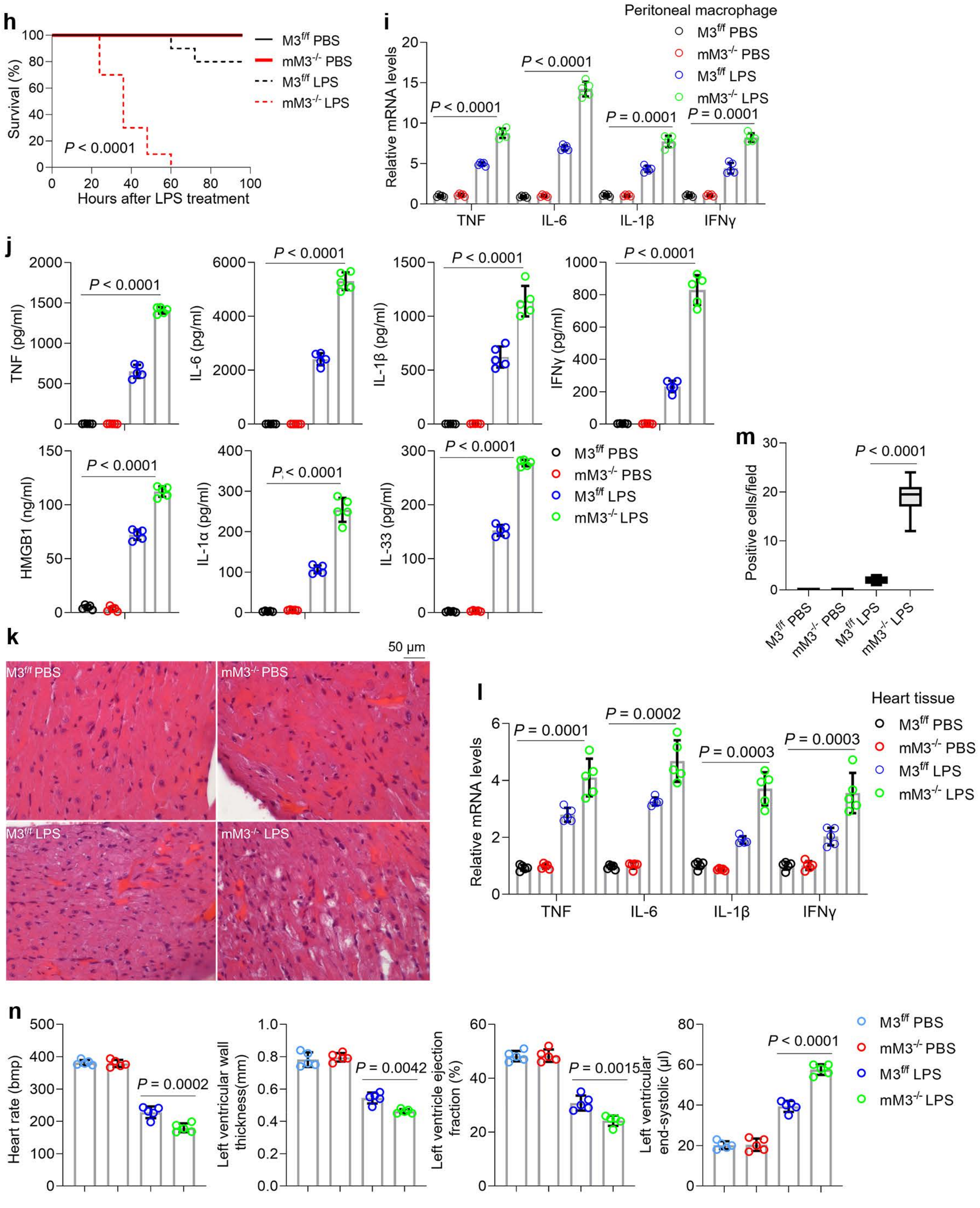

Supplementary figure 6. Roles of macrophages m^{6}A methylation in sepsis. (h-n) M3$^{f/f}$ and mM3$^{-/-}$ mice were injected with PBS or LPS for the following assays: Kaplan-Meier survival curves (h), n = 10 in each group; Real-time PCR for cytokine expression in peritoneal macrophages (i), n = 5 in each group; Elisa detection for cytokine and DAMPs in sera (j) n = 5 in each group; HE examination of heart tissues (k), images were representative of 5 independent experiments; Real-time PCR for cytokine expression in heart tissues (l) n = 5 in each group; Quantitative analysis of TUNEL staining-positive cells in heart tissues displayed by box-and-whisker plot (m),

n = 20 fields in each group; Measurements of Heart functions (n), n = 5 in each group. For box-and-whisker plot, bottom line means lower quartile, middle line means median, top line means upper quartile, whiskers mean minimum and maximum. Ctrl, Control, M14, Mettl14; M3, Mettl3; $mM14^{-/-}$, $M14^{-/-}$ macrophages; $mM3^{-/-}$, $M3^{-/-}$ macrophages. Data are shown as mean ± SD. Log-rank test (h) and two-way two-sided ANOVA (i,j,l,m,n) were performed for statistical analyses.

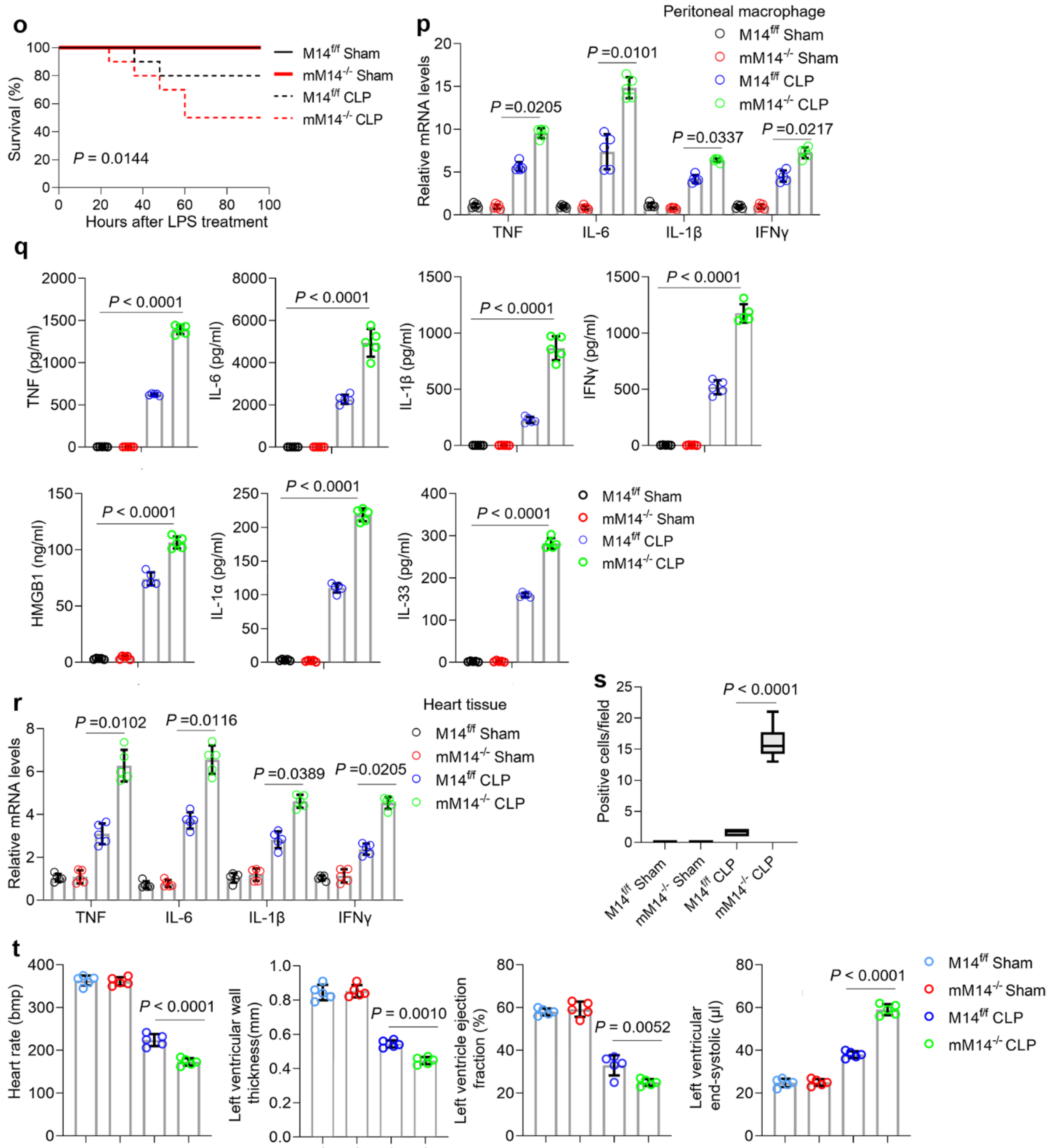

Supplementary figure 6. Roles of macrophages m^{6}A methylation in sepsis. (o-t) M14$^{f/f}$ and mM14$^{-/-}$ mice were subjected to sham or CLP surgery, followed by these assays: Kaplan-Meier survival curves (o), n = 10 in each group; Real-time PCR for cytokine expression in peritoneal macrophages (p), n = 5 in each group; Elisa detection for cytokine and DAMPs in sera (q) n = 5 in each group; Real-time PCR for cytokine expression in heart tissues (r), n = 5 in each group; Quantitative analysis of TUNEL staining-positive cells in heart tissues displayed by box-and-whisker plot (s), n = 20 fields in each group; Measurements of Heart functions (t), n = 5 in each group. For box-and-whisker plot, bottom line means lower quartile, middle line means median, top line means upper quartile, whiskers mean minimum and maximum. Ctrl, Control.

Data are shown as mean ± SD. Log-rank test (o) and two-way two-sided ANOVA (p,q,r,s,t) were performed for statistical analyses.

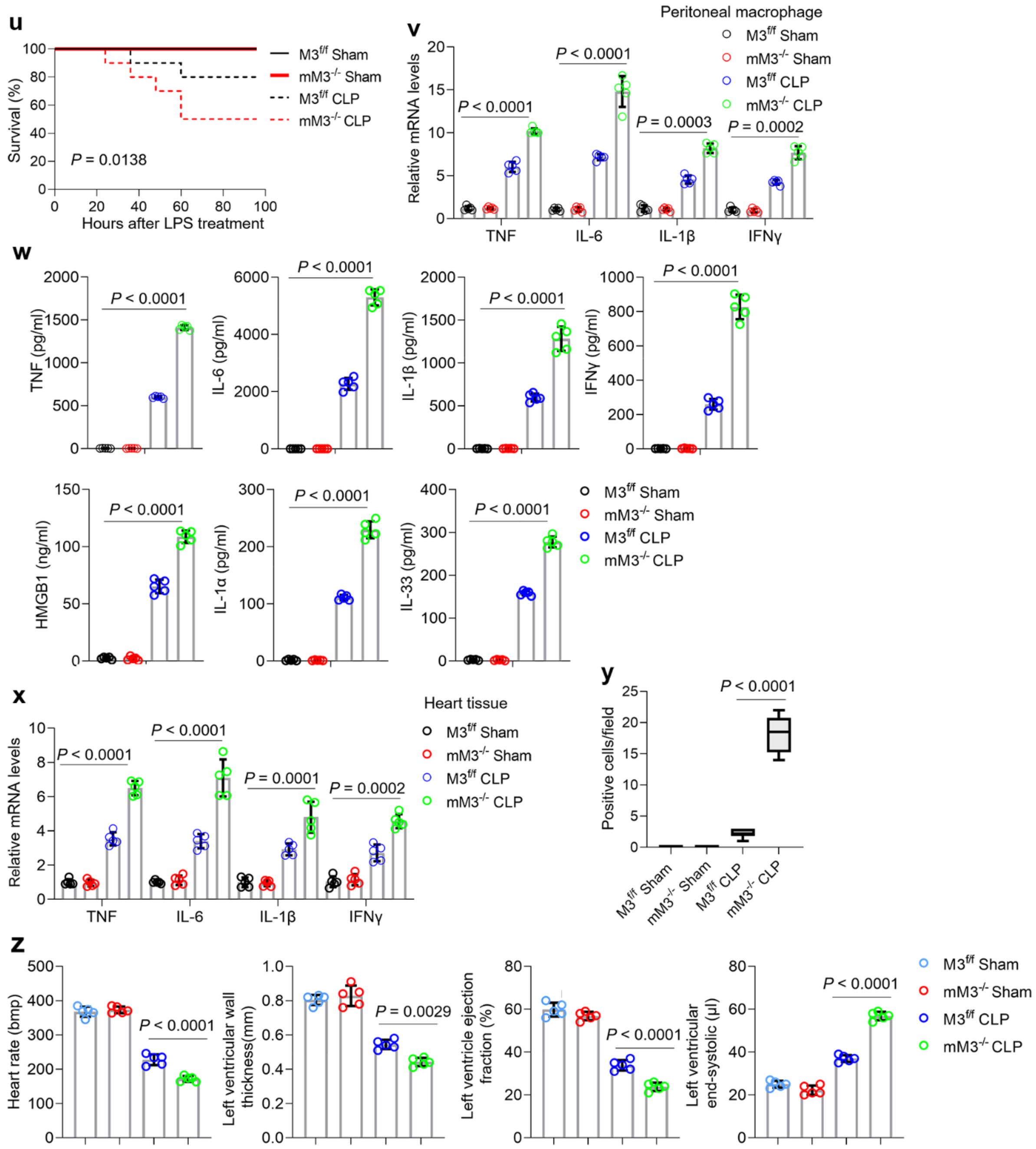

Supplementary figure 6. Roles of macrophages m^{6}A methylation in sepsis. (u-z) M3$^{f/f}$ and mM3$^{-/-}$ mice were subjected to sham or CLP surgery, followed by these assays: Kaplan-Meier survival curves (u), n = 10 in each group; Real-time PCR for cytokine expression in peritoneal macrophages (v), n = 5 in each group; Elisa detection for cytokine and DAMPs in sera (w) n = 5 in each group; Real-time PCR for cytokine expression in heart tissues (x), n = 5 in each group; Quantitative analysis of TUNEL staining-positive cells in heart tissues displayed by box-and-whisker plot (y), n = 20 fields in each group; Measurements of Heart functions (z), n = 5 in each group. For box-and-whisker plot, bottom line means lower quartile, middle line means median, top line means upper quartile, whiskers mean minimum and maximum. Ctrl, Control.

Data are shown as mean ± SD. Log-rank test (u) and two-way two-sided ANOVA (v,w,x,y,z) were performed for statistical analyses.

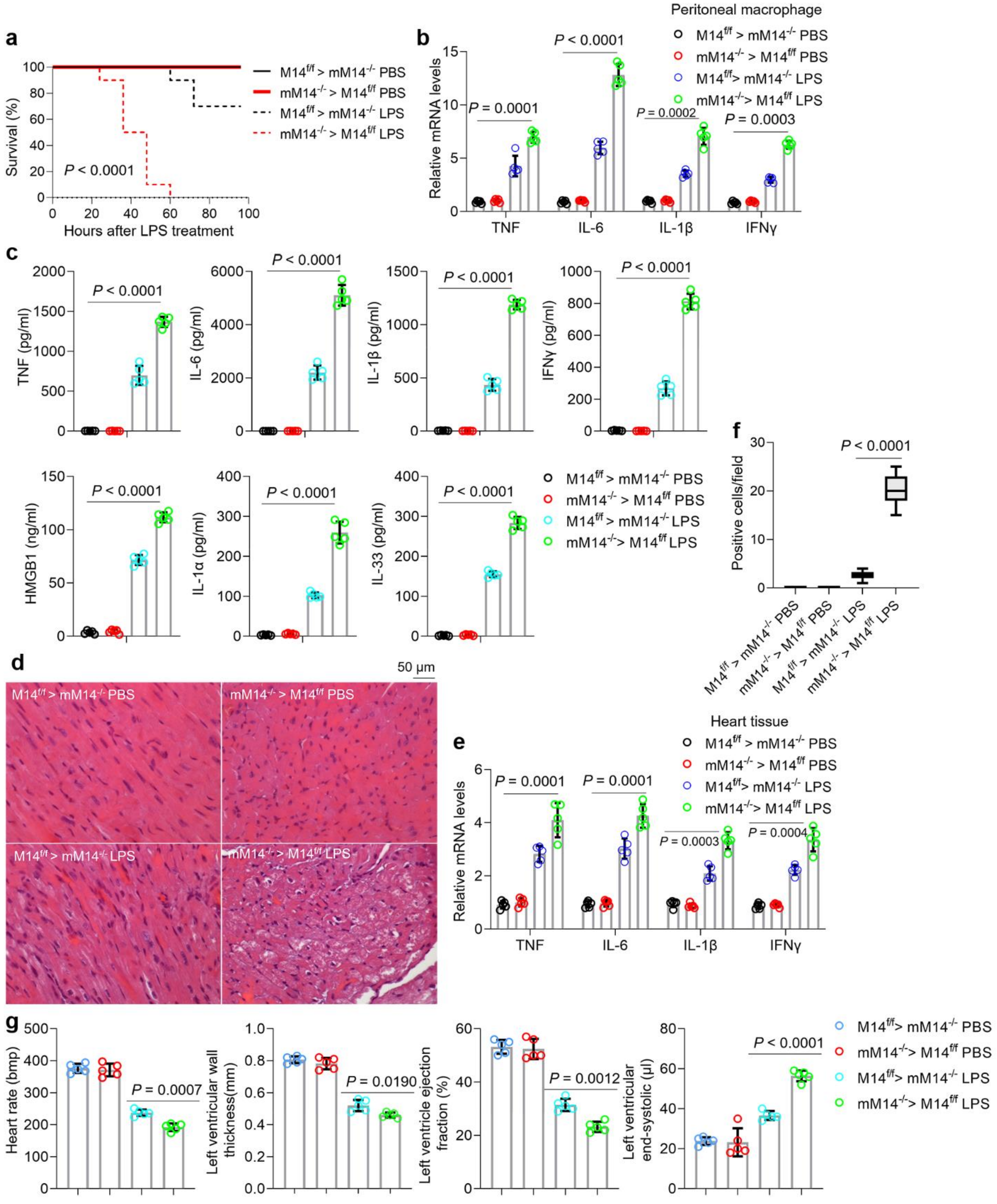

Supplementary figure 7. Roles of m^{6}A methylation in macrophages depletion and reconstitution mice in sepsis. (a-g) M14$^{f/f}$ and mM14$^{-/-}$ recipient mice were treated with clodronate liposomes to deplete macrophages and reconstituted with donor BMDMs crossly (M14$^{f/f}$ donor BMDMs > mM14$^{-/-}$ recipient mice; mM14$^{-/-}$ donor BMDMs > M14$^{f/f}$ recipient mice) prior to PBS or LPS injection for the following experiments: Kaplan-Meier survival curves (a), n = 10 in each group; Real-time PCR

for cytokine expression in peritoneal macrophages (b), n = 5 in each group; Elisa detection for cytokine and DAMPs in sera (c) n = 5 in each group; HE examination of heart tissues (d), images were representative of 5 independent experiments; Real-time PCR for cytokine expression in heart tissues (e), n = 5 in each group; Quantitative analysis of TUNEL staining-positive cells in heart tissues displayed by box-and-whisker plot (f), n = 20 fields in each group; Measurements of Heart functions (g), n = 5 in each group. For box-and-whisker plot, bottom line means lower quartile, middle line means median, top line means upper quartile, whiskers mean minimum and maximum. $mM14^{-/-}$, $M14^{-/-}$ macrophages. Data are shown as mean ± SD. Log-rank test (a) and two-way two-sided ANOVA (b,c,e,f,g) were performed for statistical analyses.

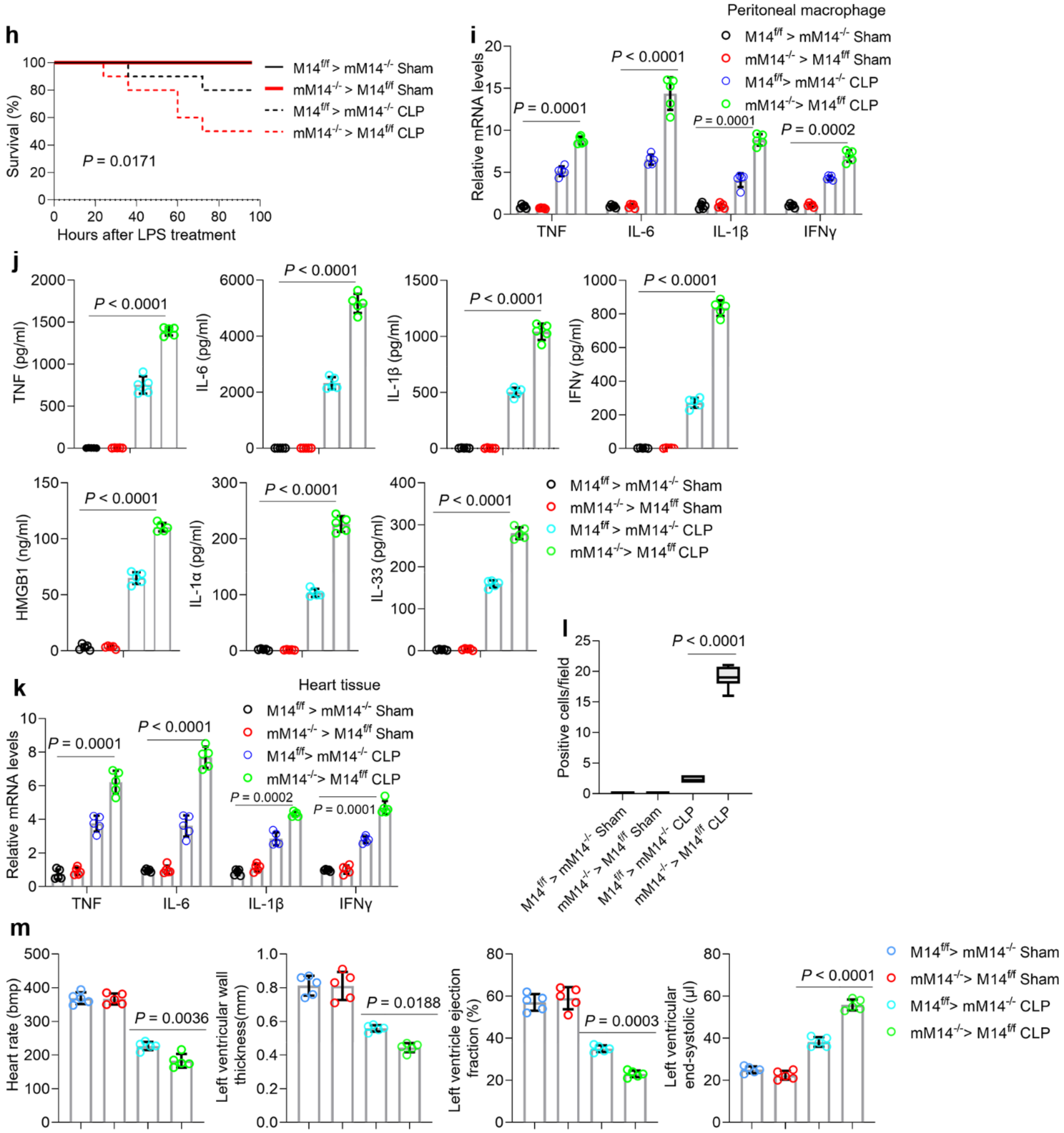

Supplementary figure 7. Roles of m^{6}A methylation in macrophages depletion and reconstitution mice in sepsis. (h-m) M14$^{f/f}$ and mM14$^{-/-}$ recipient mice were treated with clodronate liposomes to deplete macrophages and reconstituted with donor BMDMs crossly (M14$^{f/f}$ donor BMDMs > mM14$^{-/-}$ recipient mice; mM14$^{-/-}$ donor BMDMs > M14$^{f/f}$ recipient mice) prior to sham or CLP surgery for the following experiments: Kaplan-Meier survival curves (h), n = 10 in each group; Real-time PCR for cytokine expression in peritoneal macrophages (i), n = 5 in each group; Elisa detection for cytokine and DAMPs in sera (j) n = 5 in each group; Real-time PCR for cytokine expression in heart tissues (k), n = 5 in each group; Quantitative analysis of

TUNEL staining-positive cells in heart tissues displayed by box-and-whisker plot (l), n = 20 fields in each group; Measurements of Heart functions (m), n = 5 in each group. For box-and-whisker plot, bottom line means lower quartile, middle line means median, top line means upper quartile, whiskers mean minimum and maximum. mM14$^{-/-}$, M14$^{-/-}$ macrophages. Data are shown as mean ± SD. Log-rank test (h) and two-way two-sided ANOVA (i,j,k,l,m) were performed for statistical analyses.

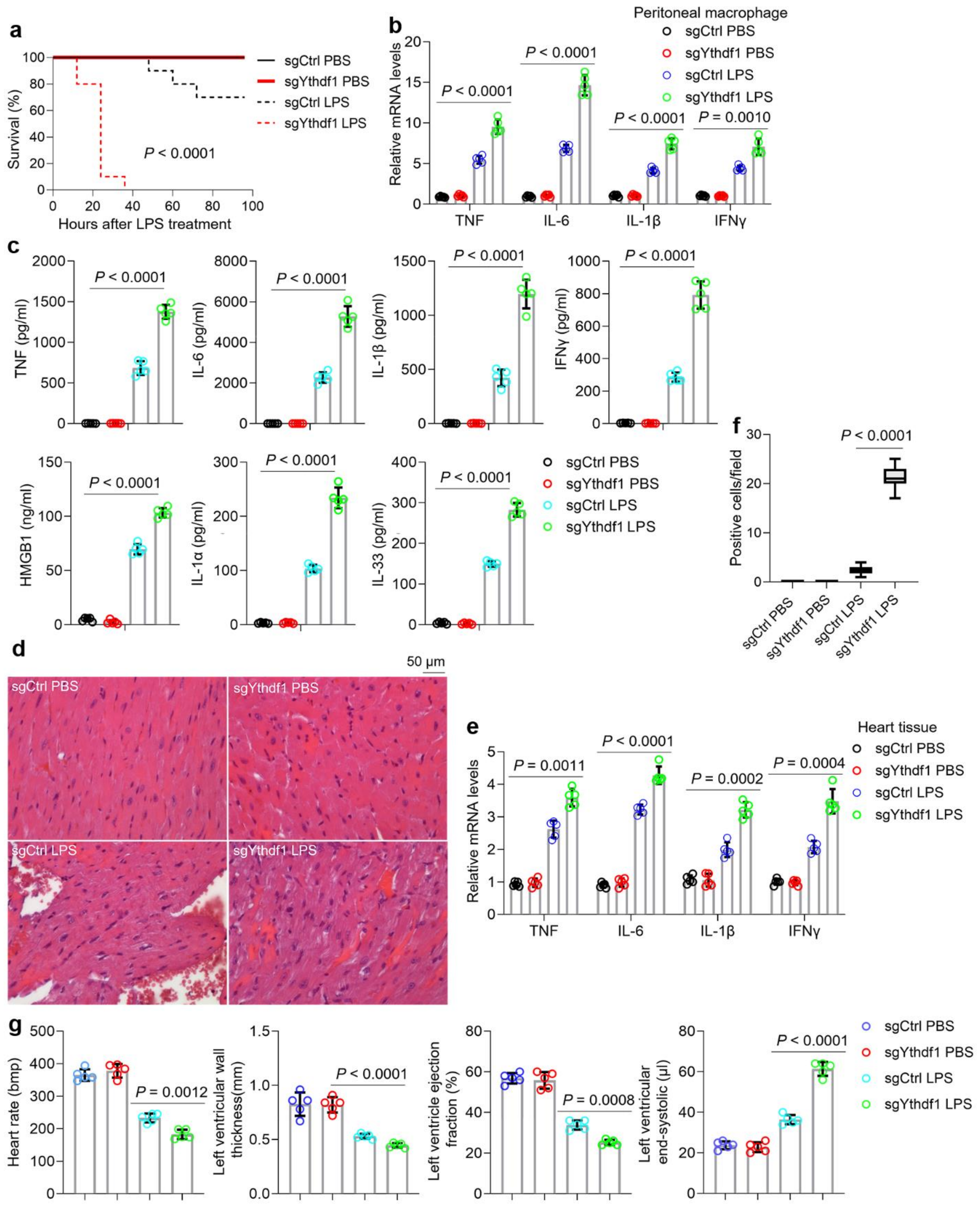

Supplementary figure 8. Functions of YTHDF1 and FTO of macrophages in sepsis. (a-g) Macrophage-depleted wild type mice were reconstituted with wild type BMDMs transduced with sgCtrl- or sg*Ythdf1*-lentivirus prior to LPS challenge for the following assays: Kaplan-Meier survival curves (a), n = 10 in each group; Real-time PCR for cytokine expression in peritoneal macrophages (b), n = 5 in each group; Elisa detection for cytokine and DAMPs in sera (c) n = 5 in each group; HE examination of

heart tissues (d), images were representative of 5 independent experiments; Real-time PCR for cytokine expression in heart tissues (e) n = 5 in each group; Quantitative analysis of TUNEL staining-positive cells in heart tissues displayed by box-and-whisker plot (f), n = 20 fields in each group; Measurements of Heart functions (g), n = 5 in each group. For box-and-whisker plot, bottom line means lower quartile, middle line means median, top line means upper quartile, whiskers mean minimum and maximum. Ctrl, Control. Data are shown as mean ± SD. Log-rank test (a) and two-way two-sided ANOVA (b,c,e,f,g) were performed for statistical analyses.

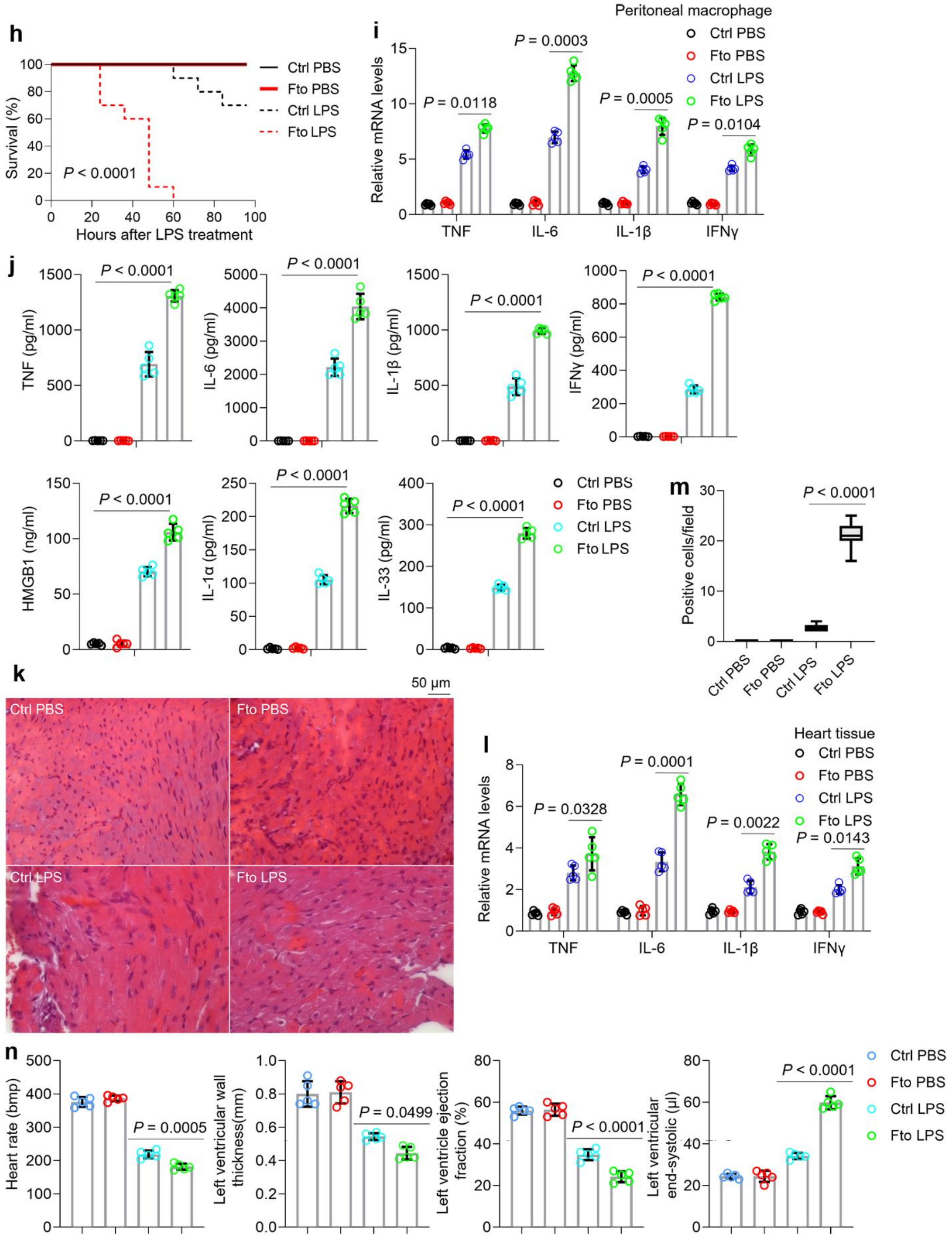

Supplementary figure 8. Functions of YTHDF1 and FTO of macrophages in sepsis. (h-n) Macrophage-depleted wild type mice were reconstituted with wild type BMDMs transduced with control- or *Fto*-lentivirus prior to LPS challenge, then the following experiments were performed: Kaplan-Meier survival curves (h), n = 10 in each group;

Real-time PCR for cytokine expression in peritoneal macrophages (i), n = 5 in each group; Elisa detection for cytokine and DAMPs in sera (j) n = 5 in each group; HE examination of heart tissues (k), images were representative of 5 independent experiments; Real-time PCR for cytokine expression in heart tissues (l), n = 5 in each group; Quantitative analysis of TUNEL staining-positive cells in heart tissues displayed by box-and-whisker plot (m), n = 20 fields in each group; Measurements of Heart functions (n), n = 5 in each group. For box-and-whisker plot, bottom line means lower quartile, middle line means median, top line means upper quartile, whiskers mean minimum and maximum. Ctrl, Control. Data are shown as mean ± SD. Log-rank test (h) and two-way two-sided ANOVA (i,j,l,m,n) were performed for statistical analyses.

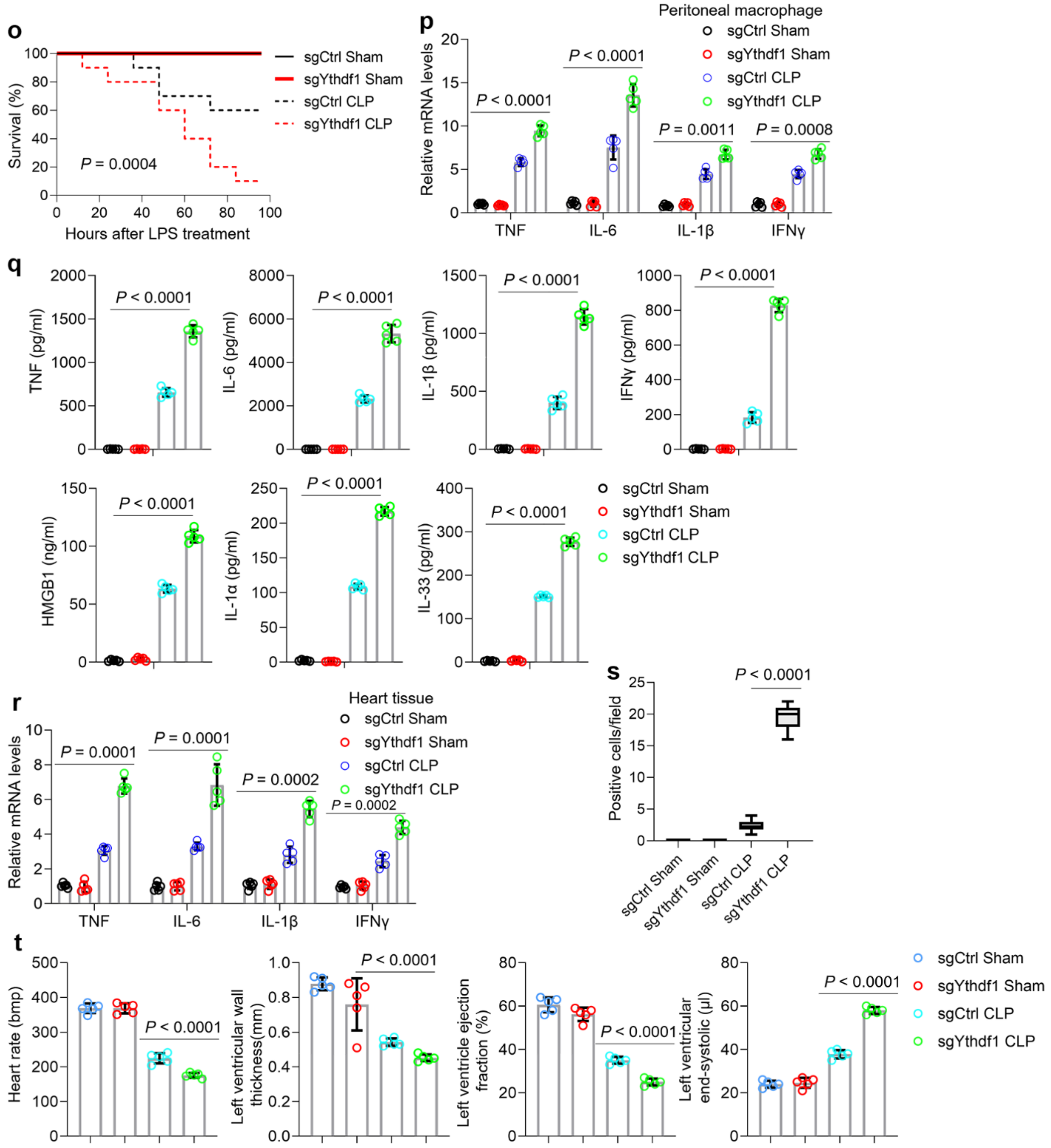

Supplementary figure 8. Functions of YTHDF1 and FTO of macrophages in sepsis. (o-t) Macrophage-depleted wild type mice were reconstituted with wild type BMDMs transduced with sgCtrl- or sg*Ythdf1*-lentivirus prior to sham or CLP surgery for the following assays: Kaplan-Meier survival curves (o), n = 10 in each group; Real-time PCR for cytokine expression in peritoneal macrophages (p), n = 5 in each group; Elisa detection for cytokine and DAMPs in sera (q) n = 5 in each group; Real-time PCR for cytokine expression in heart tissues (r), n = 5 in each group; Quantitative analysis of TUNEL staining-positive cells in heart tissues displayed by box-and-whisker plot (s), n = 20 fields each group; Measurements of Heart functions (t), n = 5 in each group.

For box-and-whisker plot, bottom line means lower quartile, middle line means median, top line means upper quartile, whiskers mean minimum and maximum. Ctrl, Control. Data are shown as mean ± SD. Log-rank test (o) and two-way two-sided ANOVA (p,q,r,s,t) were performed for statistical analyses.

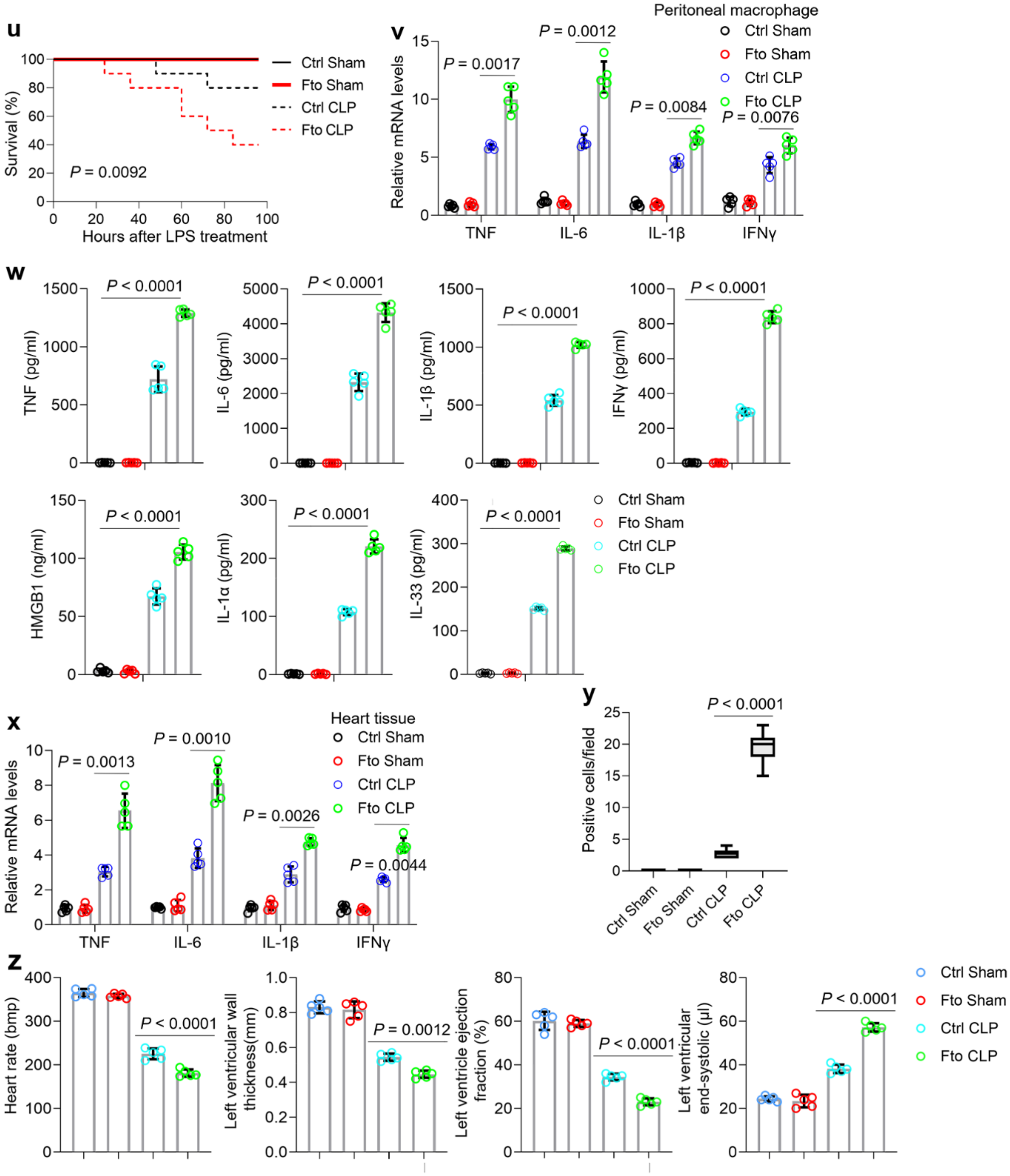

Supplementary figure 8. Functions of YTHDF1 and FTO of macrophages in sepsis. (u-z) Macrophage-depleted wild type mice were reconstituted with wild type BMDMs transduced with control- or *Fto*-lentivirus prior to sham or CLP surgery, then the following experiments were performed: Kaplan-Meier survival curves (u), n = 10 in each group; Real-time PCR for cytokine expression in peritoneal macrophages (v), n = 5 in each group; Elisa detection for cytokine and DAMPs in sera (w) n = 5 in each group; Real-time PCR for cytokine expression in heart tissues (x), n = 5 in each group; Quantitative analysis of TUNEL staining-positive cells in heart tissues displayed by box-and-whisker plot (y), n = 20 fields in each group; Measurements of Heart

functions (z), n = 5 in each group. For box-and-whisker plot, bottom line means lower quartile, middle line means median, top line means upper quartile, whiskers mean minimum and maximum. Ctrl, Control. Data are shown as mean ± SD. Log-rank test (u) and two-way two-sided ANOVA (v,w,x,y,z) were performed for statistical analyses.

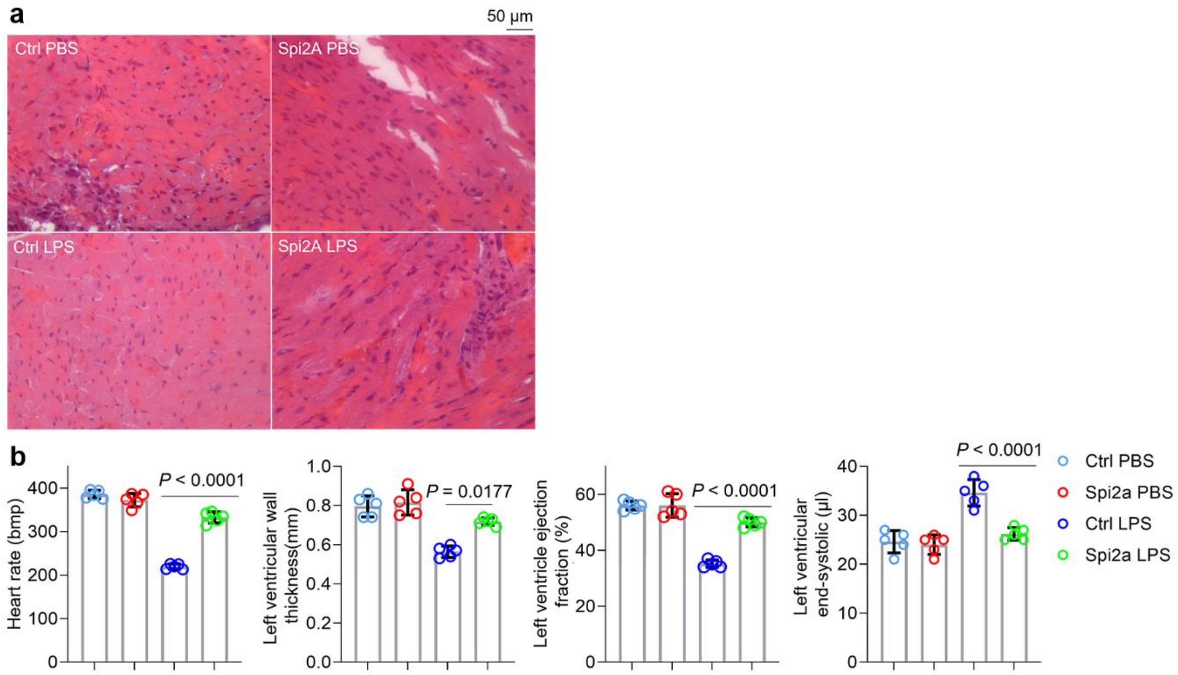

Supplementary figure 9. Forced expression of Spi2a in macrophages inhibits cytokine storm and rescues heart failure, Related to Figure 5. (a-b) Macrophage-depleted wild type mice were reconstituted with wild type BMDMs transduced with control- or *Spi2a*-lentivirus prior to LPS challenge, then the following experiments were performed: HE examination of heart tissues (a), images were representative of 5 independent experiments; Measurements of Heart functions (b), n = 5 in each group. Ctrl, Control. Data are shown as mean ± SD. Two-way two-sided ANOVA was performed for statistical analyses.

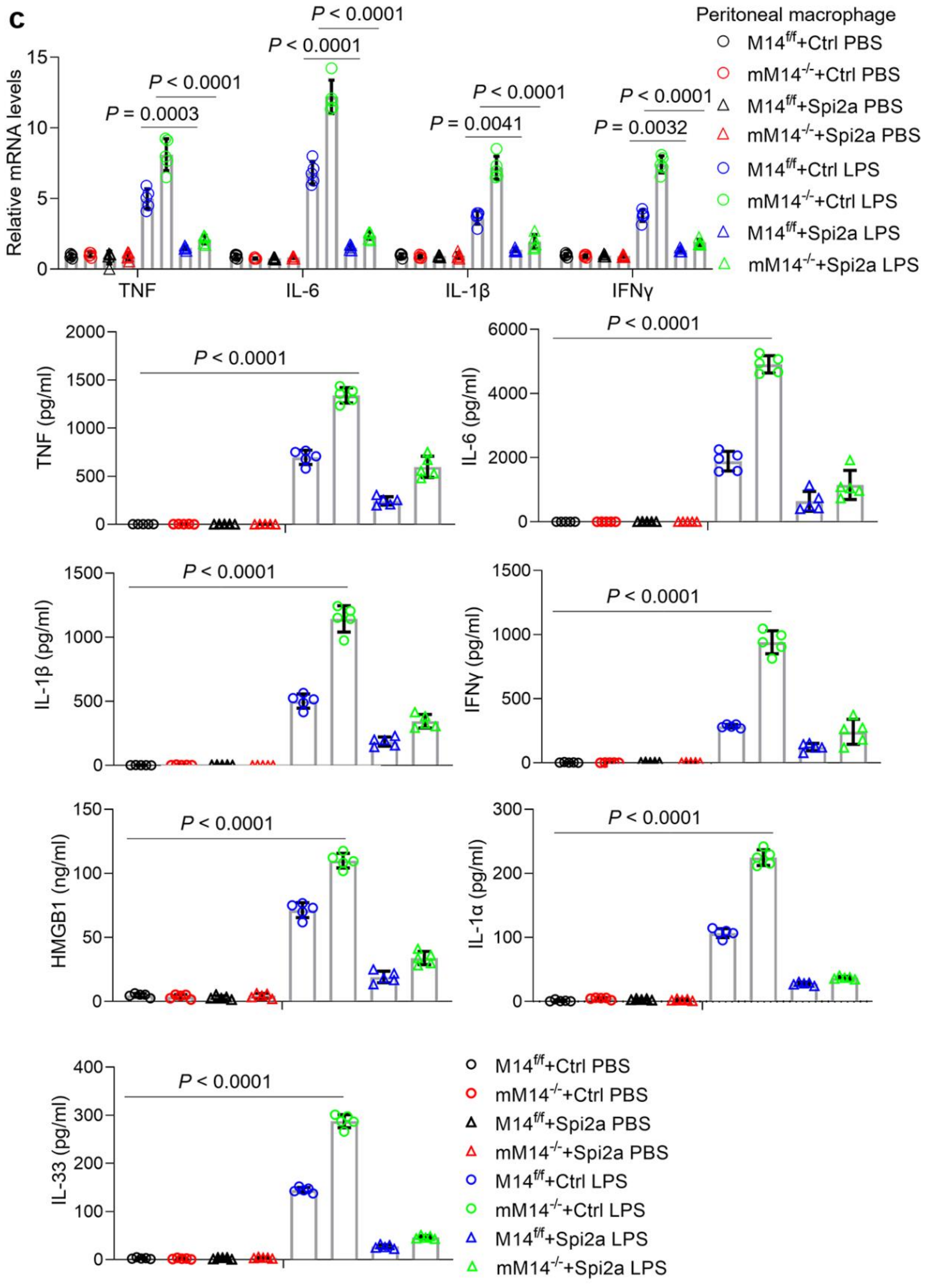

Supplementary figure 9. Forced expression of Spi2a in macrophages inhibits cytokine storm and rescues heart failure, Related to Figure 5. (c-e) Macrophage-depleted wild type mice were reconstituted with $M14^{f/f}$ or $M14^{-/-}$ BMDMs transduced with control- or *Spi2a*-overexpressing lentivirus prior to LPS challenge, then the following experiments were performed: Real-time PCR for cytokine expression in peritoneal

macrophages (c, top) and Elisa detection for cytokine and DAMPs in sera (c, bottom), n = 5 in each group. Ctrl, Control; mM14$^{-/-}$, M14$^{-/-}$ macrophages. Data are shown as mean ± SD. Two-way two-sided ANOVA was performed for statistical analyses.

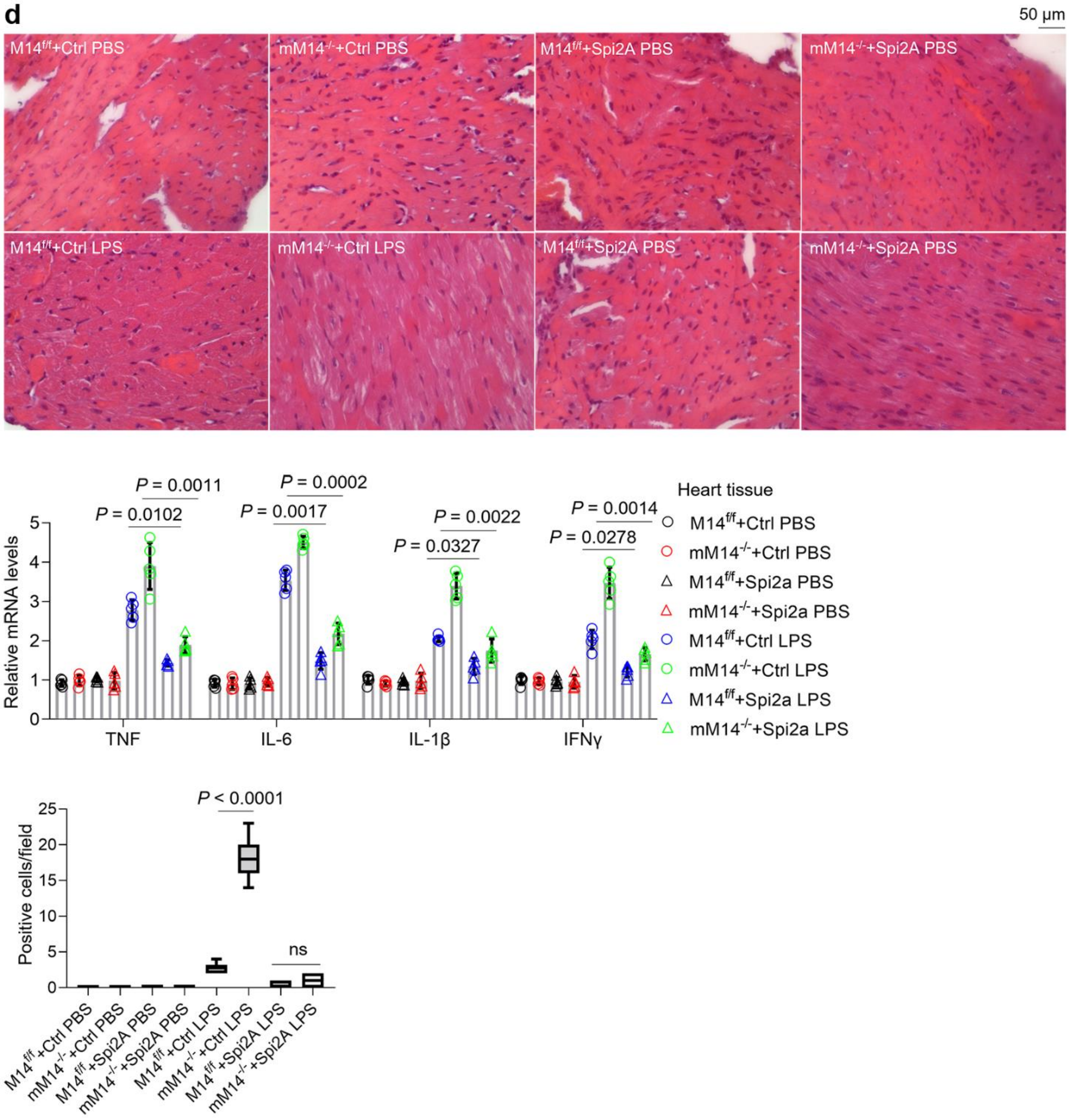

Supplementary figure 9. Forced expression of Spi2a in macrophages inhibits cytokine storm and rescues heart failure, Related to Figure 5. (d) HE examination of heart tissues (top, images were representative of 5 independent experiments), real-time PCR for cytokine expression in heart tissues (middle, n = 5 in each group) and quantitative analysis of TUNEL staining-positive cells in heart tissues displayed by box-and-whisker plot (bottom, n = 20 fields in each group). For box-and-whisker plot, bottom line means lower quartile, middle line means median, top line means upper quartile, whiskers mean minimum and maximum. Ctrl, Control; $mM14^{-/-}$, $M14^{-/-}$ macrophages. Data are shown as mean ± SD. Two-way two-sided ANOVA was performed for statistical analyses.

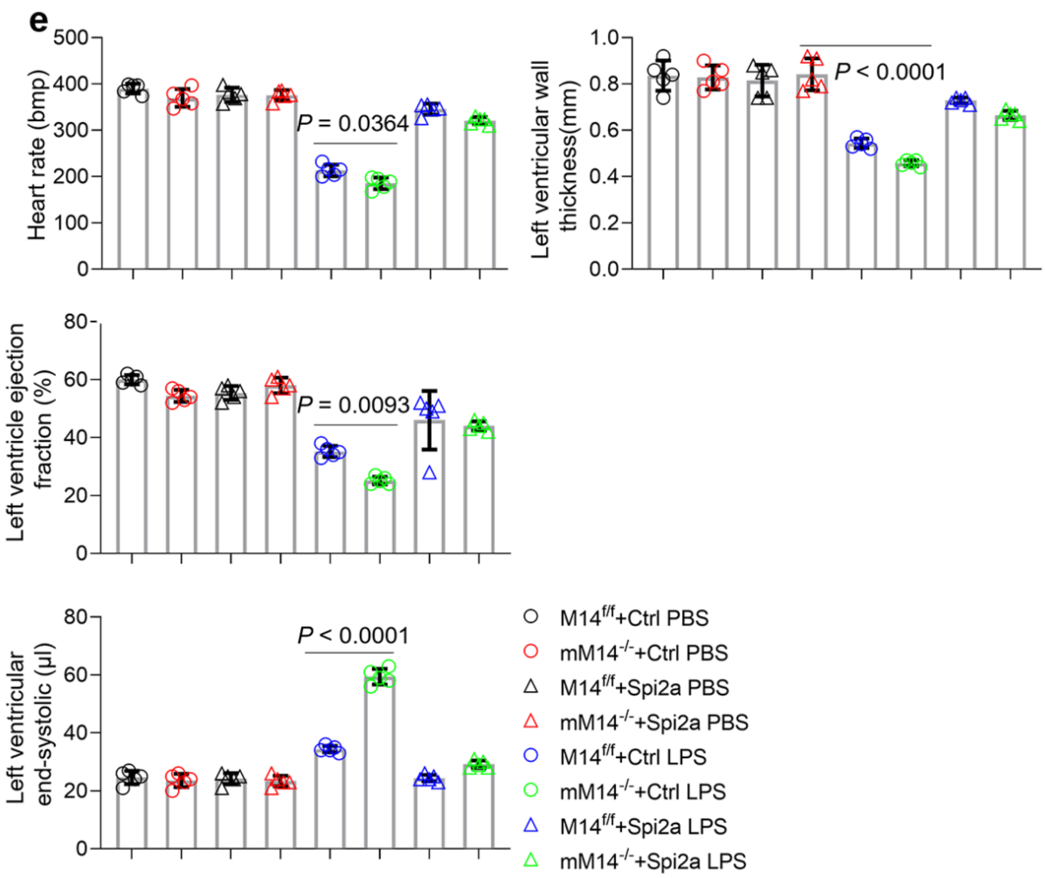

Supplementary figure 9. Forced expression of Spi2a in macrophages inhibits cytokine storm and rescues heart failure, Related to Figure 5. (e) Measurements of Heart functions, n = 5 in each group. Ctrl, Control; mM14$^{-/-}$, M14$^{-/-}$ macrophages. Data are shown as mean ± SD. Two-way two-sided ANOVA was performed for statistical analyses.

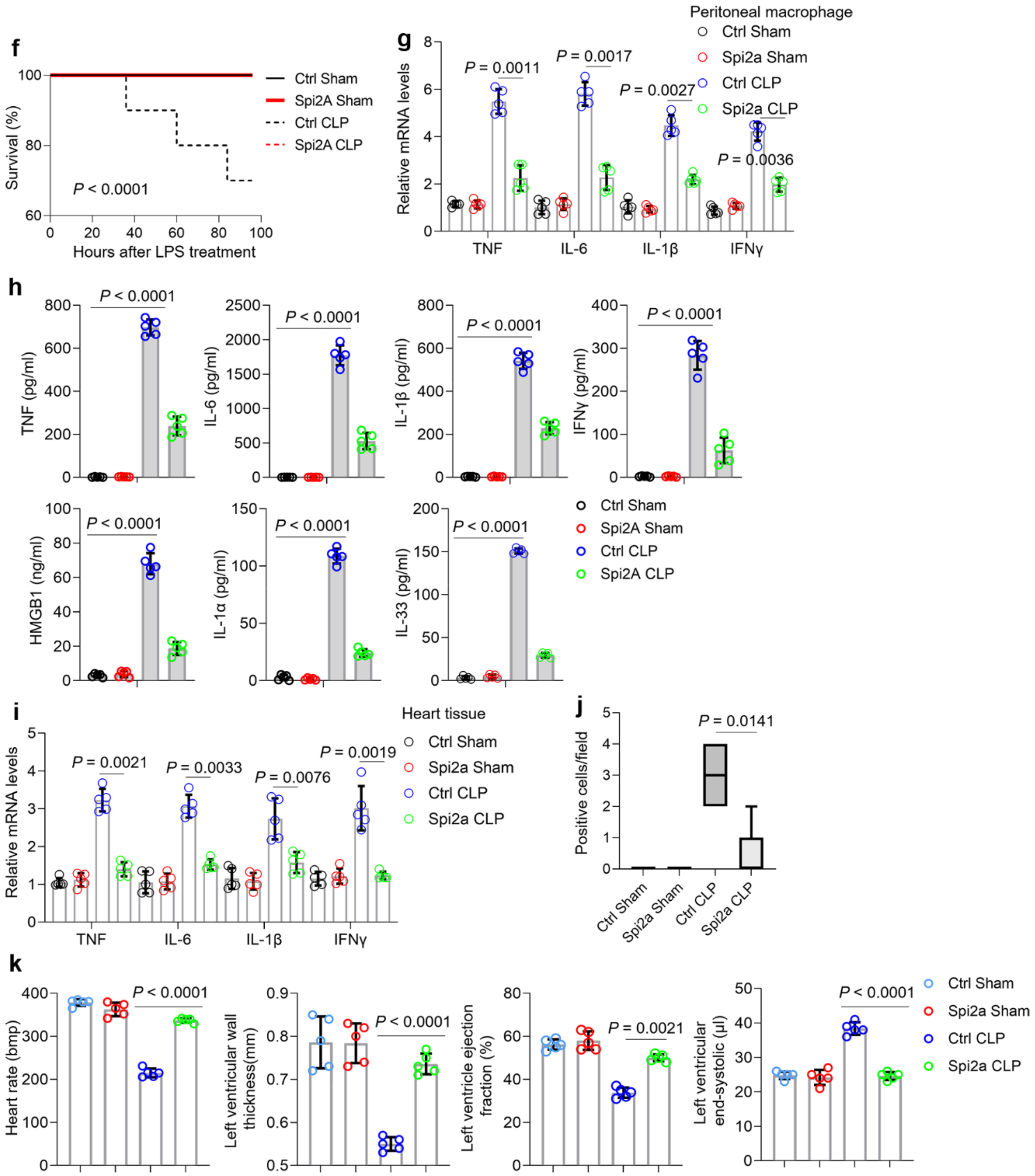

Supplementary figure 9. Forced expression of Spi2a in macrophages inhibits cytokine storm and rescues heart failure, Related to Figure 5. (f-k) Macrophage-depleted wild type mice were reconstituted with wild type BMDMs transduced with control- or *Spi2a*-lentivirus prior to sham or CLP surgery, then the following experiments were performed: Kaplan-Meier survival curves (f), n = 10 in each group; Real-time PCR for cytokine expression in peritoneal macrophages (g), n = 5 in each group; Elisa detection for cytokine and DAMPs in sera (h) n = 5 in each group; Real-time PCR for

cytokine expression in heart tissues (i), n = 5 in each group; Quantitative analysis of TUNEL staining-positive cells in heart tissues displayed by box-and-whisker plot (j), n = 20 fields in each group; Measurements of Heart functions (k), n = 5 in each group. For box-and-whisker plot, bottom line means lower quartile, middle line means median, top line means upper quartile, whiskers mean minimum and maximum. Ctrl, Control. Data are shown as mean ± SD. Log-rank test (f) and two-way two-sided ANOVA (g,h,i,j,k) were performed for statistical analyses.

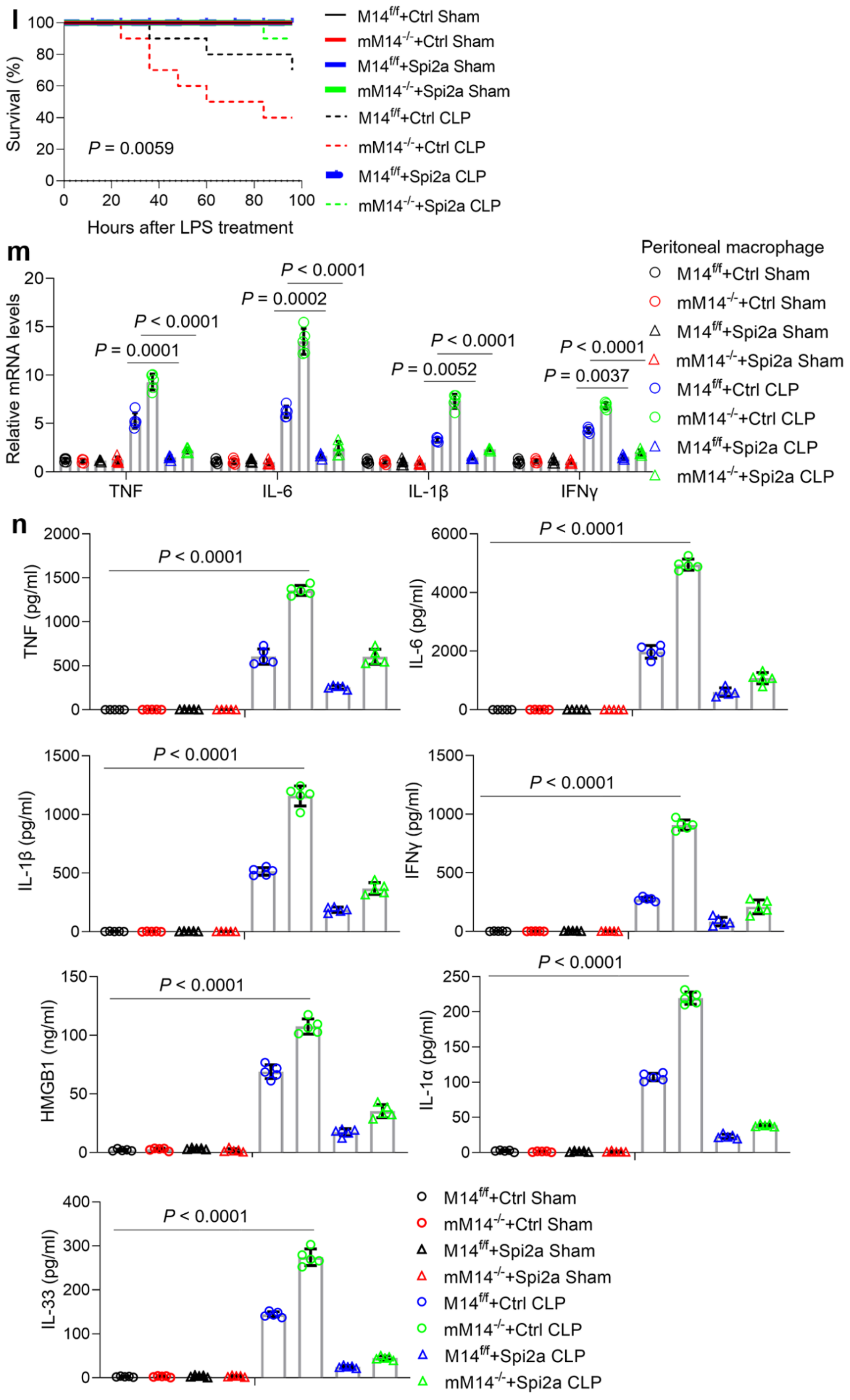
l
Survival (%)
Hours after LPS treatment
P = 0.0059
M14f/f+Ctrl Sham
mM14-/-+Ctrl Sham
M14f/f+Spi2a Sham
mM14-/-+Spi2a Sham
M14f/f+Ctrl CLP
mM14-/-+Ctrl CLP
M14f/f+Spi2a CLP
mM14-/-+Spi2a CLP
m
Relative mRNA levels
P = 0.0001
P < 0.0001
P = 0.0002
P < 0.0001
P = 0.0052
P < 0.0001
P = 0.0037
P < 0.0001
TNF
IL-6
IL-1β
IFNγ
Peritoneal macrophage
n
TNF (pg/ml)
IL-6 (pg/ml)
IL-1β (pg/ml)
IFNγ (pg/ml)
HMGB1 (ng/ml)
IL-1α (pg/ml)
IL-33 (pg/ml)
P < 0.0001

Supplementary figure 9. Forced expression of Spi2a in macrophages inhibits cytokine storm and rescues heart failure, Related to Figure 5. (l-q) Macrophage-depleted wild type mice were reconstituted with $M14^{f/f}$ or $M14^{-/-}$ BMDMs transduced with control- or *Spi2a*-lentivirus sham or CLP surgery, then the following experiments were performed: Kaplan-Meier survival curves (l), n = 10 in each group; Real-time PCR for cytokine expression in peritoneal macrophages (m), n = 5 in each group; Elisa detection for cytokine and DAMPs in sera (n), n = 5 in each group. Ctrl, Control; $mM14^{-/-}$, $M14^{-/-}$ macrophages. Data are shown as mean ± SD. Log-rank test (l) and two-way two-sided ANOVA (m,n) were performed for statistical analyses.

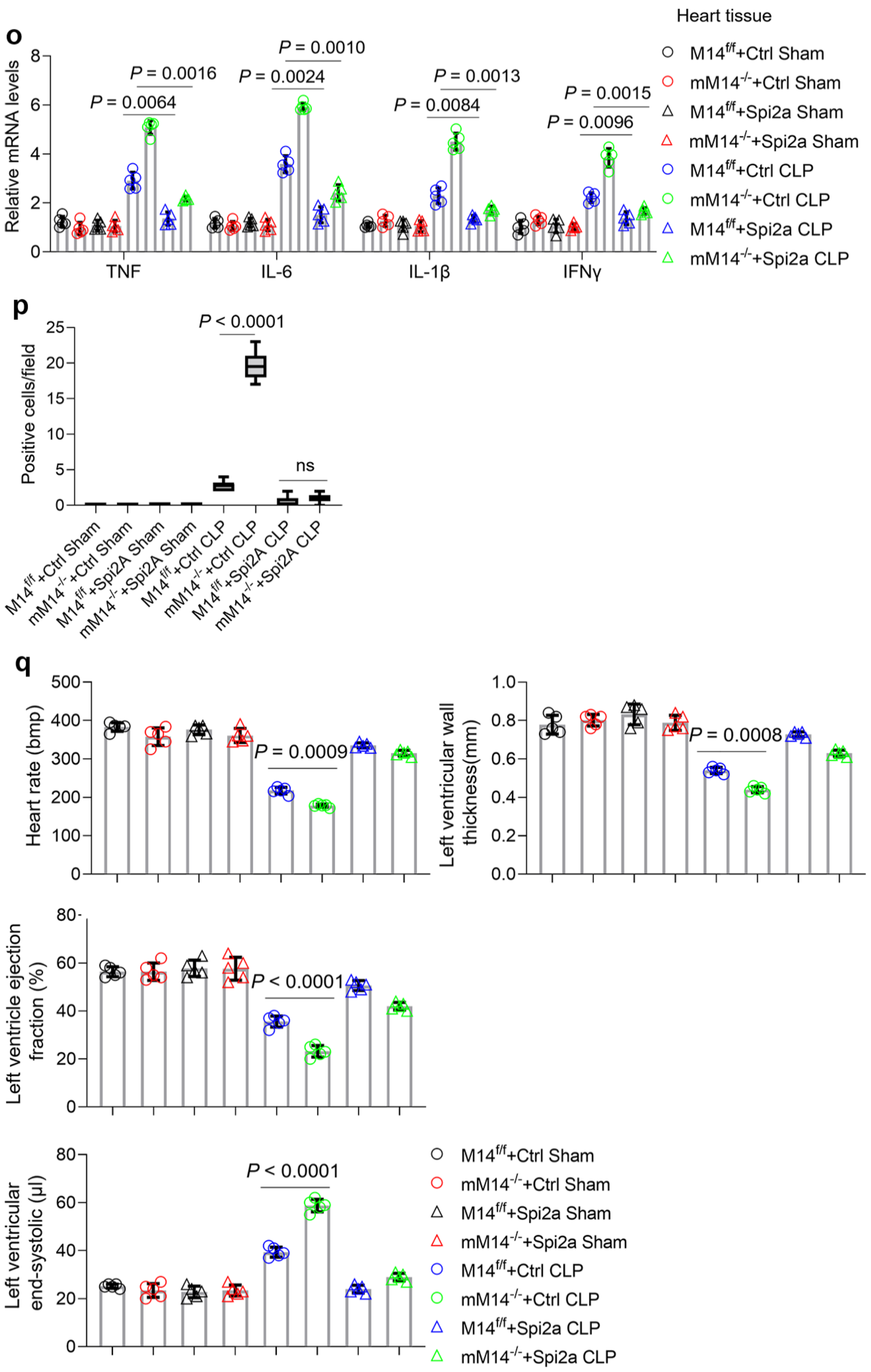
o
Heart tissue
Relative mRNA levels
P = 0.0064
P = 0.0016
P = 0.0024
P = 0.0010
P = 0.0084
P = 0.0013
P = 0.0096
P = 0.0015
TNF
IL-6
IL-1β
IFNγ
M14f/f+Ctrl Sham
mM14-/-+Ctrl Sham
M14f/f+Spi2a Sham
mM14-/-+Spi2a Sham
M14f/f+Ctrl CLP
mM14-/-+Ctrl CLP
M14f/f+Spi2a CLP
mM14-/-+Spi2a CLP
p
Positive cells/field
P < 0.0001
ns
M14f/f+Ctrl Sham
mM14-/-+Ctrl Sham
M14f/f+Spi2A Sham
mM14-/-+Spi2A Sham
M14f/f+Ctrl CLP
mM14-/-+Ctrl CLP
M14f/f+Spi2A CLP
mM14-/-+Spi2a CLP
q
Heart rate (bmp)
P = 0.0009
Left ventricular wall thickness(mm)
P = 0.0008
Left ventricle ejection fraction (%)
P < 0.0001
Left ventricular end-systolic (μl)
P < 0.0001
M14f/f+Ctrl Sham
mM14-/-+Ctrl Sham
M14f/f+Spi2a Sham
mM14-/-+Spi2a Sham
M14f/f+Ctrl CLP
mM14-/-+Ctrl CLP
M14f/f+Spi2a CLP
mM14-/-+Spi2a CLP

Supplementary figure 9. Forced expression of Spi2a in macrophages inhibits cytokine storm and rescues heart failure, Related to Figure 5. Real-time PCR for cytokine expression in heart tissues (o), n = 5 in each group; Quantitative analysis of TUNEL staining-positive cells in heart tissues displayed by box-and-whisker plot (p), n = 20 fields in each group; Measurements of Heart functions (q), n = 5 in each group. For box-and-whisker plot, bottom line means lower quartile, middle line means median, top line means upper quartile, whiskers mean minimum and maximum. Ctrl, Control; $mM14^{-/-}$, $M14^{-/-}$ macrophages. Data are shown as mean ± SD. Two-way two-sided ANOVA was performed for statistical analyses.

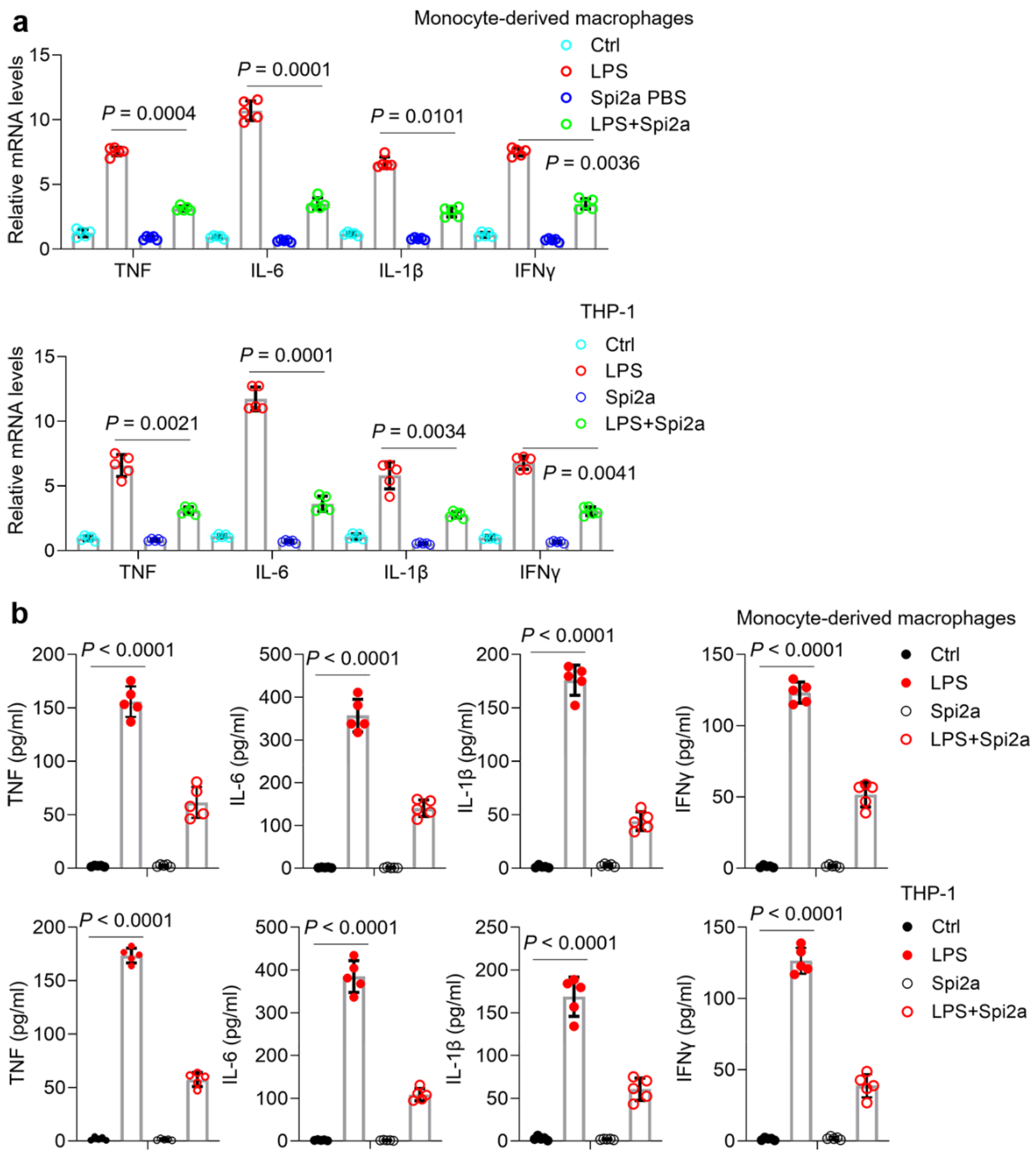

Supplementary figure 10. SERPINA3 plays an essential role in suppressing activated macrophages-driving cytokines, Related to Figure 9 and Figure 10. (a) Real-time PCR for cytokines in control- or *Spi2a*-lentivirus-infected monocyte-derived macrophages (top) or THP-1 cells (bottom) with or without LPS treatment. (b) Elisa detection of cytokines in the culture medium of control- or *Spi2a*-lentivirus-infected monocyte-derived macrophages (top) or THP-1 cells (bottom) with or without LPS treatment. Primary macrophages were from 5 different human donors. Cells were all treated with 100 ng/ml LPS for 8 hours. Ctrl, Control. Data are shown as mean ± SD. Two-way two-sided ANOVA was performed for statistical analyses.

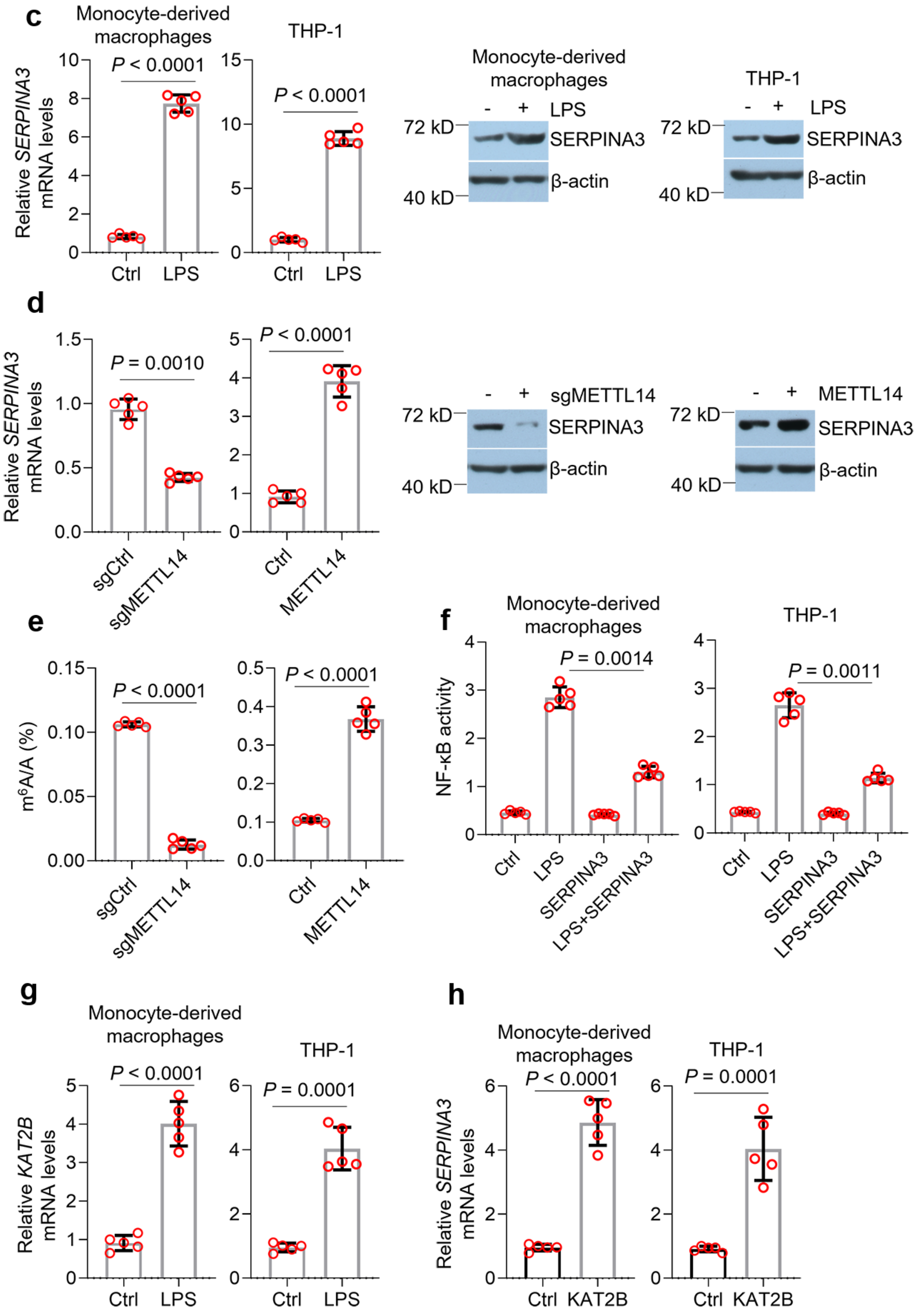

Supplementary figure 10. SERPINA3 plays an essential role in suppressing activated macrophages-driving cytokines, Related to Figure 9 and Figure 10. (c) Real-time PCR and western blot showing SERPINA3 levels in monocyte-derived macrophages or THP-1 cells with or without LPS treatment. (d) Real-time PCR and western blot

showing SERPINA3 levels in monocyte-derived macrophages or THP-1 cells with sg*METTL14*- or *METTL14*-lentivirus infection. (e) m^6A quantification of monocyte-derived macrophages with sg*METTL14*- or *METTL14*-lentivirus infection. (f) NF-κB activity assays of control- or *SERPINA3*-lentivirus-infected monocyte-derived macrophages or THP-1 cells with or without LPS treatment. (g) Real-time PCR for *KAT2B* levels in monocyte-derived macrophages or THP-1 cells with or without LPS treatment. (h) Real-time PCR for SERPINA3 levels in monocyte-derived macrophages or THP-1 cells with or without *KAT2B*-lentivirus infection. Primary macrophages were from 5 different human donors. Cells were all treated with 100 ng/ml LPS for 8 hours. Ctrl, Control. Data are shown as mean ± SD. Unpaired two-tailed Student's *t* test (c,d,e,g,h) and two-way two-sided ANOVA (f) were performed for statistical analyses.

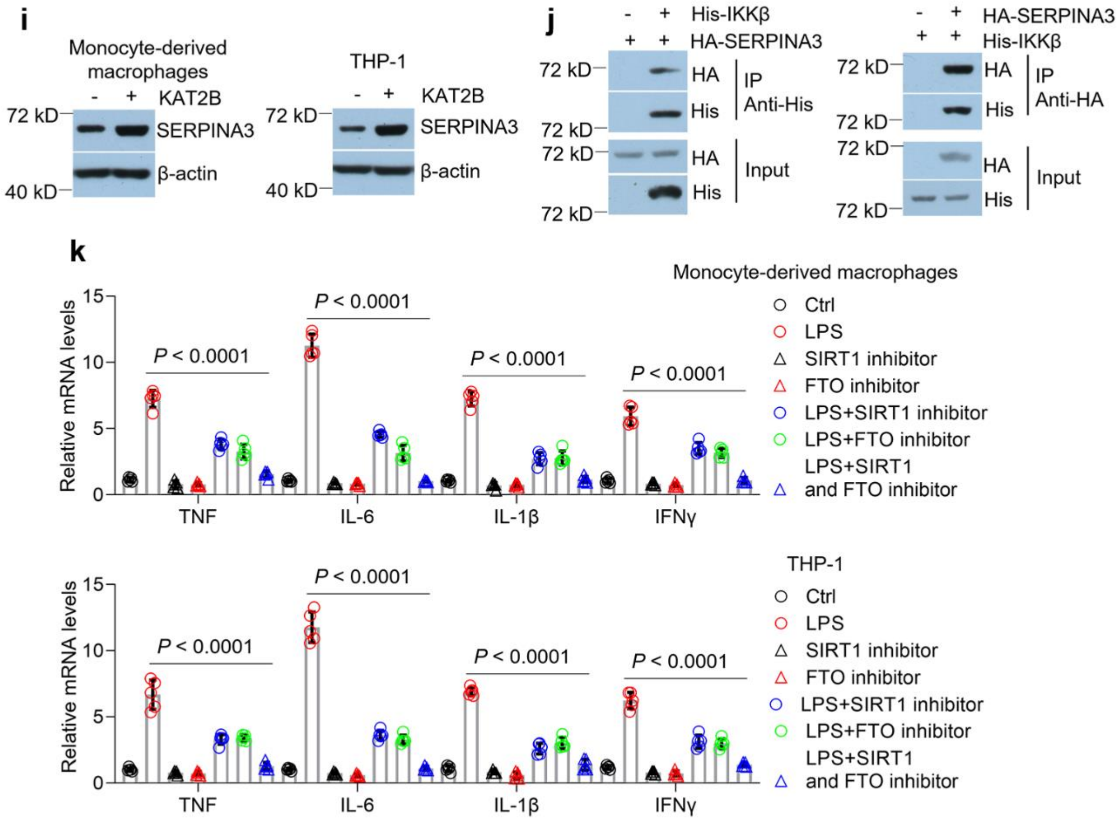
i
Monocyte-derived macrophages
- + KAT2B
72 kD
SERPINA3
β-actin
40 kD
THP-1
- + KAT2B
72 kD
SERPINA3
β-actin
40 kD
j
- + His-IKKβ
+ + HA-SERPINA3
72 kD
HA
His
IP Anti-His
72 kD
72 kD
HA
His
Input
72 kD
- + HA-SERPINA3
+ + His-IKKβ
72 kD
HA
His
IP Anti-HA
72 kD
72 kD
HA
His
Input
72 kD
k
Monocyte-derived macrophages
Ctrl
LPS
SIRT1 inhibitor
FTO inhibitor
LPS+SIRT1 inhibitor
LPS+FTO inhibitor
LPS+SIRT1 and FTO inhibitor
Relative mRNA levels
15
10
5
0
P < 0.0001
P < 0.0001
P < 0.0001
P < 0.0001
TNF
IL-6
IL-1β
IFNγ
THP-1
Ctrl
LPS
SIRT1 inhibitor
FTO inhibitor
LPS+SIRT1 inhibitor
LPS+FTO inhibitor
LPS+SIRT1 and FTO inhibitor
Relative mRNA levels
15
10
5
0
P < 0.0001
P < 0.0001
P < 0.0001
P < 0.0001
TNF
IL-6
IL-1β
IFNγ

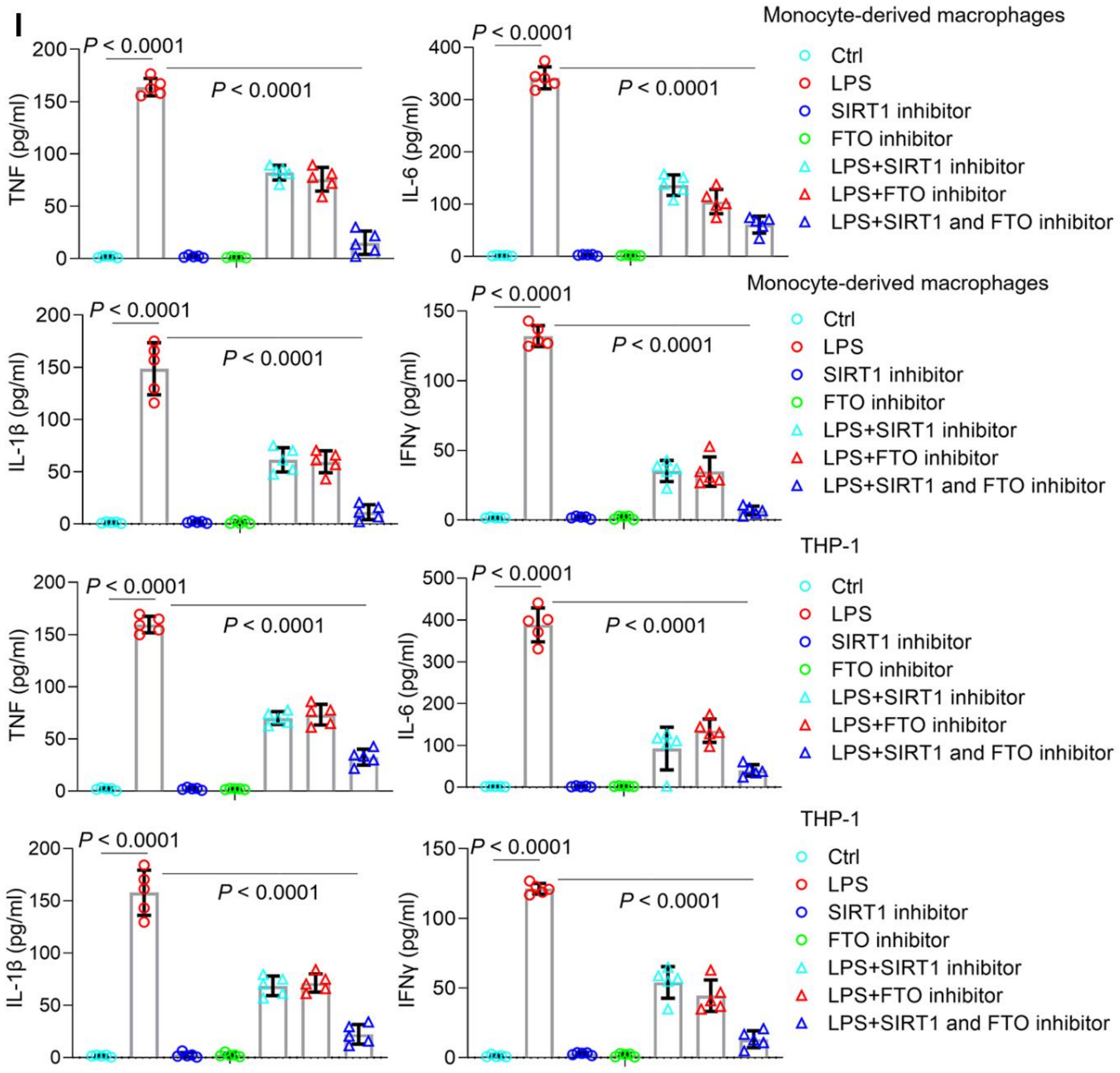
I
Monocyte-derived macrophages
TNF (pg/ml)
IL-6 (pg/ml)
IL-1β (pg/ml)
IFNγ (pg/ml)
THP-1
P < 0.0001
Ctrl
LPS
SIRT1 inhibitor
FTO inhibitor
LPS+SIRT1 inhibitor
LPS+FTO inhibitor
LPS+SIRT1 and FTO inhibitor

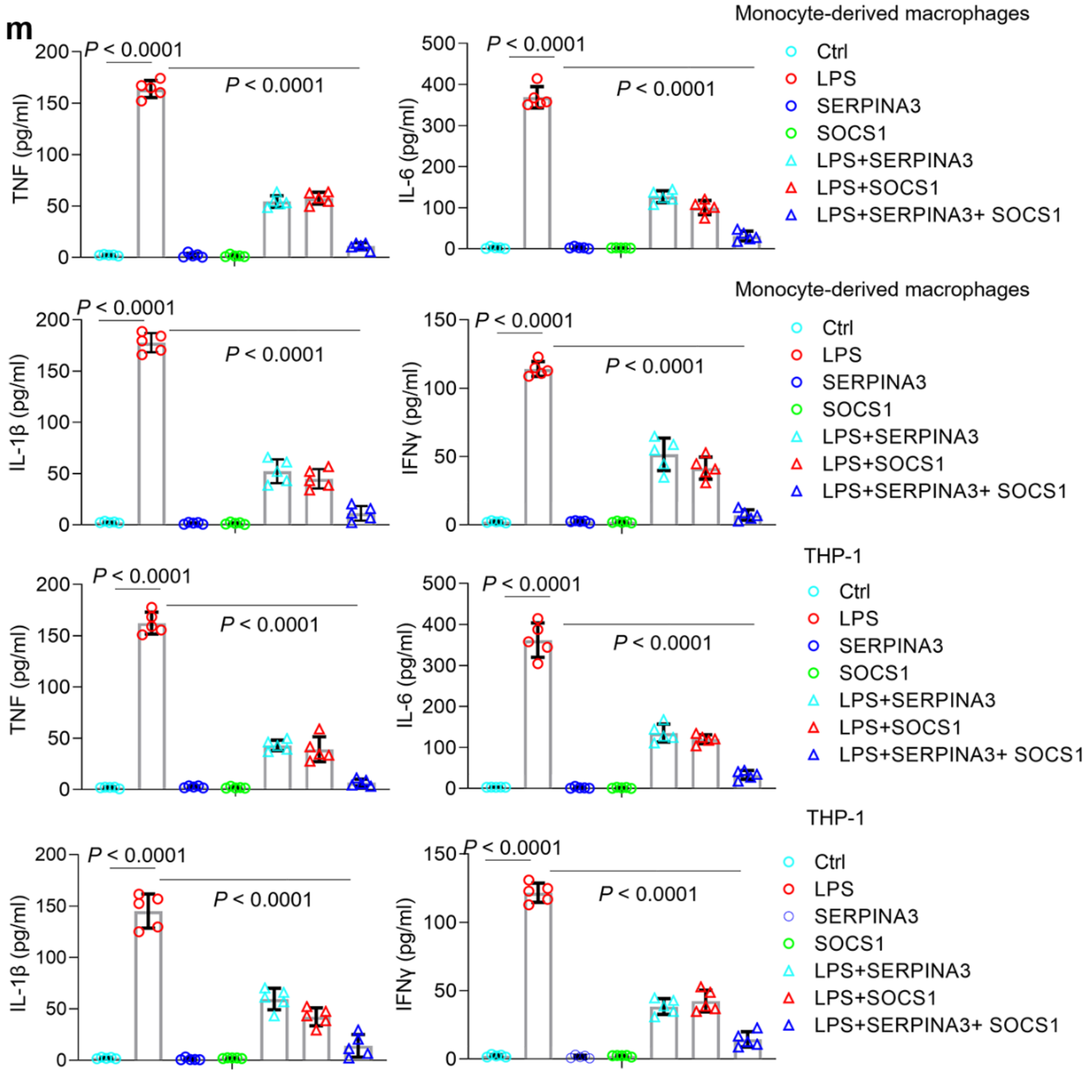

Supplementary figure 10. SERPINA3 plays an essential role in suppressing activated macrophages-driving cytokines, Related to Figure 9 and Figure 10. (i) Western blot for SERPINA3 levels in monocyte-derived macrophages or THP-1 cells with or without *KAT2B*-lentivirus infection. (j) Monocyte-derived macrophages were transduced with HA-*SERPINA3*- and His-*IKKβ*-lentivirus as indicated, followed by Co-IP against His (left) or HA (right) antibody and western blot analyses. (k-l) Monocyte-derived macrophages or THP-1 cells were pretreated with 10 μM FTO and/or 1 μM SIRT1 inhibitors for 4 hours, followed by PBS or LPS treatment. RNAs were harvested for real-time PCR analysis (k) and culture media were collected for Elisa assays (l). (m) Elisa evaluation for cytokine in PBS- or LPS-treated monocyte-derived macrophages or THP-1 cells with or without *SERPINA3*- or *SOCS1*-lentivirus infection as indicated. Primary macrophages were from 5 different human donors. Cells were all treated with 100 ng/ml LPS for 8 hours. Ctrl, Control. Data are shown as mean ± SD. Two-way two-sided ANOVA were performed for statistical analyses.

Supplementary Table 1

Sequences of primers involved in the study

**RT-PCR primers**		
Name	Forward 5'-3'	Reverse 5'-3'
mSpi2a	CTTCCCAACGGCTGGAATCTA	ACTGTCCAATCAGGCATAGCG
mMettl14	GCTGAGAGTGCGGATAGCAT	CCTTCATCCAGACACTTCCGT
mMettl3	GGACACGTGGAGCTCTATCC	GCACGGGACTATCACTACGG
mWtap	AGTTATGGCACGGGATGAGT	TCCCTTAAACCAGTCACATCGT
mFto	TCAGCAGTGGCAGCTGAAAT	TCACCACGTCCCGAAACAAG
mAlkbh5	CGGGAGAAGCTCAAGTCCAT	TATTTCCGCTTGGTGGTCCC
mKat2b	GAAGCCGCCATTTGAGAAGC	AGTTGATGCGGTTCAGAAACA
mTnfα	TCAGCCTCTTCTCATTCCTG	CAGGCTTGTCACTCGAATTT
mIl-6	CCTCTCTGCAAGAGACTTCCA	AGAATTGCCATTGCACAACTCT
mIl-1β	CCAAAAGATGAAGGGCTGCT	ACAGAGGATGGGCTCTTCTT
mIfnγ	GCGTCATTGAATCACACCTG	TGAGCTCATTGAATGCTTGG
mSerpina3f	AGCCCAGGTTGATAAATGAGC	TGGGGAAAAGCATACTCCACA
mGapdh	AGGTCGGTGTGAACGGATTTG	TGTAGACCATGTAGTTGAGGTCA
hTNFα	CCTCTCTCTAATCAGCCCTCTG	GAGGACCTGGGAGTAGATGAG
hIL-6	ACTCACCTCTTCAGAACGAATTG	CCATCTTTGGAAGGTTCAGGTTG
hIL-1β	ATGATGGCTTATTACAGTGGCAA	GTCGGAGATTCGTAGCTGGA
hIFNγ	TCGGTAACTGACTTGAATGTCCA	TCGCTTCCCTGTTTTAGCTGC
hSERPIMA3	CCTGAAGGCCCCTGATAAGAA	GCTGGACTGATTGAGGGTGC
hGAPDH	GGAGCGAGATCCCTCCAAAAT	GGCTGTTGTCATACTTCTCATGG
**RIP-qPCR and CLIP-qPCR primers**		
Target site	Forward 5'-3'	Reverse 5'-3'
Spi2a IP-seq	CTAAGAAAGGAACCTGGTGCCCTGG	AGTGCTACAGGGGGAGGAGCTGCTCT
**Lentivirus and plasmids construction primers**		
Name	Forward 5'-3'	Reverse 5'-3'
mIkkβ cDNA	ATGAGCTGGTCACCGTCCCT	AGTCAGTGTCCGGACGAGGTCCGA
mMettl3 cDNA	ATGTCGGACACGTGGAGCTCTAT	GATATTTAAGAATCCAAATC
mMettl14 cDNA	ATGGATAGCCGCCTGCAGGAGAT	GATGGCTCCTCATTTCGGCGGAGA
mKat2a cDNA	ATGGCGGAACCTTCCCAGGCCCCA	GATGAACAGCTACTCGGGAGGGA
mKat2b cDNA	ATGGCCGAGGCTGGCGGGGCC	AGTGAACAGTTAGTTGGGACG
mSirt1 cDNA	ATGGCGGACGAGGTGGCGCTCGC	AATACTAAACAGACTACCTA

mSirt2 cDNA	ATGGCCGAGCCGGACCCCTC	AATGACGACAAGGAGAAAGA
mSirt6 cDNA	ATGTCGGTGAATTATGCAGCA	AGTCGACCCCCGTCGGAGCCAGAA
mSirt7 cDNA	ATGGCAGCCGGTGGCGGTCTGA	GATACGGTGAAAGAAGGAAAA
mSpi2a cDNA	ATGGCTGGTGTCTCCCCTGCTGT	AGTGACAGGTTAGTCCGTATCG
mFto cDNA	ATGAAGCGCGTCCAGACCGC	GATCCTAGAACGAAGGTCGT
mSerpina3f cDNA	ATGGCTGGTGTCTCCCCTGC	AGTGACAGATCAGTCTGTACC
hMETTL14 cDNA	ATGGATAGCCGCTTGCAGGAGATCC	AATAGCTCCACCTTTCGGTGGAGA
hSOCS1 cDNA	ATGGTAGCACACAACCAGGTGGCA	AGTTTAGACCTTCCCCTTCCTC
hIKKβ cDNA	ATGAGCTGGTCACCTTCCCTGACA	AGTACTCCGGACGAGGTCCGTCGAC
hKAT2B cDNA	ATGTCCGAGGCTGGCGGGGCCGG	AGTGAACAGTTAATTAGGTCG
hSERPINA3 cDNA	ATGGAGAGAATGTTACCTCTCC	TCACCAATCCCAAGCAAGCCTAG
M14-R298P mutation	AACTGTGAAGCCTAGCACAGACG	CCTTTGATCCCCATGAGG
M3-D394A mutation	AGCTTCTAGAGCCACCATGTACCCATACGATGTTCCAGATTACGCTTCGGACACGTGGAGCTCTAT	ATATCCGCGGGTGGGGCAGCCATCACAACTGCAAA
M3-W397A mutation	GCCCCACCCGCGGATATTCACATGGAACTGCCCTAT	AATTGCGGCCGCCTATAAATTCTTAGGTTTAG
pGL3-Spi2a plasmid	CTAAGAAAGGAACCTGGTGCCCTGGG	AGTGCTACAGGGGGAGGAGCTGCTCTGA
pGL3-Spi2a mutation	CTAAGAAAGGATCCTGGTGCCC	GGGCACCAGGATCCTTTCTTAG
pLV-Neo-EF1A-His-mMettl14-K13R	CGGCAGAGATTACGGCGGCAG	CCGTAATCTCTGCCGCTCCCG
pLV-Neo-EF1A-His-mMettl14-K38R	AATAGCAGAGATGAACAGAGG	TTCATCTCTGCTATTTAACAC
pLV-Neo-EF1A-His-mMettl14-K63R	AACTCAAGACGGAAGTGTCTG	CTTCCGTCTTGAGTTTGGAGC
pLV-Neo-EF1A-His-mMettl14-K65R	CGGAGATGTCTGGATGAAGGA	CAGACATCTCCGTTTTGAGTT
pLV-Neo-EF1A-His-mMettl14-K7	GAAGACAGAGTAGAAGAATAT	TTCTACTCTGTCTTCATCAGT

6R		
pLV-Neo-EF1A-His-mMettl14-K81R	GAATATAGAGATGAACTGGAA	TTCATCTCTATATTCTTCTAC
pLV-Neo-EF1A-His-mMettl14-K101R	ATTTACAGAGATTCCAGTACC	GGAATCTCTGTAAATCTCTTC
pLV-Neo-EF1A-His-mMettl14-K108R	TTTCTTAAGGGAACGCAGAGC	CGTTCCTCTAAGAAAGGTACT
pLV-Neo-EF1A-His-mMettl14-K148R	TACCCTAGACTTAGGGAACTC	CCTAAGTCTAGGGTATTCTTC
pLV-Neo-EF1A-His-mMettl14-K156R	AGACTAAGAGATGAGTTAATA	CTCATCTCTTAGTCTGATGAG
pLV-Neo-EF1A-His-mMettl14-K162R	ATAGCTAGATCAAACACTCCT	GTTTGATCTAGCTATTAACTC
pLV-Neo-EF1A-His-mMettl14-K185R	ACACCCAGATTTGATGTGATT	ATCAAATCTGGGTGTCAATTC
pLV-Neo-EF1A-His-mMettl14-K209R	AATGAGAGATGCTGGACCTGG	CCAGCATCTCTCATTCGCAGT
pLV-Neo-EF1A-His-mMettl14-K218R	ATTATGAGATTAGAAATCGAT	TTCTAATCTCATAATATCATC
pLV-Neo-EF1A-His-mMettl14-K250R	TTGCGAAGATGGGGTTACAGA	ACCCCATCTTCGCAAGCATAC
pLV-Neo-EF1A-His-mMettl14-K263R	TGGATTAGAACCAATAAAAAC	ATTGGTTCTAATCCAACAAAT
pLV-Neo-EF1A-His-mMettl14-K266R	ACCAATAGAAACAATCCTGGA	ATTGTTTCTATTGGTTTTAAT
pLV-Neo-EF1A-His-mMettl14-K271R	CCTGGAAGAACAAAGACTCTA	CTTTGTTCTTCCAGGATTGTT
pLV-Neo-EF1A-His-mMettl14-K273R	AAGACAAGAACTCTAGATCCA	TAGAGTTCTTGTCTTTCCAGG
pLV-Neo-EF1A-	AGTCCAAGAGCAGTTTTCCAG	AACTGCTCTTGGATCTAGAGT

His-mMettl14-K278R		
pLV-Neo-EF1A-His-mMettl14-K285R	AGAACAAGAGAGCATTGCCTG	ATGCTCTCTTGTTCTCTGGAA
pLV-Neo-EF1A-His-mMettl14-K293R	ATGGGGATCAGAGGAACCGTG	GGTTCCTCTGATCCCCATCAG
pLV-Neo-EF1A-His-mMettl14-K297R	ACCGTGAGACGAAGCACAGAC	TGTGCTTCGTCTCACGGTTCC
pLV-Neo-EF1A-His-mMettl14-K326R	ATAGAAAGACCAGTAGAAATT	TACTGGTCTTTCTATATTGCC
pLV-Neo-EF1A-His-mMettl14-K398R	CGACCGAGATCACCTCCTCCC	AGGTGATCTCGGTCGAAGCCT
pLV-Neo-EF1A-His-mMettl14-K398Q	CGACCGCAATCACCTCCTCCC	AGGTGATTGCGGTCGAAGCCT
pLV-Neo-EF1A-His-mMettl14-K403R	CCTCCCAGATCCAAGTCTGAC	CTTGGATCTGGGAGGAGGTGA
pLV-Neo-EF1A-His-mMettl14-K405R	AAGTCCAGATCTGACCGTGGG	GTCAGTCTTGGACTTGGGAGG

Supplementary Table 2

Clinical parameters of all sepsis patients (n = 15)

Age	66.53 ± 15.87 y
Gender	8 males and 7 females
Respiratory frequency	20.27 ± 1.94 breaths/min
Cardiac frequency	87.60 ± 14.89 pulsations/min
Hematocrit	31.80 ± 8.47
Creatinine	129.41 ± 130.15 µmol/l
Leukocytes	1.77 ± 0.74 (x 10^9/l)
Neutrophiles	6.51 ± 5.43 (x 10^9/l)
K^+	4.37 ± 0.62 mmol/l
Na^+	138.85 ± 4.23 mmol/l
Glucose	6.52 ± 2.37 mmol/l
C-Reactive Protein (CRP)	59.41 ± 38.89 mg/l
Troponin T, High Sensitivity (hs-TnT)	0.24 ± 0.48 pg/ml
SOFA (Sequential [Sepsis-Related] Organ Failure Assessment) score	3.73 ± 0.59

*CRP is a critical marker for inflammation and hs-TnT is a typical marker for myocardial dysfunction in clinic. The normal range of CRP or hs-TnT in healthy individuals is less than 6 mg/l or 0.014 pg/ml, respectively. For healthy individuals, there are 8 males and 7 females in total and the average age is 66.13 ± 15.41.

Supplementary Table 3

Critical clinical parameters analyses between male and female patients.

	Male (n = 8)	Female (n = 7)	*P* value
C-Reactive Protein (CRP)	58.52 ± 36.57	55.37 ± 44.41	0.8873
Troponin T, High Sensitivity (hs-TnT)	0.33 ± 0.67	0.16 ± 0.29	0.5456
SOFA (Sequential [Sepsis-Related] Organ Failure Assessment) score	3.86 ± 0.37	3.71 ± 0.76	0.6627

Unpaired two-tailed student’s *t* test was used.

Uncropped versions of blots

Figure 1a

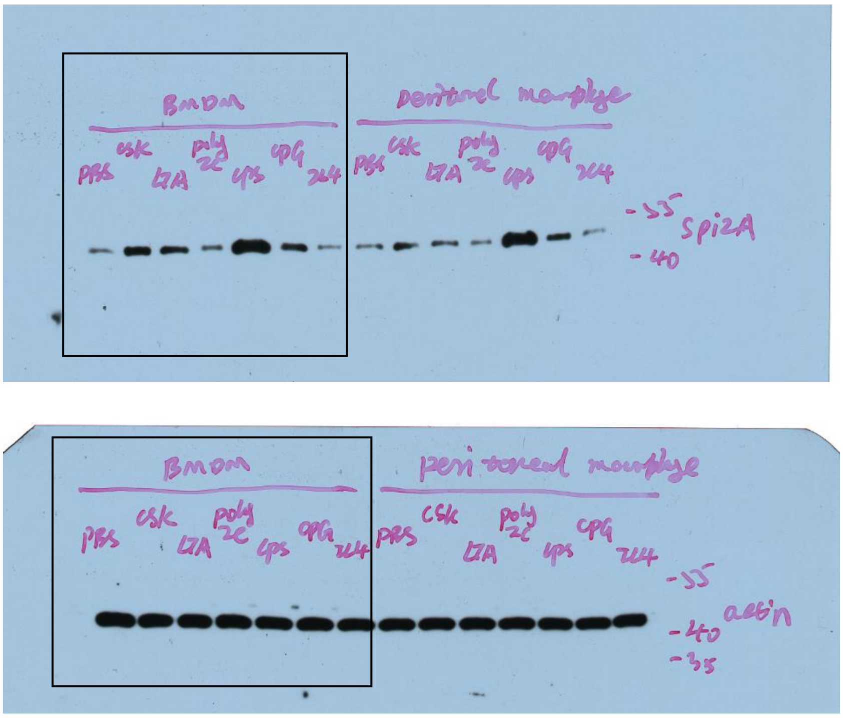

Figure 1c

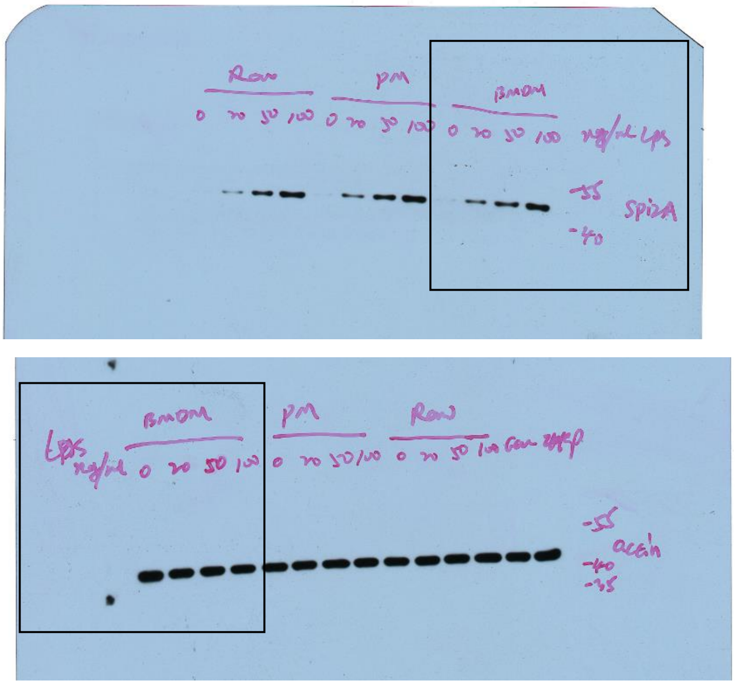

Figure 1e

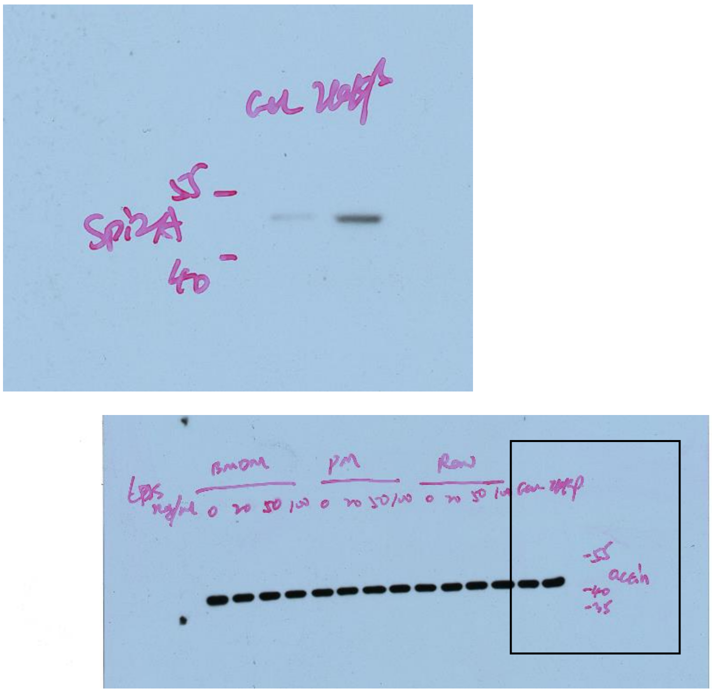

Figure 1g

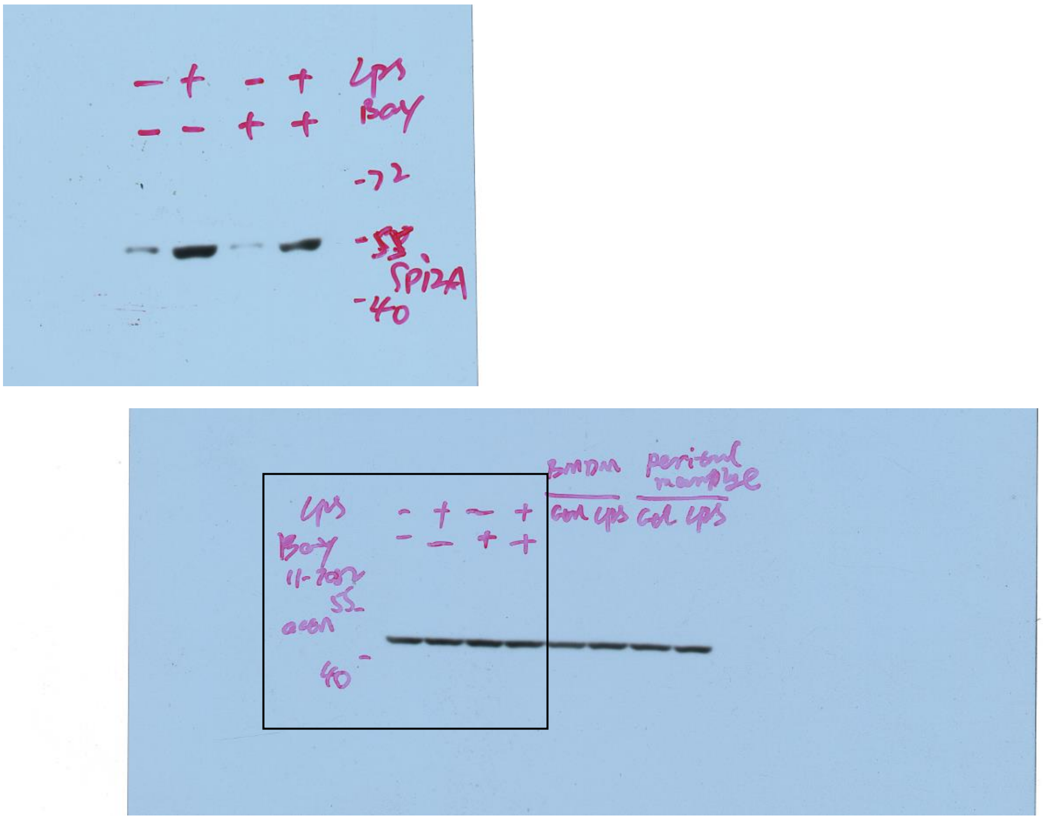

Figure 2a

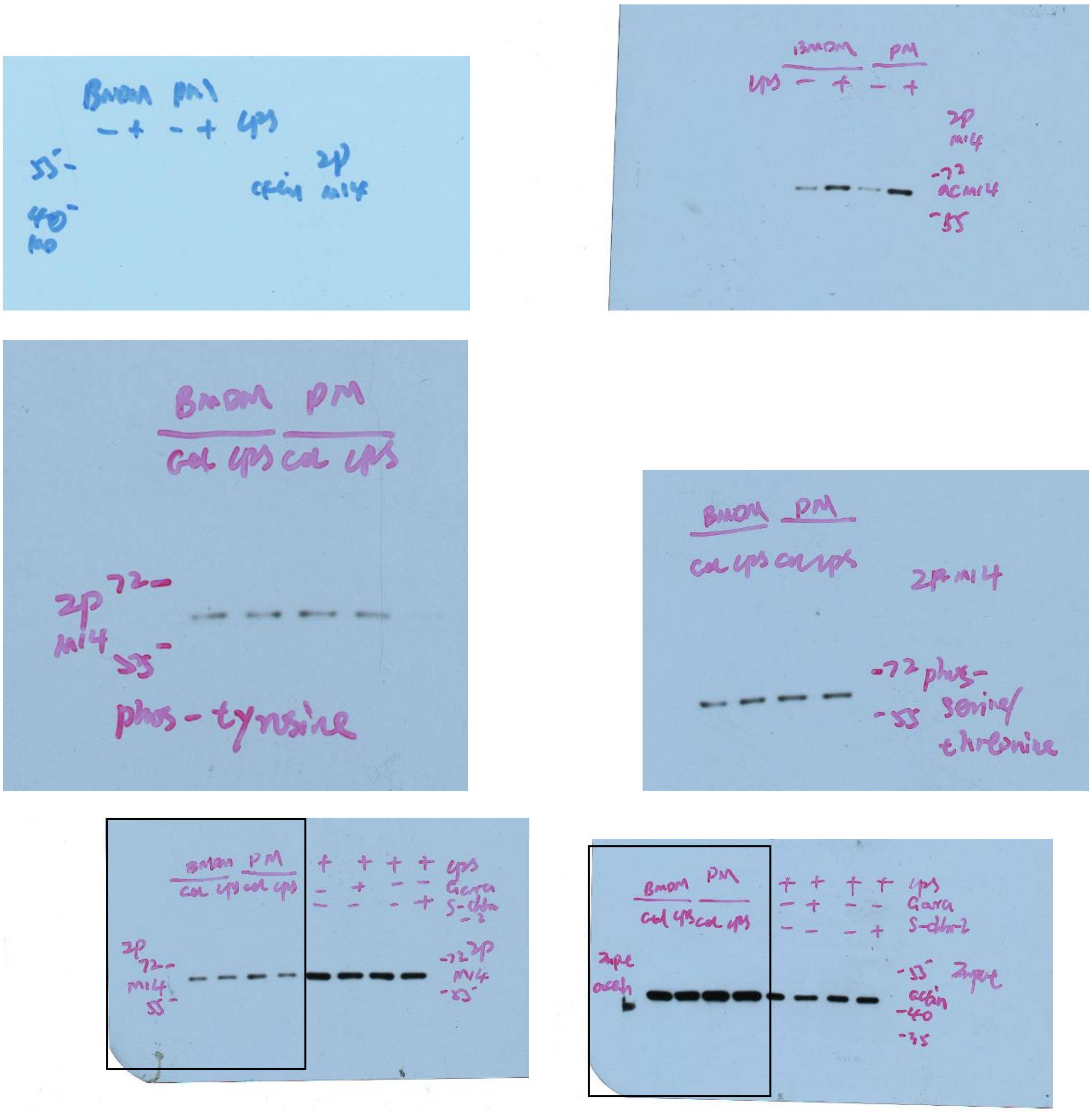

Figure 2c

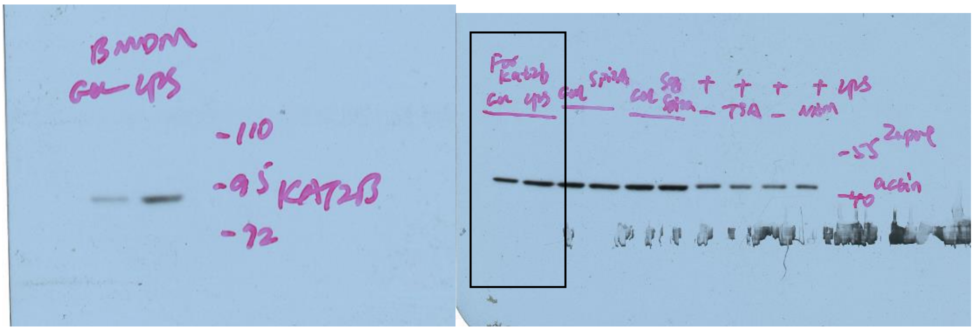

Figure 2d

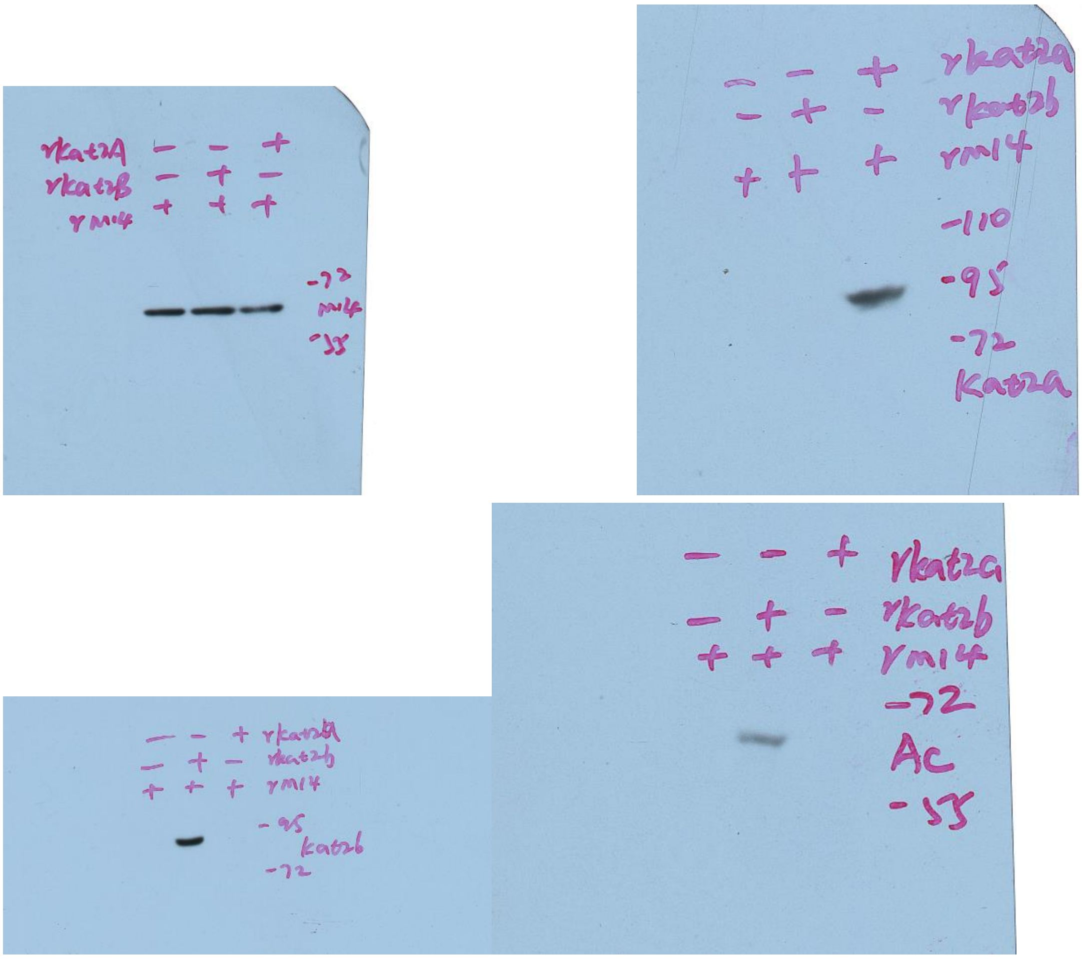

Figure 2e

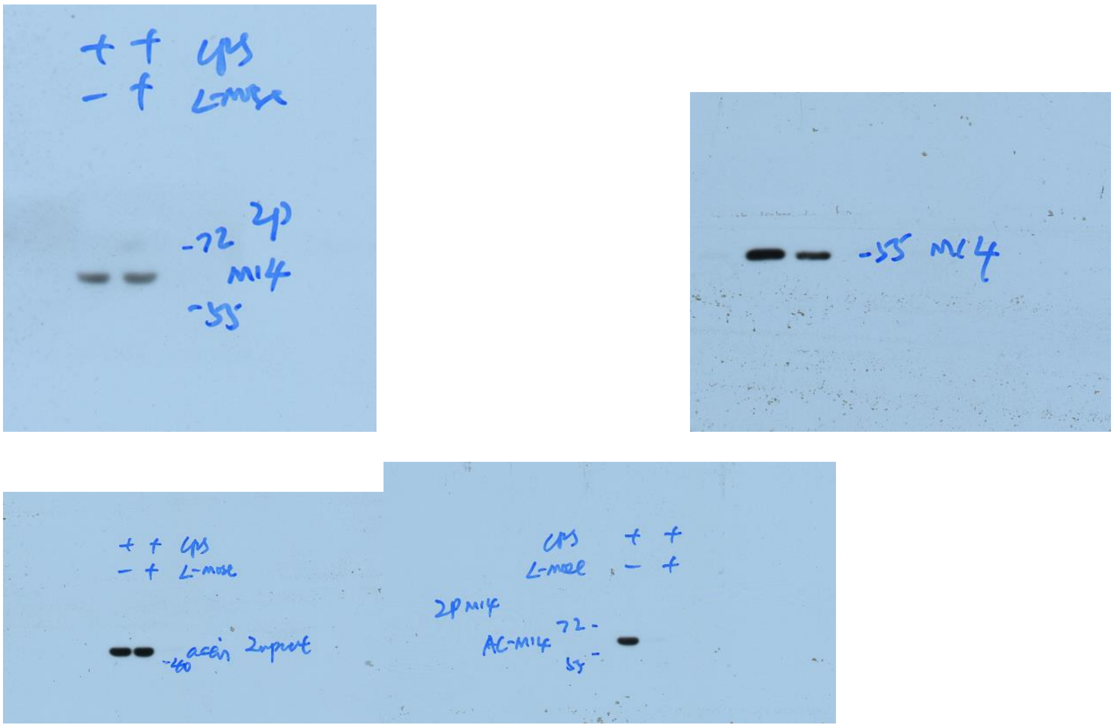

Figure 2g

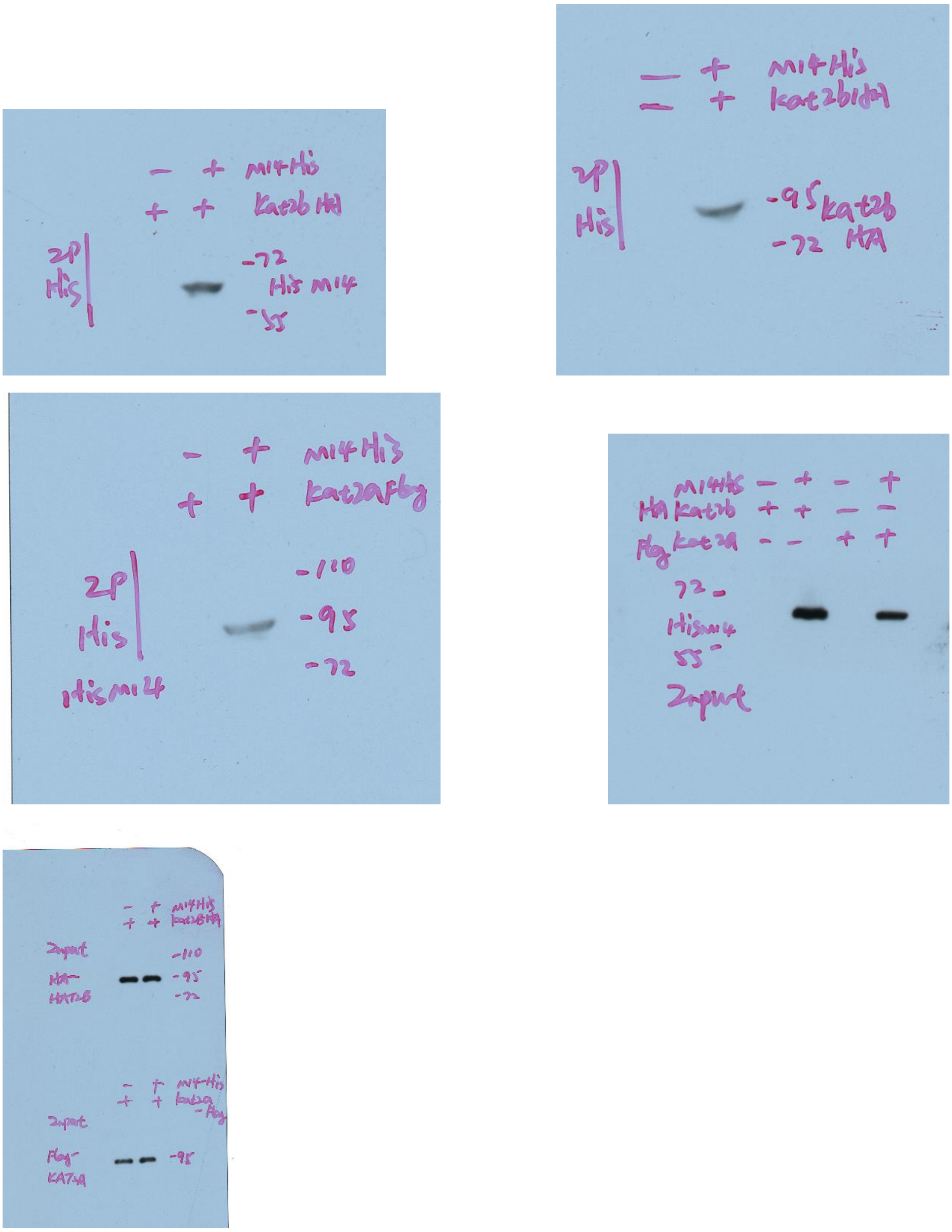

Figure 2h

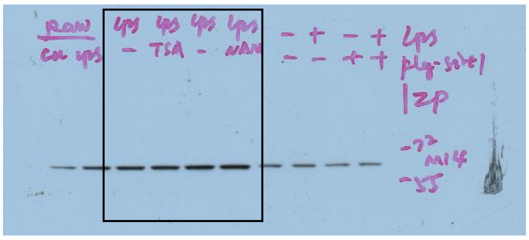

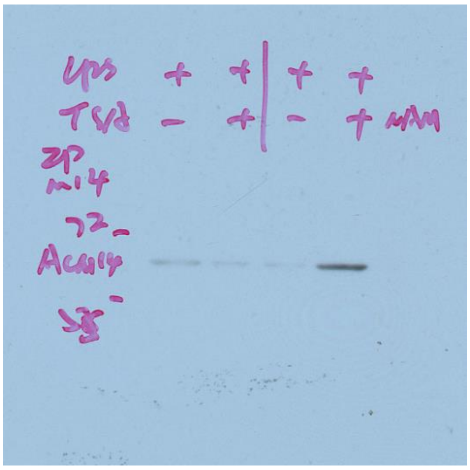

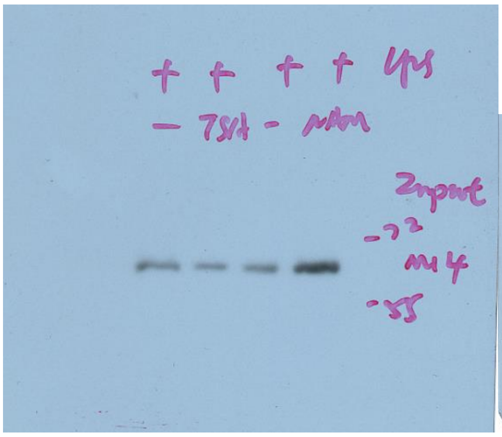

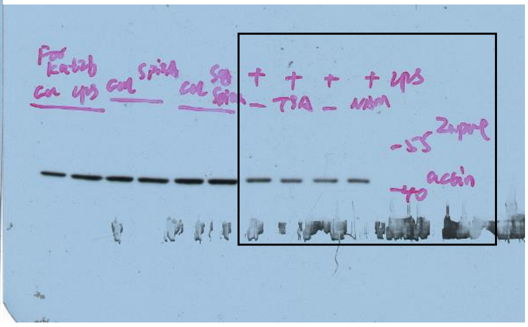

Figure 2i

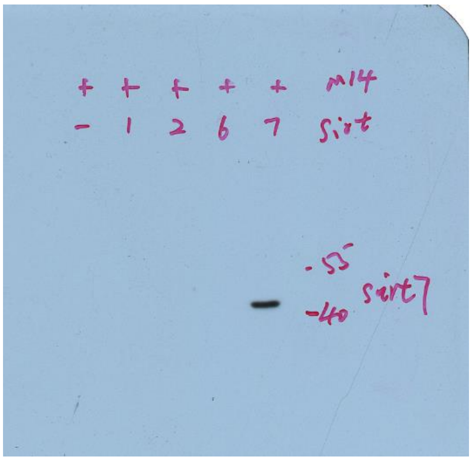

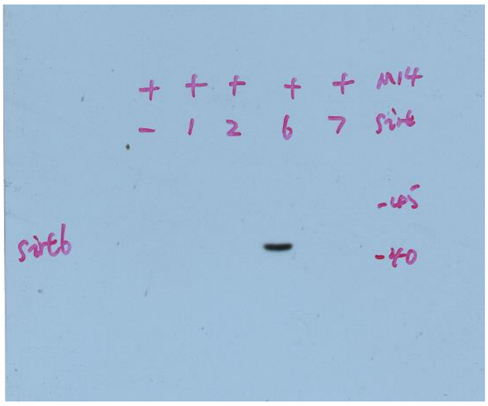

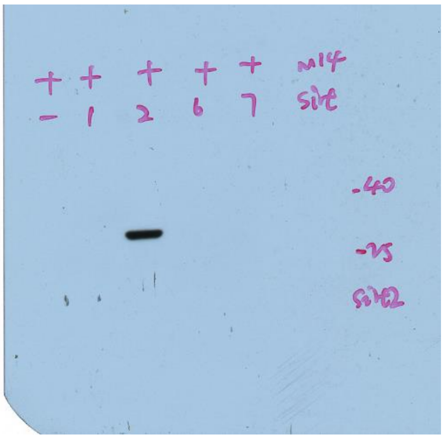

MIF
Sirt
-40
-35
Sirt2

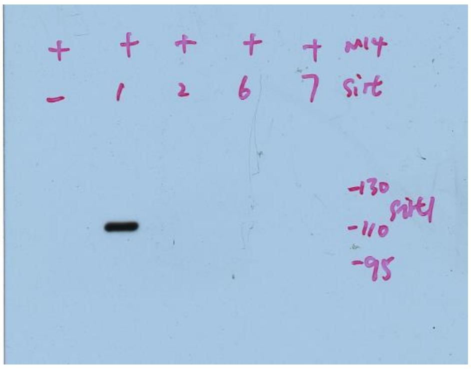

MIF
Sirt
-130
-110
Sirt1
-95

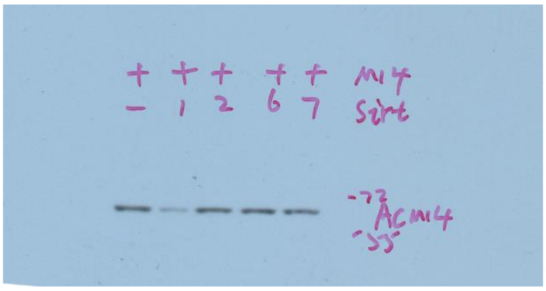

MIF
Sirt
-72
AcMIF
-55

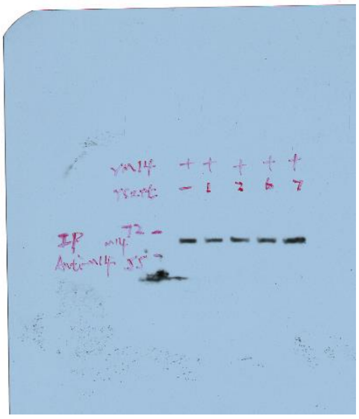

Figure 2j

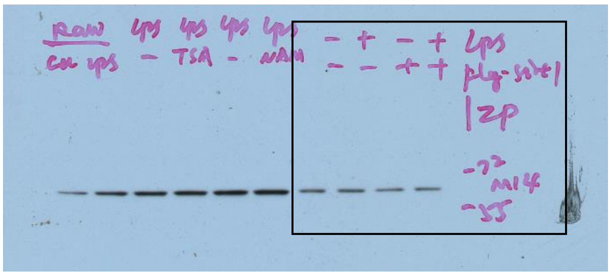

Raw
TSA
NAM
Lps
flag-sirt1
IP
-72
MIF
-55

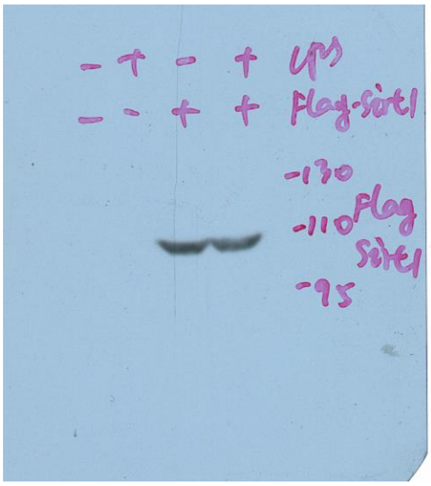

Lps
Flag-Sirt1
-130
-110
Flag
Sirt1
-95

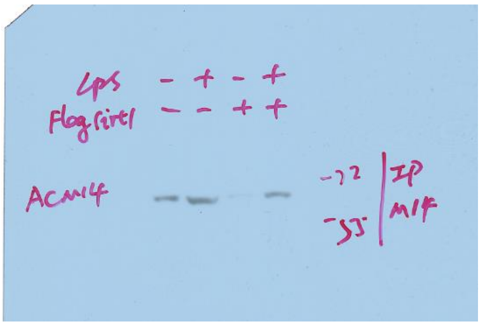

Lps
Flag sirt1
AcMIF
-72
-55
IP
MIF

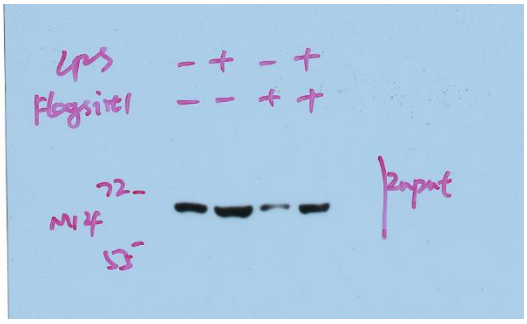

Lps
Flagsirt1
72
MIF
55
Input

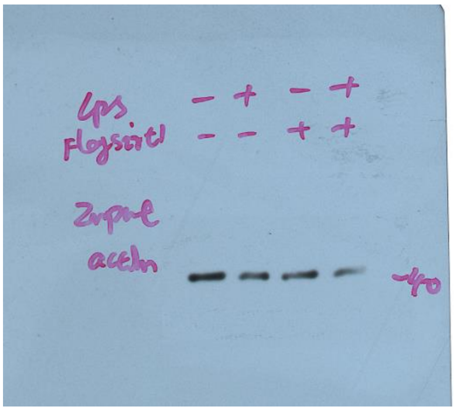

Figure 3f

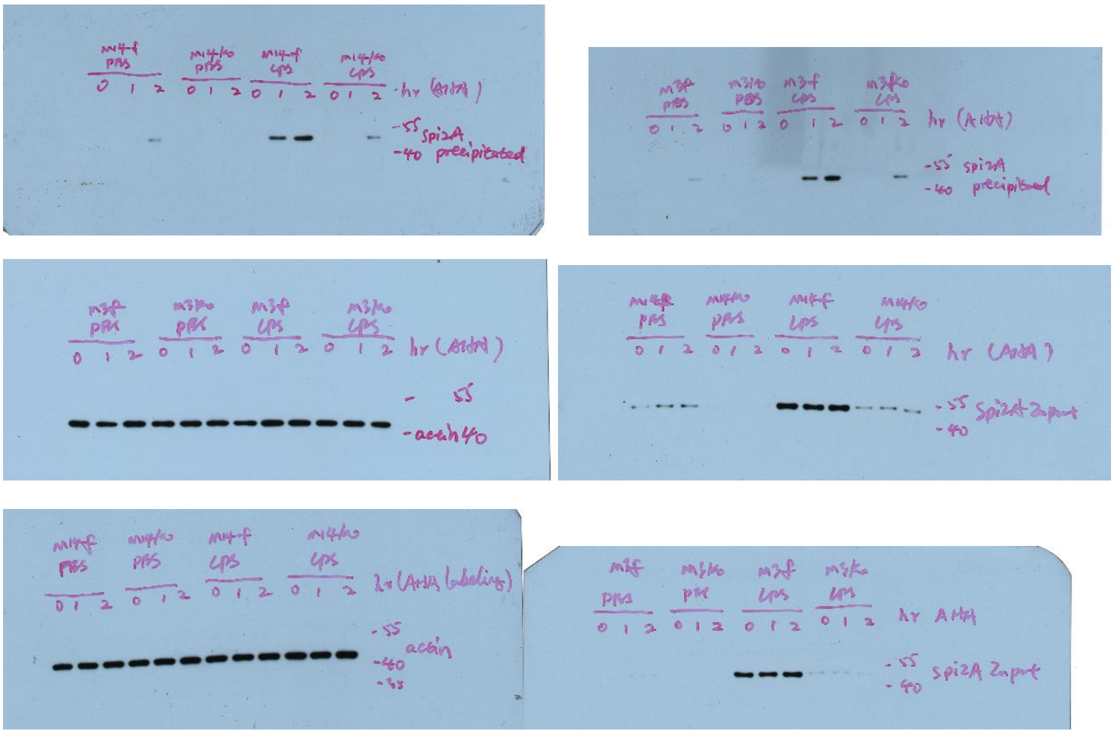

Figure 4c

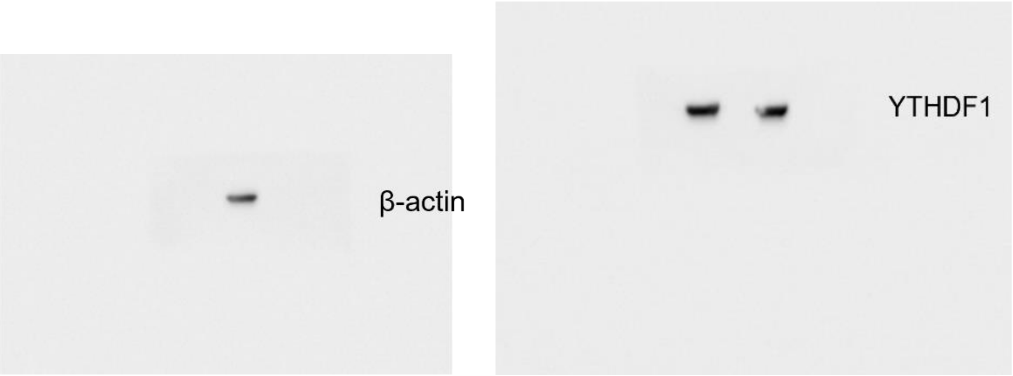

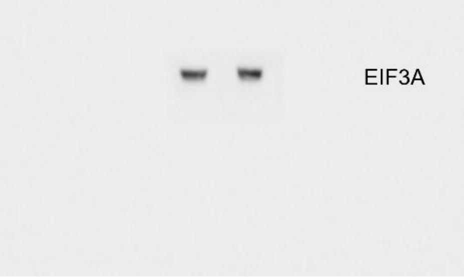

Figure 4d

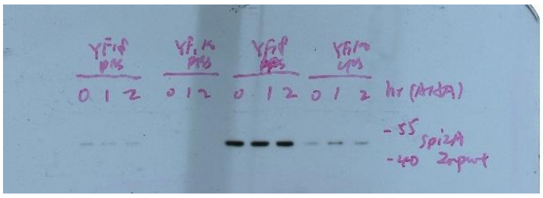

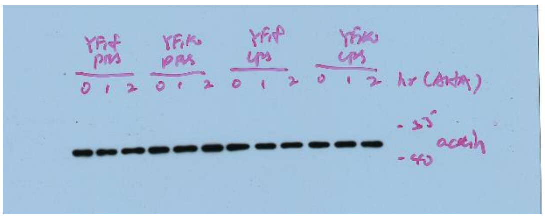

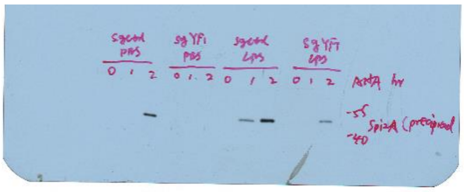

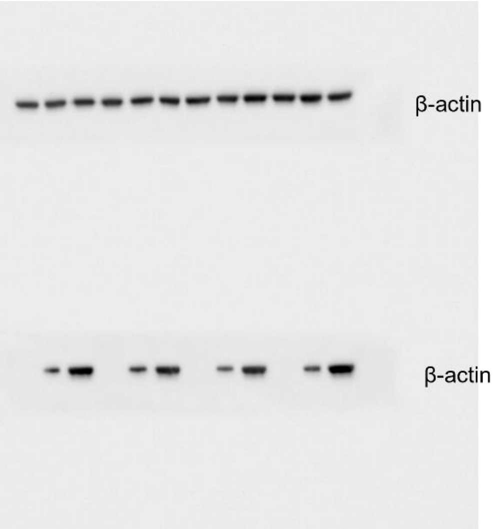

Figure 6b

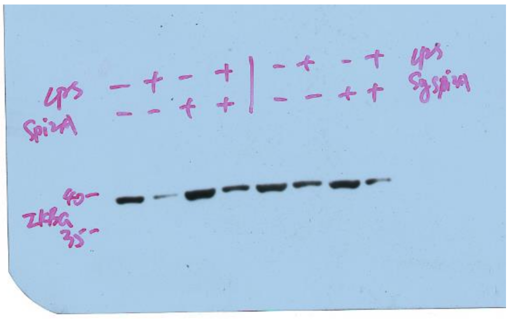

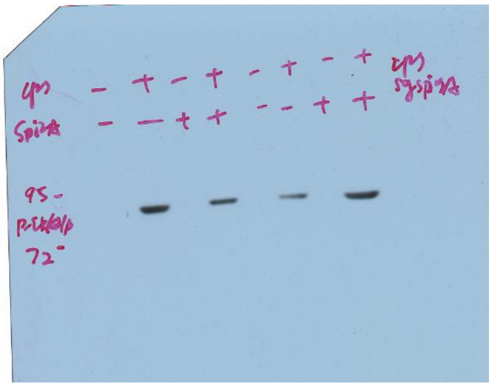

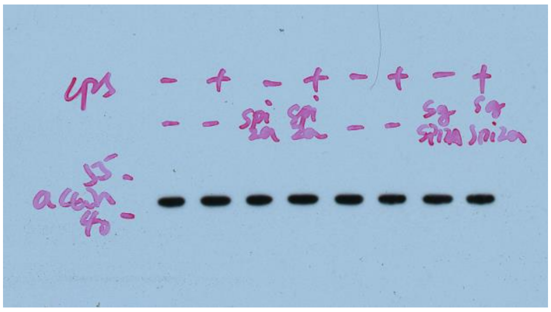
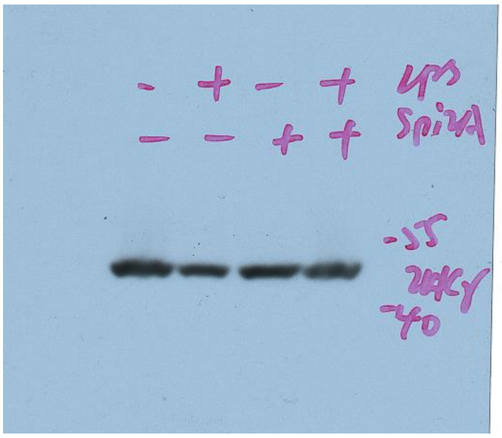

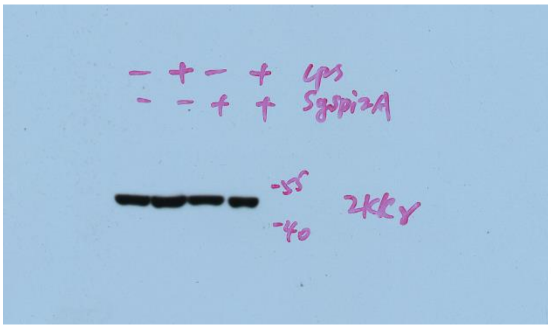

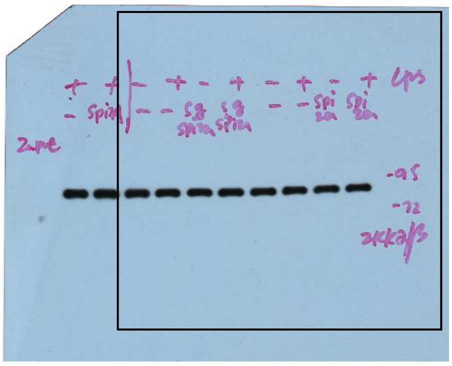
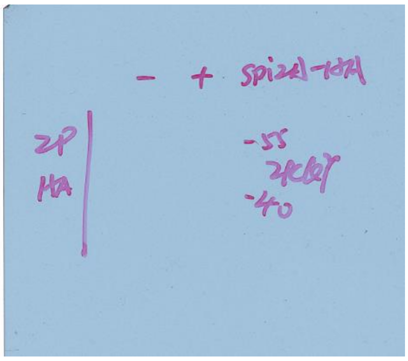

Figure 6c

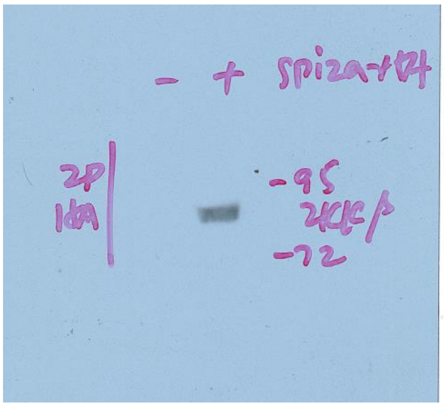

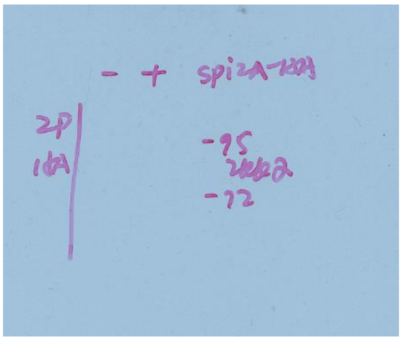

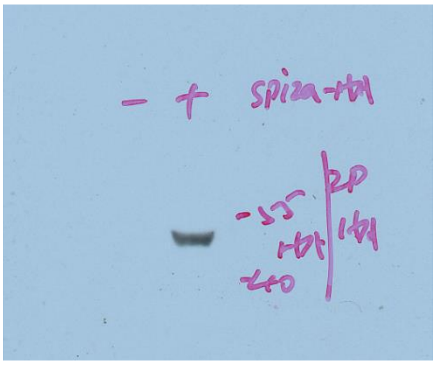

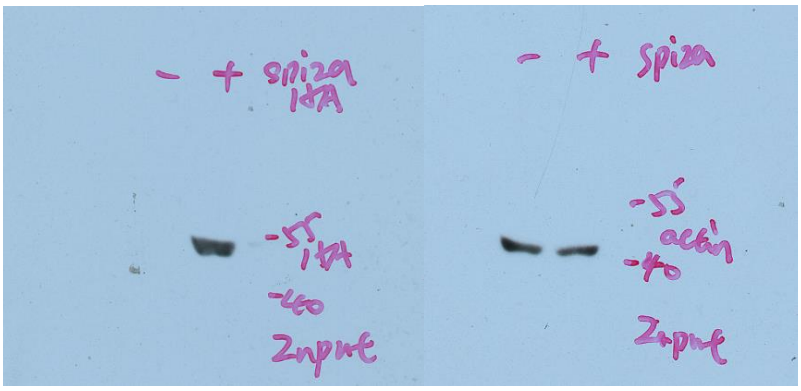

Figure 6d

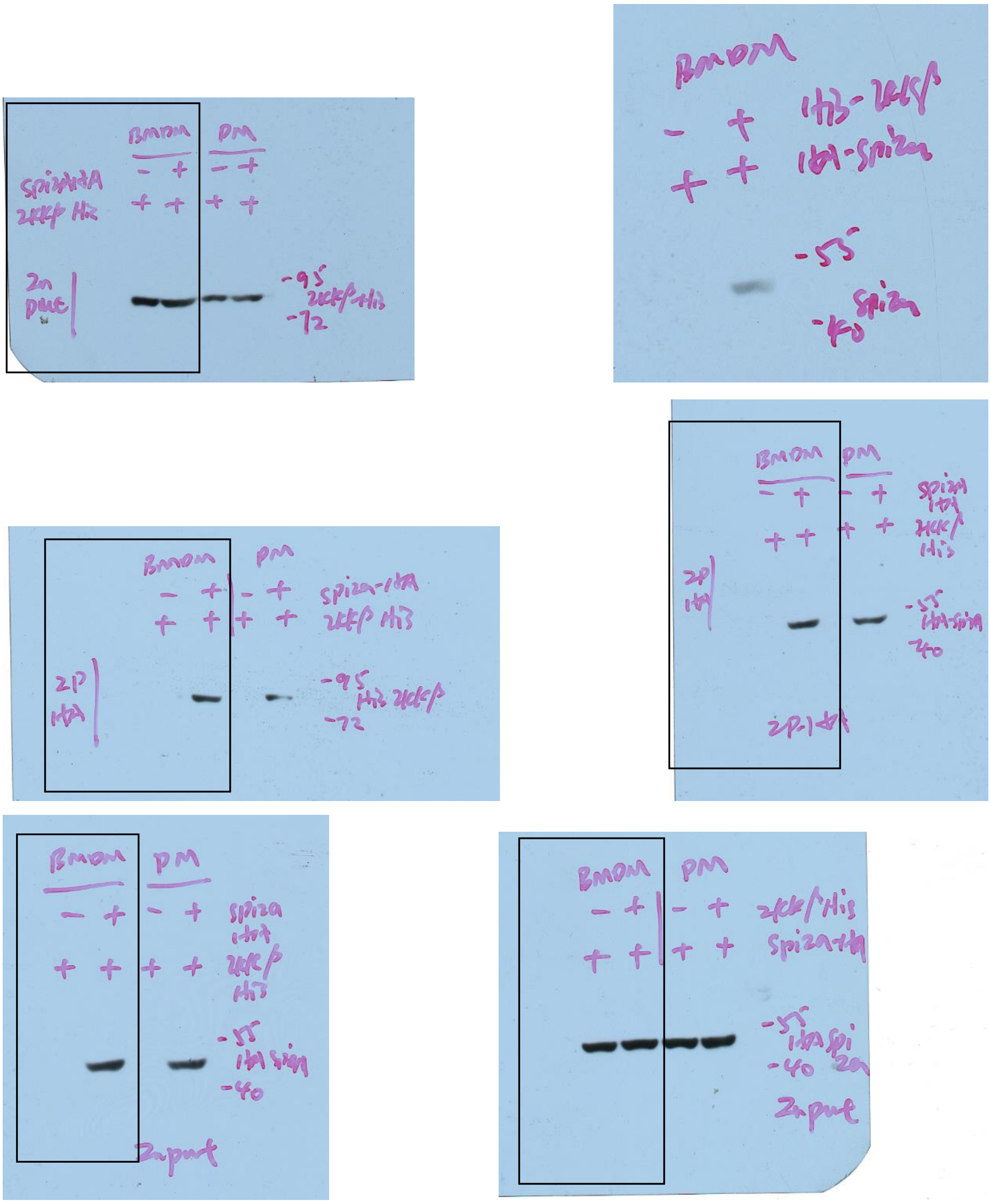

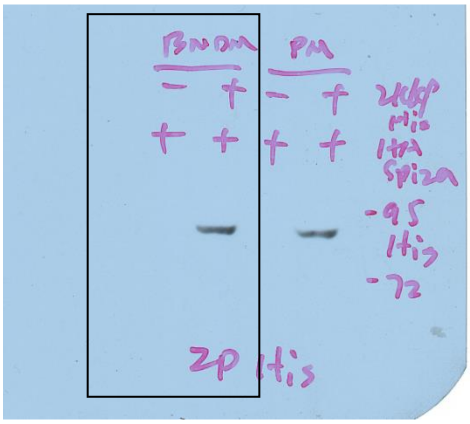

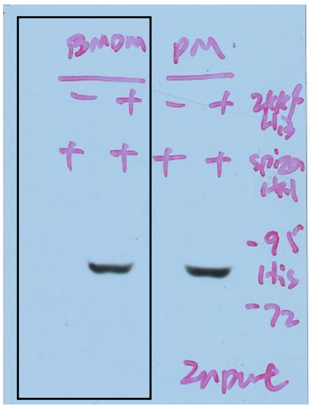

Figure 6e

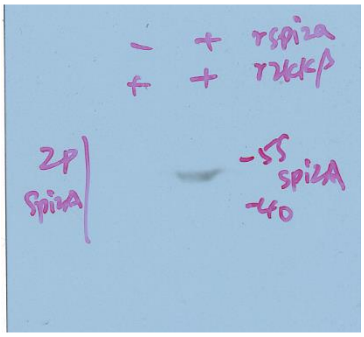

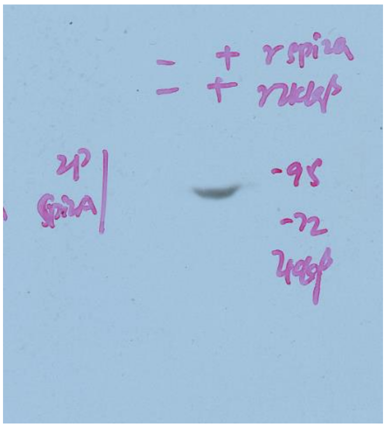

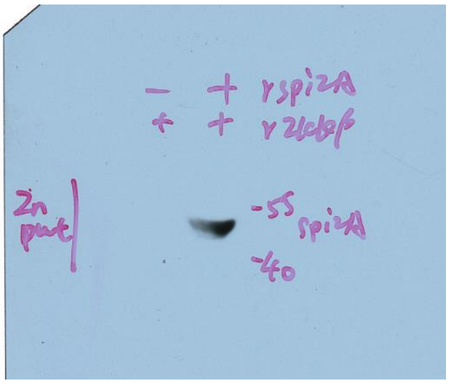

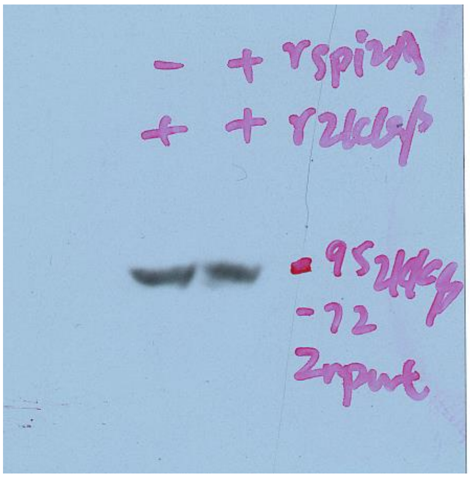

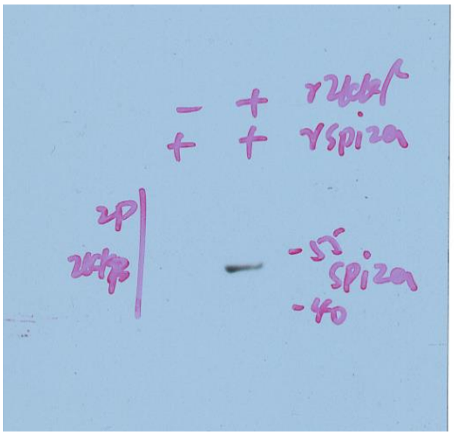

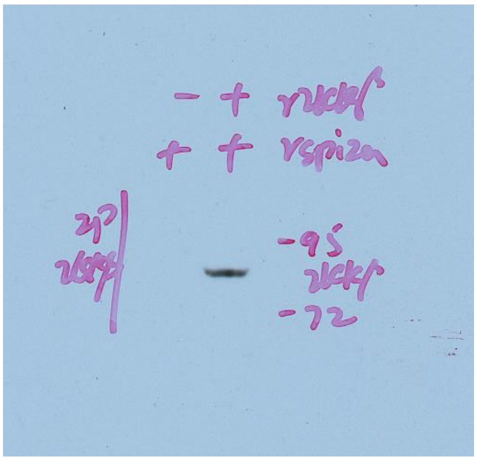

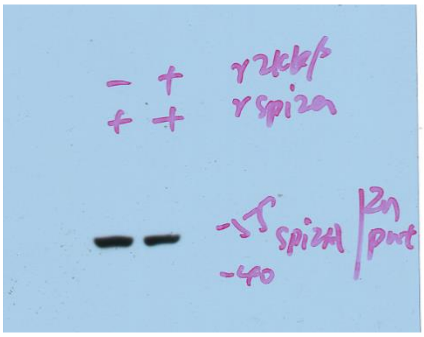

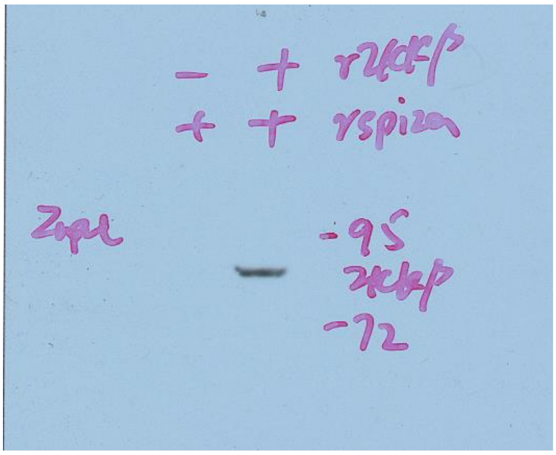

Figure 6f

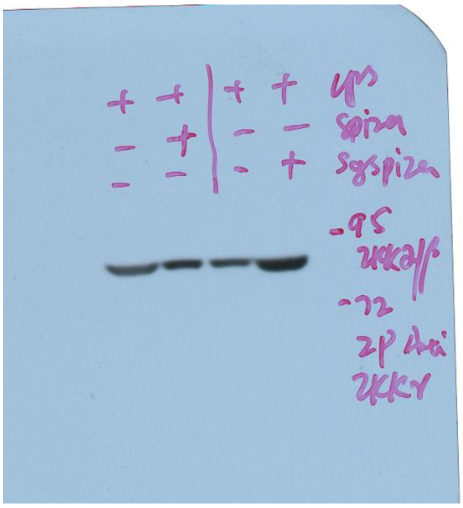

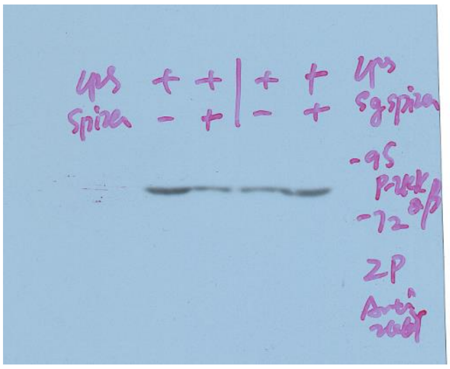

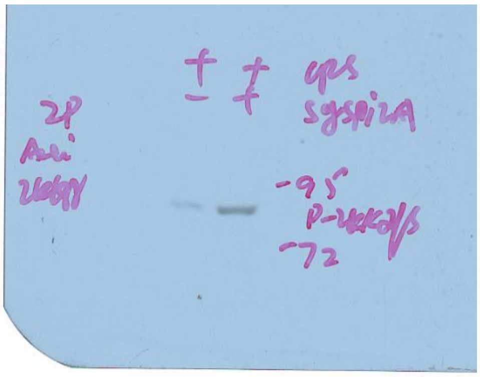

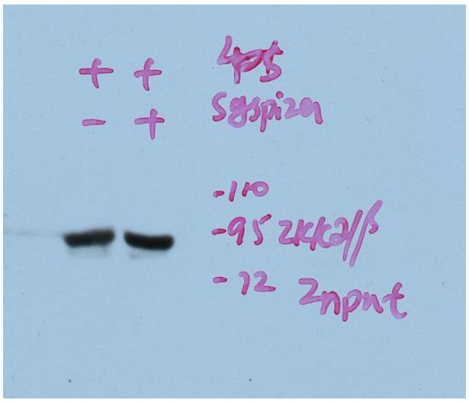

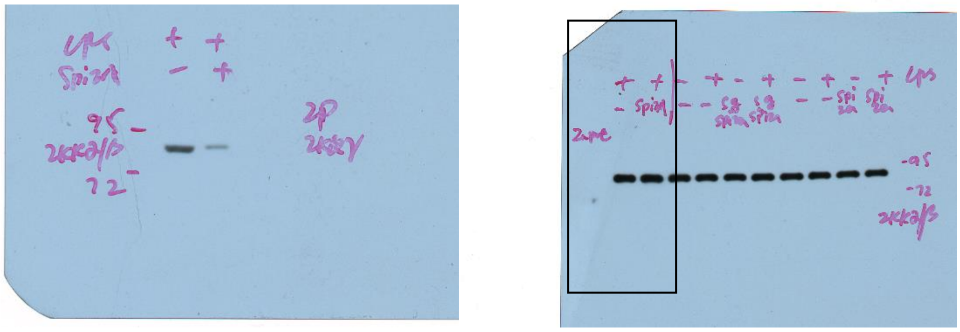

Supplementary figure 1a

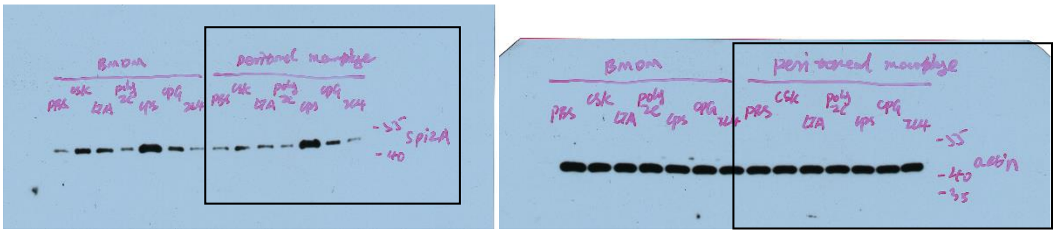

Supplementary figure 1b

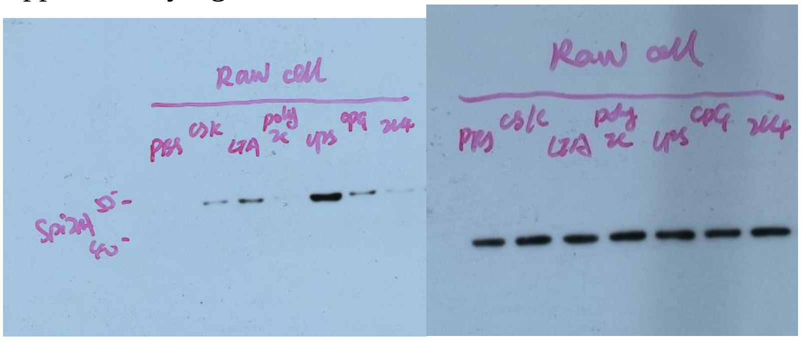

Supplementary figure 1d

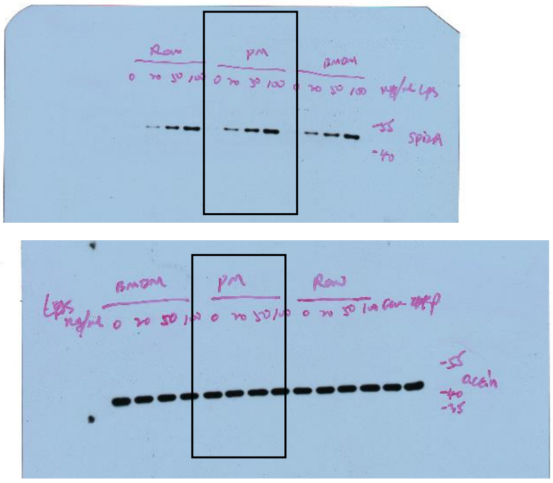

Supplementary figure 1e

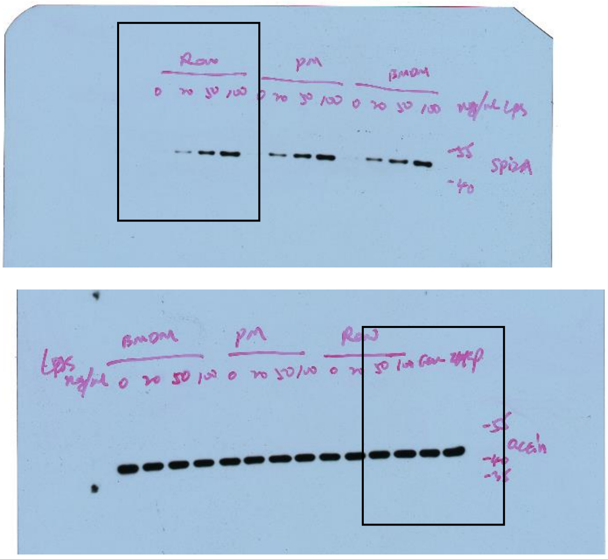

Supplementary figure 1f

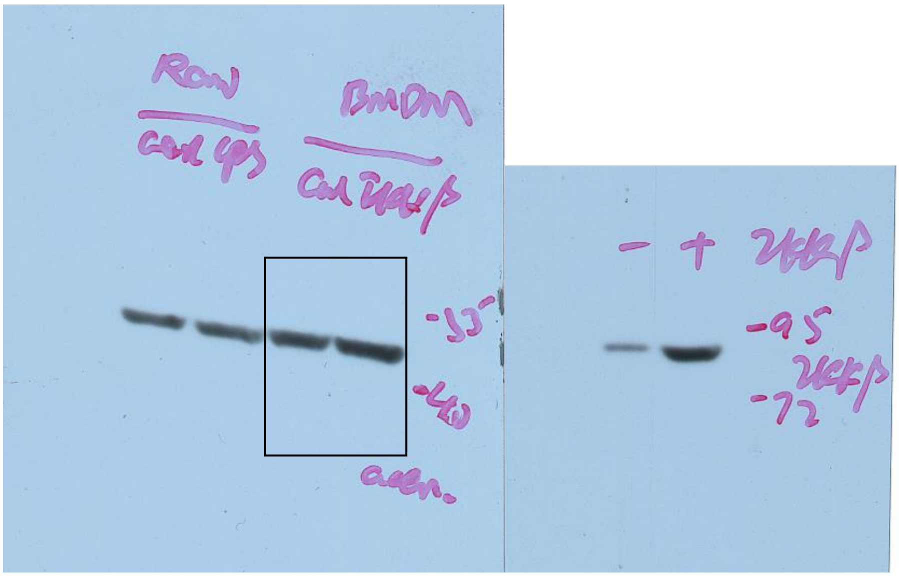

Supplementary figure 2c

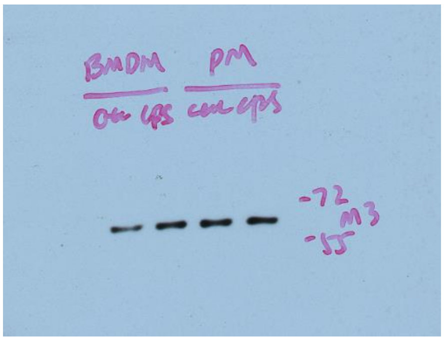

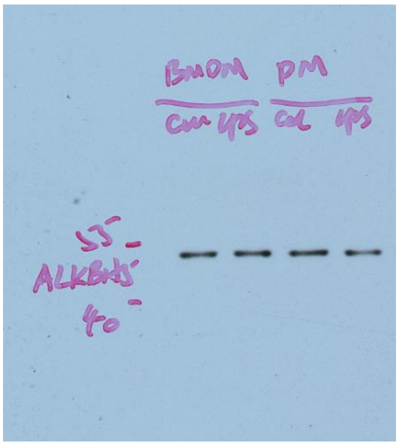

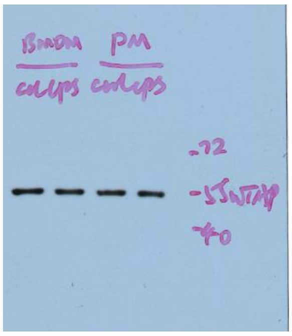

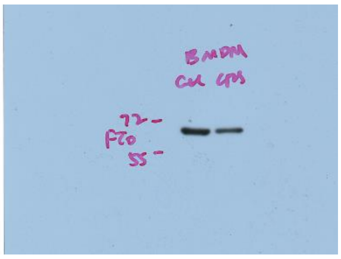

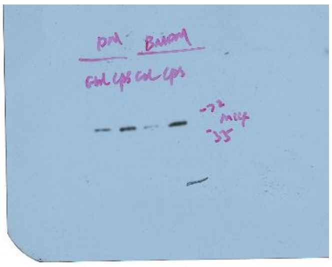

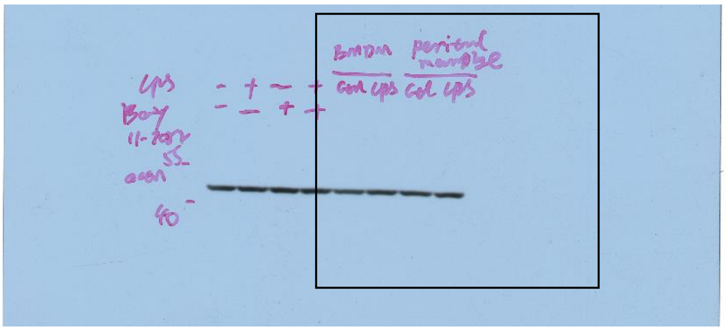

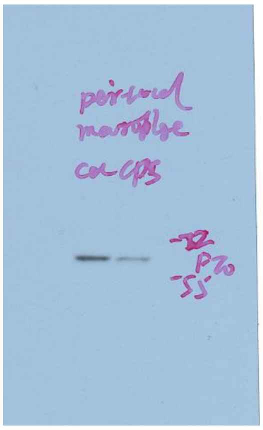

Supplementary figure 2d

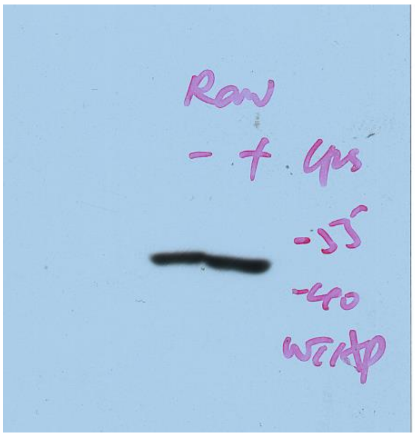

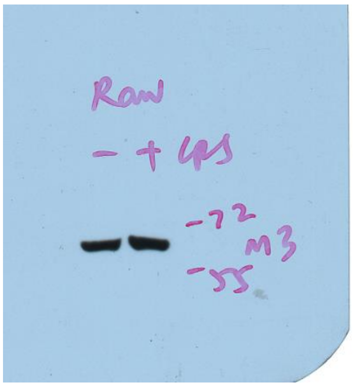

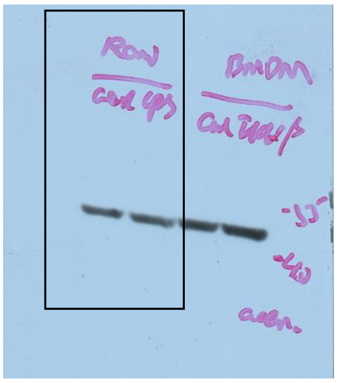

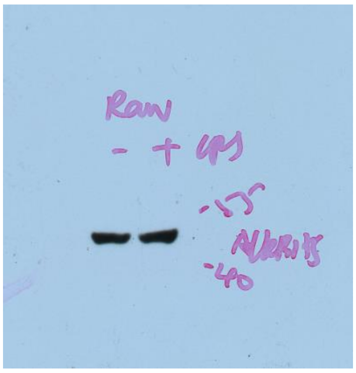

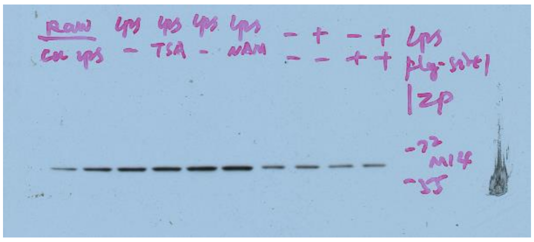

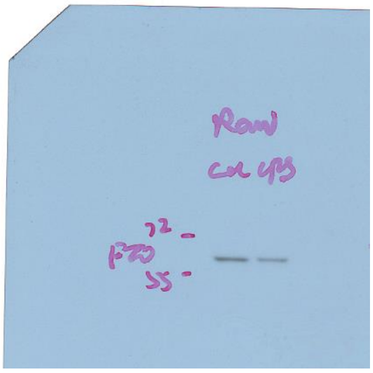

Supplementary figure 2e

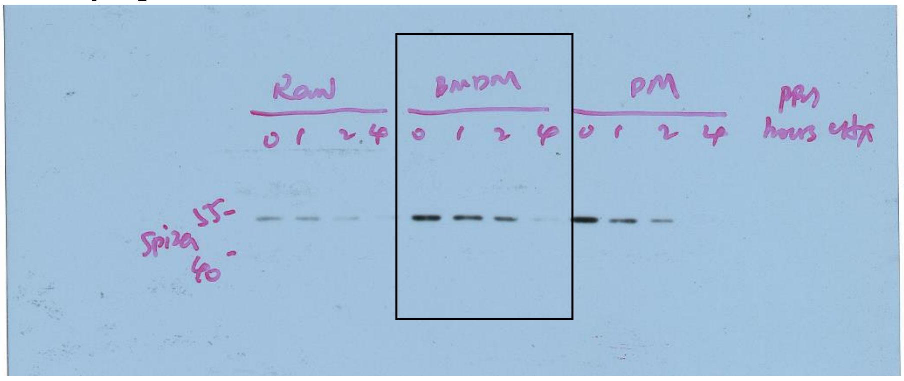

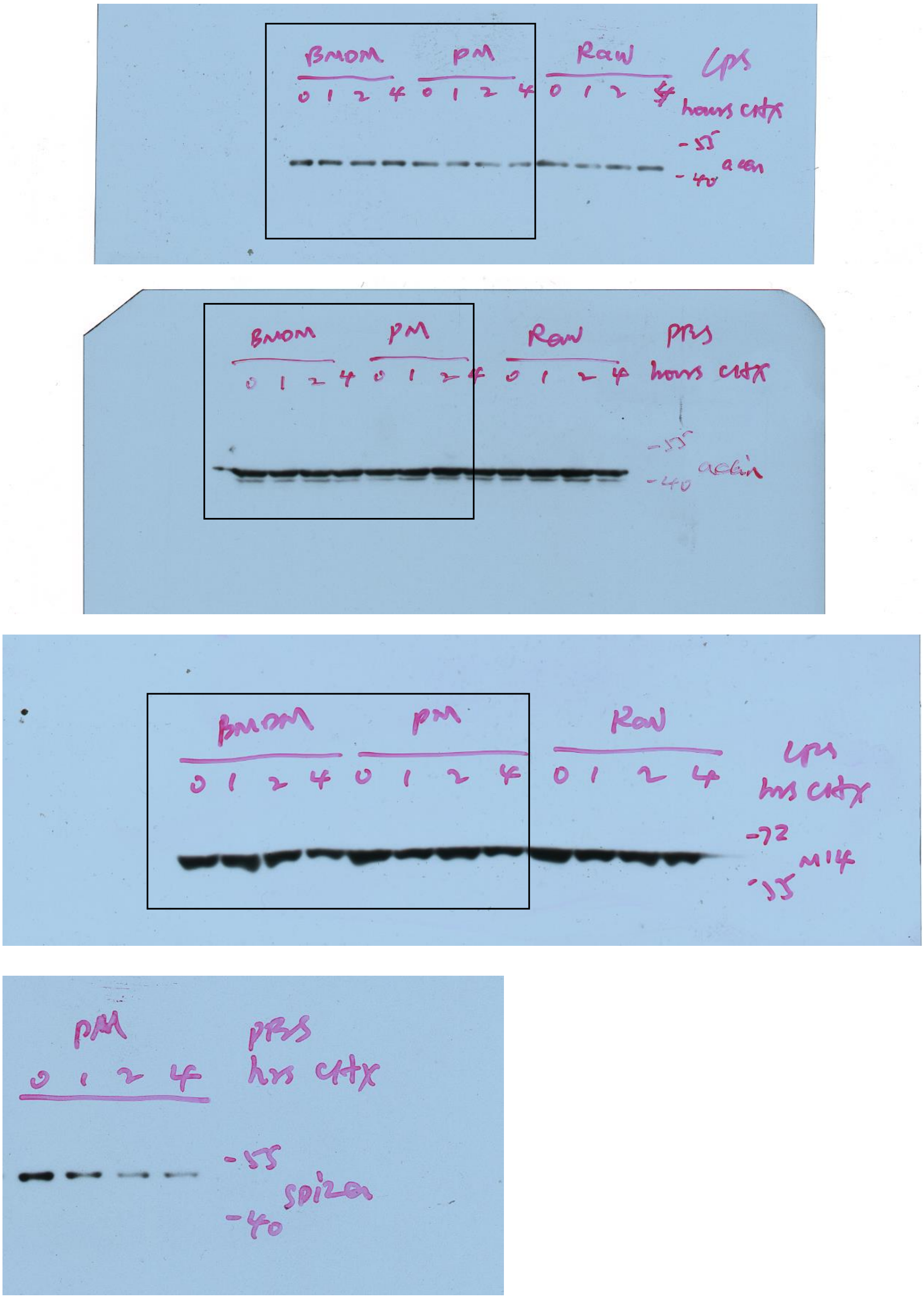

Supplementary figure 2f

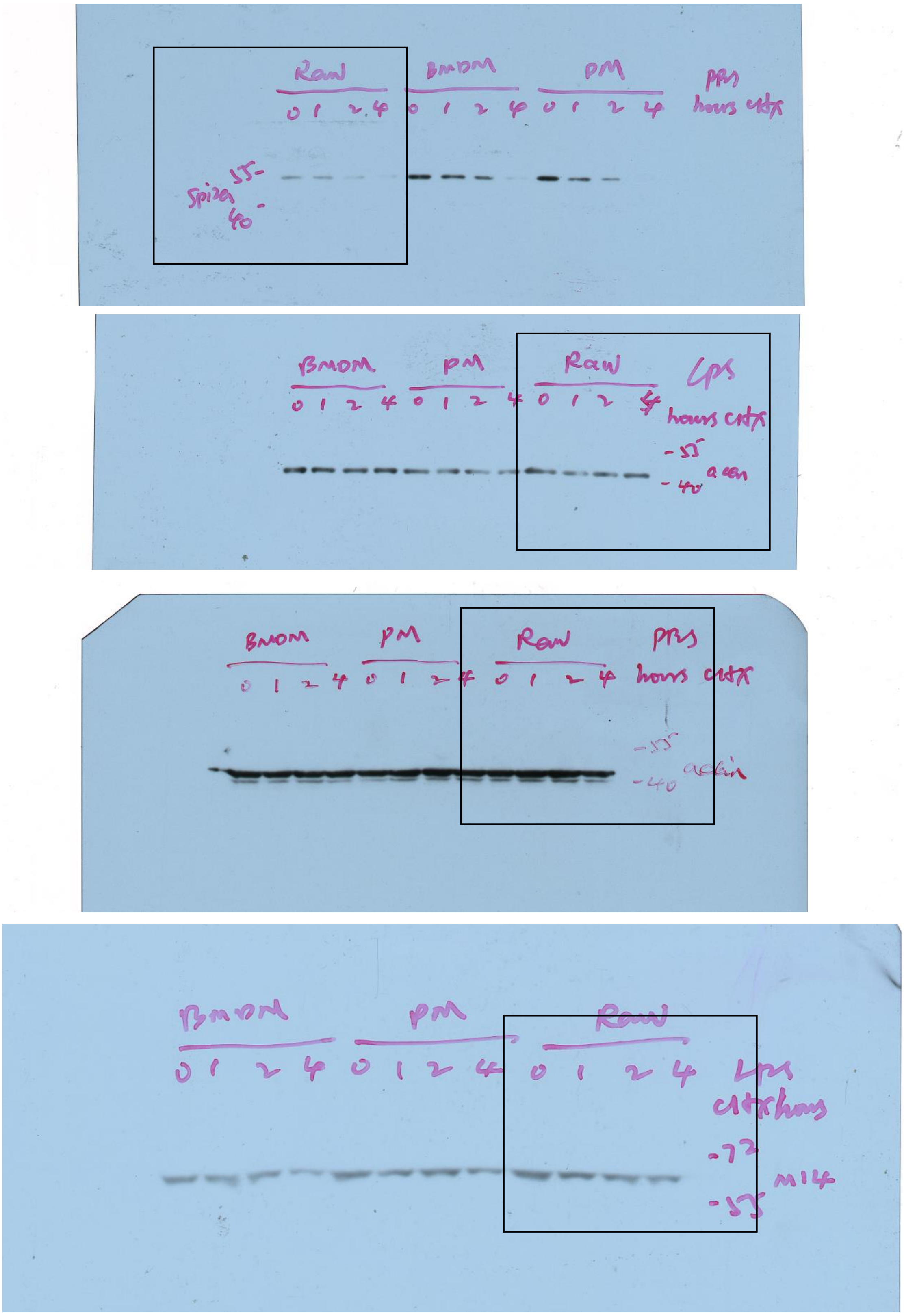

Supplementary figure 2g

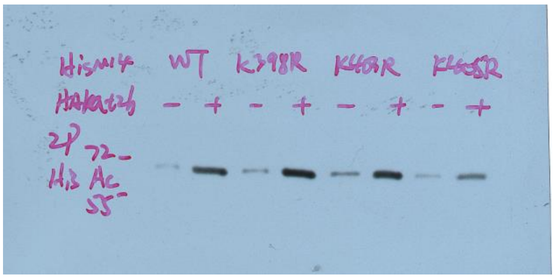

Hismi4
WT K398R K403R K405R
HAkat2b - + - + - + - +
72
H3 Ac
55

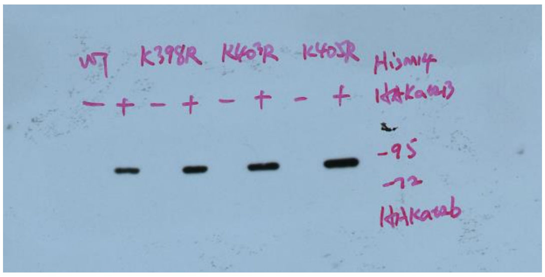

WT K398R K403R K405R
Hismi4
- + - + - + - +
HAkat2b
-95
-72
HAkat2b

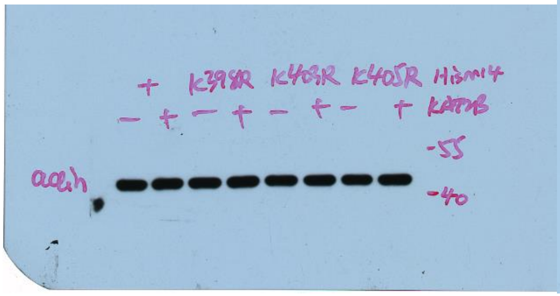

+ K398R K403R K405R Hismi4
- + - + - + - + KAT2B
-55
actin
-40

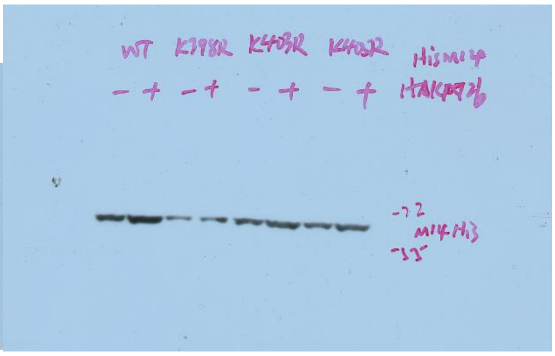

WT K398R K403R K405R
Hismi4
- + - + - + - +
HAKat2b
-72
mi4 His
-55

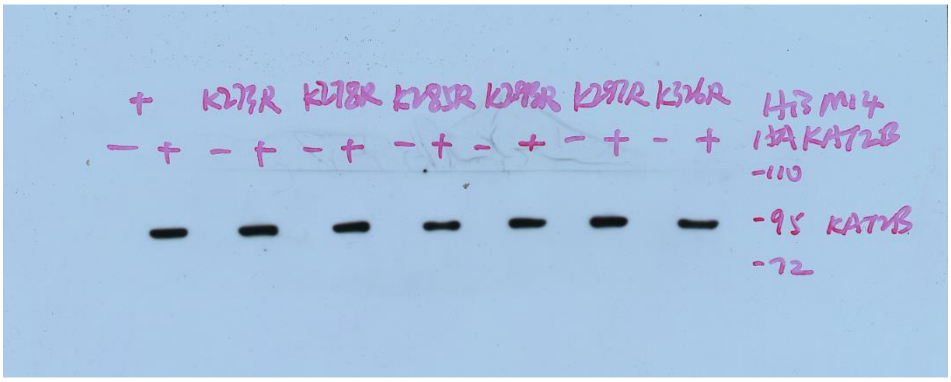

+ K273R K278R K285R K293R K297R K326R
His mi4
- + - + - + - + - + - + - +
HA KAT2B
-110
-95 KAT2B
-72

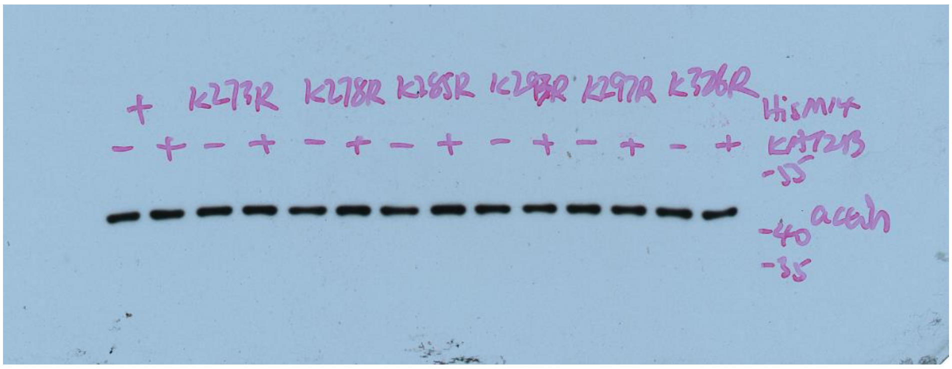

+ K273R K278R K285R K293R K297R K326R
His mi4
- + - + - + - + - + - + - +
KAT2B
-55
-40 actin
-35

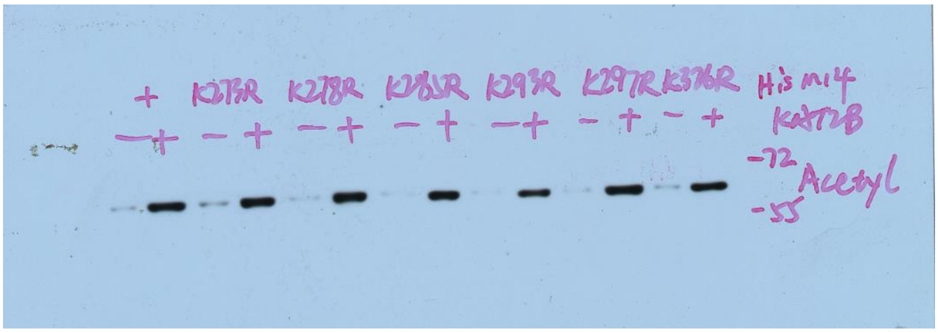

+ K273R K278R K285R K293R K297R K326R
His mi4
- + - + - + - + - + - + - +
KAT2B
-72
Acetyl
-55

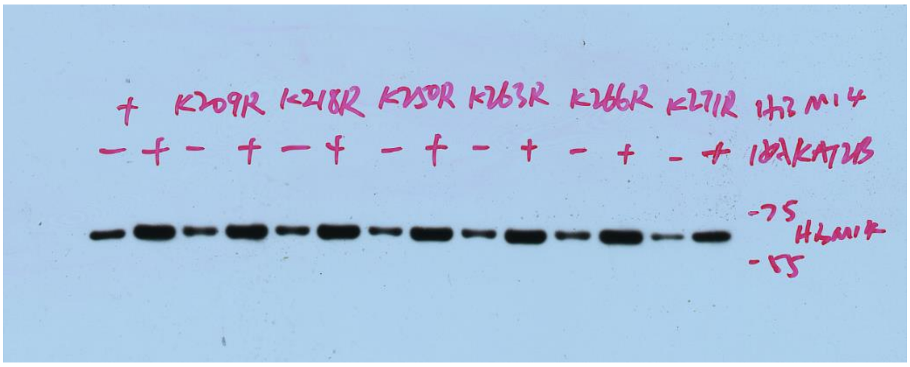
K209R K218R K263R K266R K271R
-75
-55

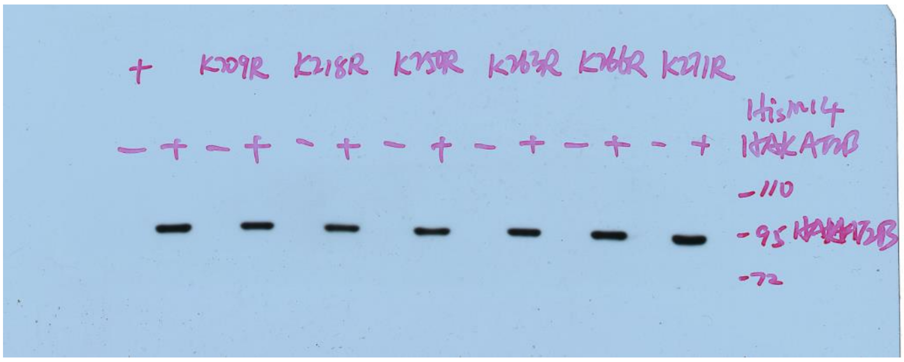
K209R K218R K263R K266R K271R
-110
-95
-72

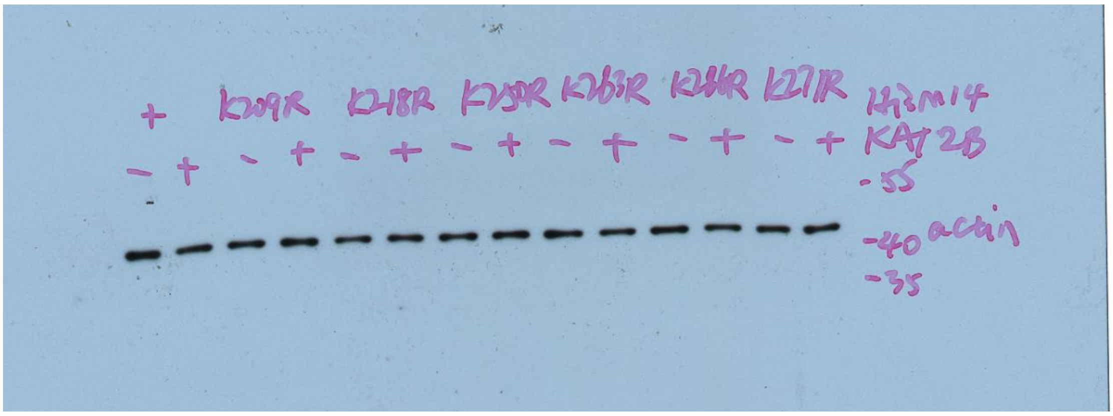
K209R K218R K263R K271R
KAT2B
-55
-40 actin
-35

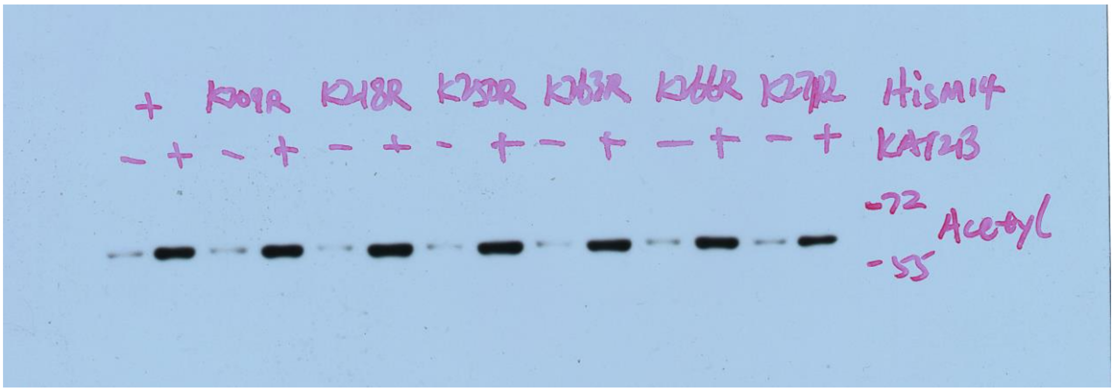
K218R
KAT2B
-72
Acetyl
-55

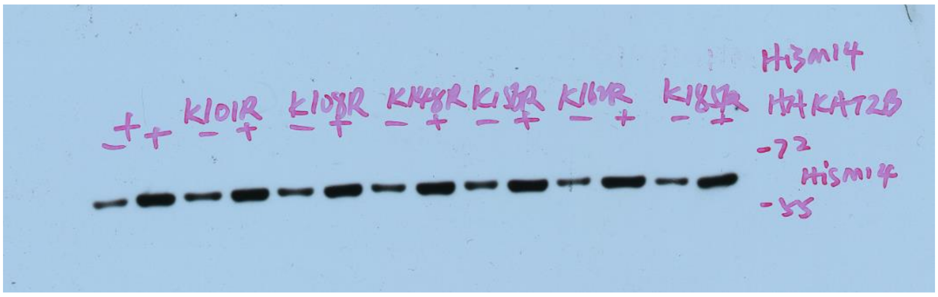
His m14
HAKAT2B
K101R K108R K148R K158R K160R K185R
-72
His m14
-55

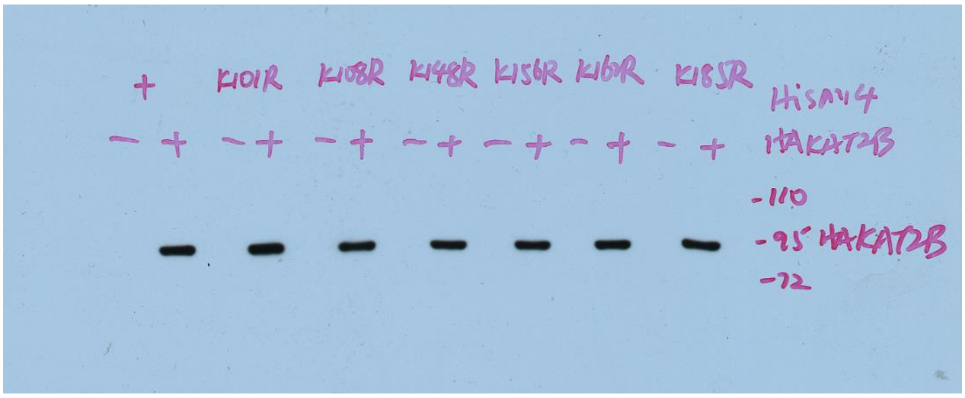
K101R K108R K148R K156R K160R K185R
His m14
HAKAT2B
-110
-95 HAKAT2B
-72

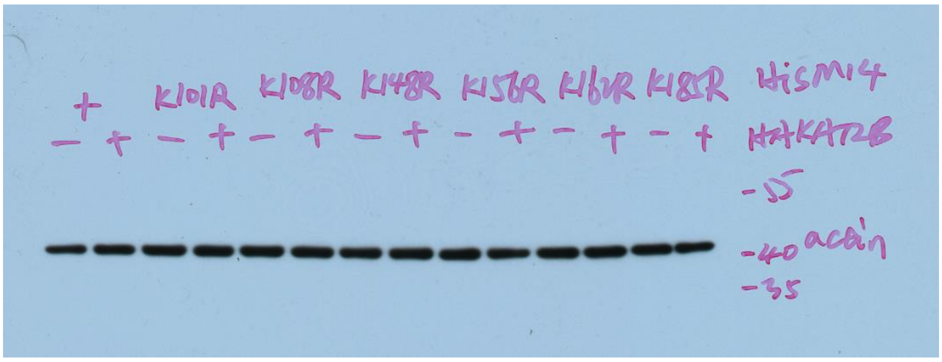
K101R K108R K148R K156R K160R K185R
His m14
HAKAT2B
-55
-40 actin
-35

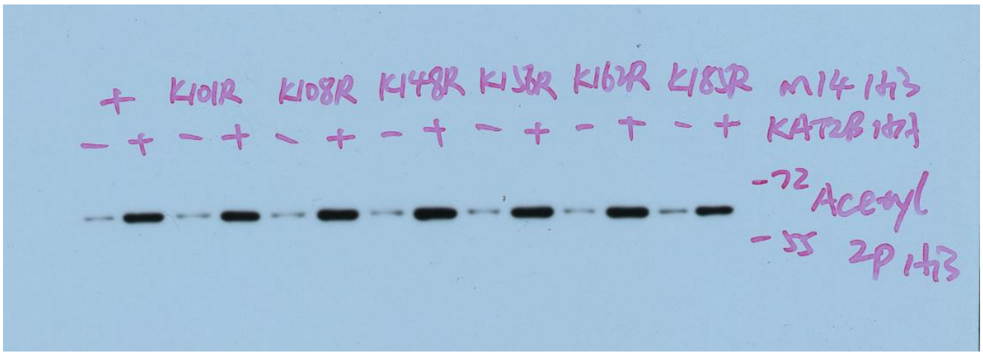
K101R K108R K148R K158R K160R K185R
m14 His
KAT2B HA
-72
Acetyl
-55
IP His

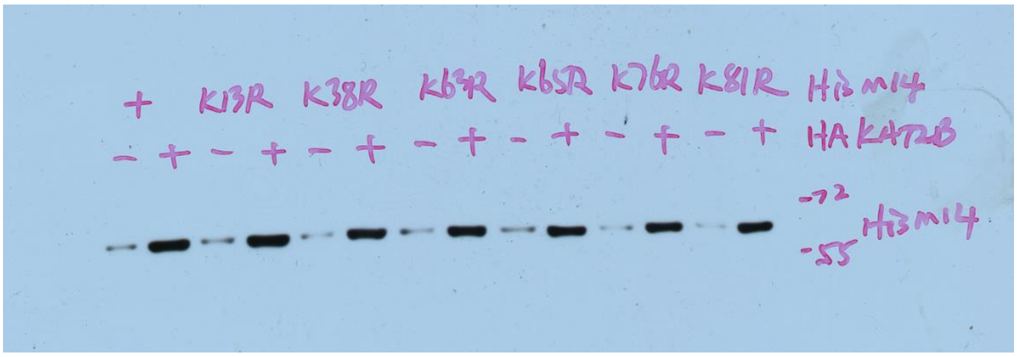
K13R K38R K63R K65R K76R K81R
His m14
HA KAT2B
-72
His m14
-55

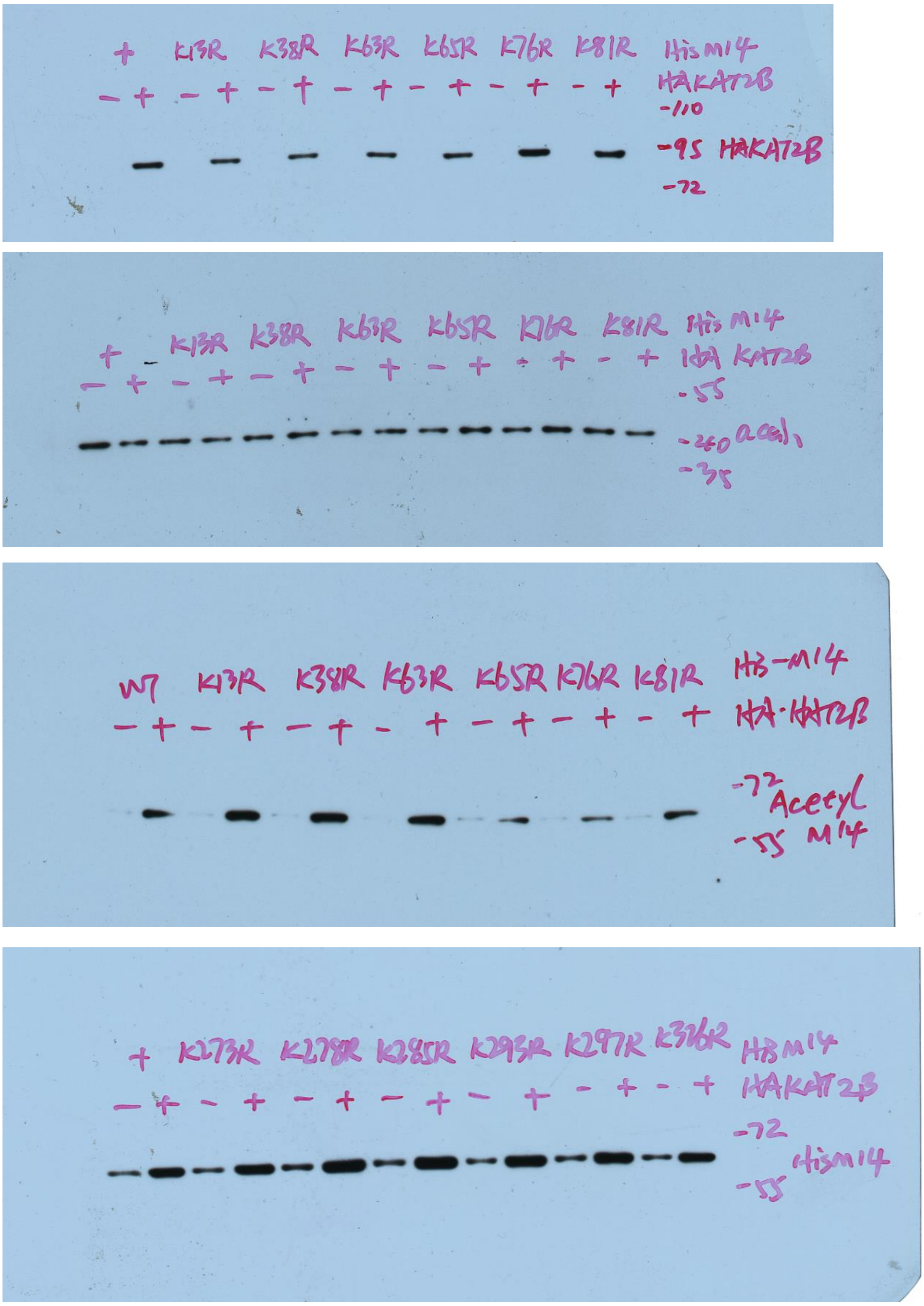

Supplementary figure 3a

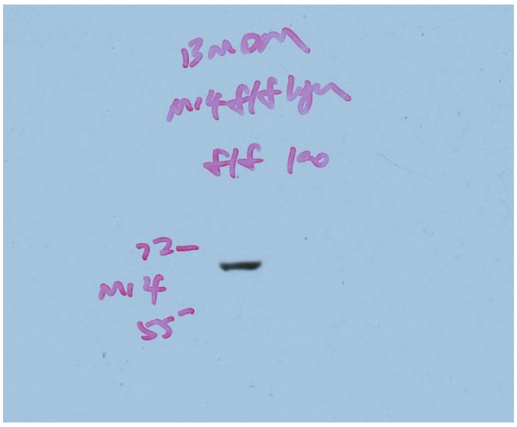

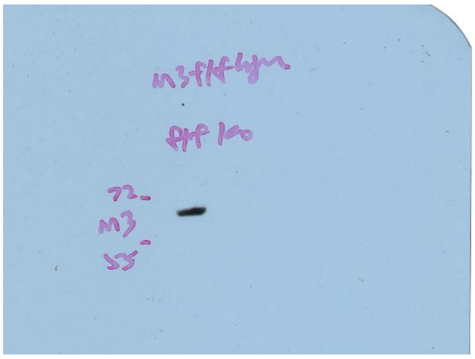

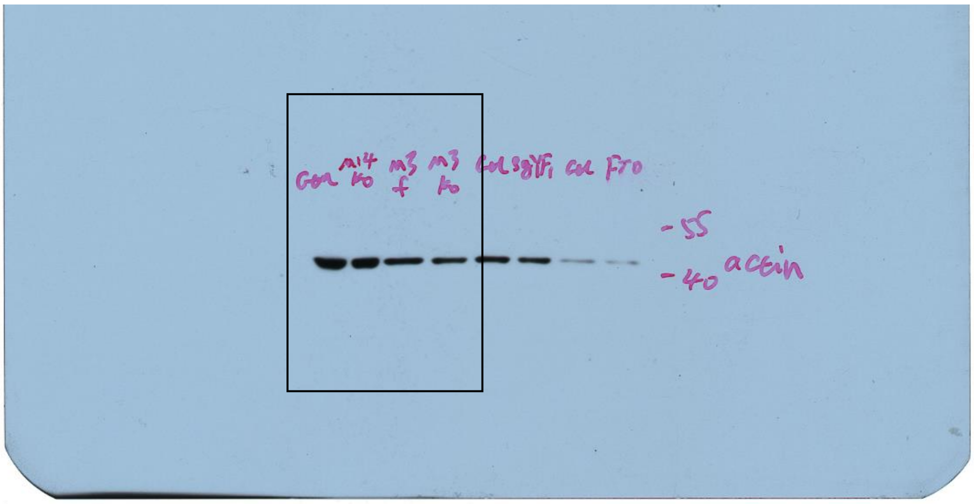

Supplementary figure 3c

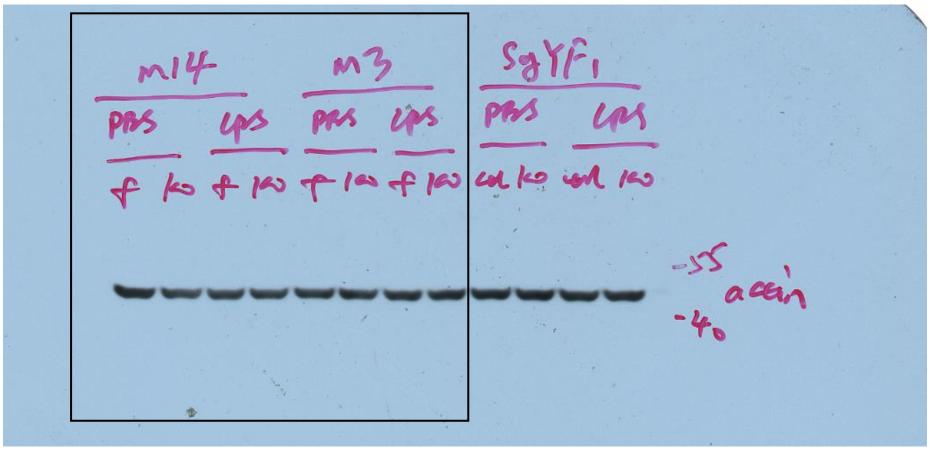

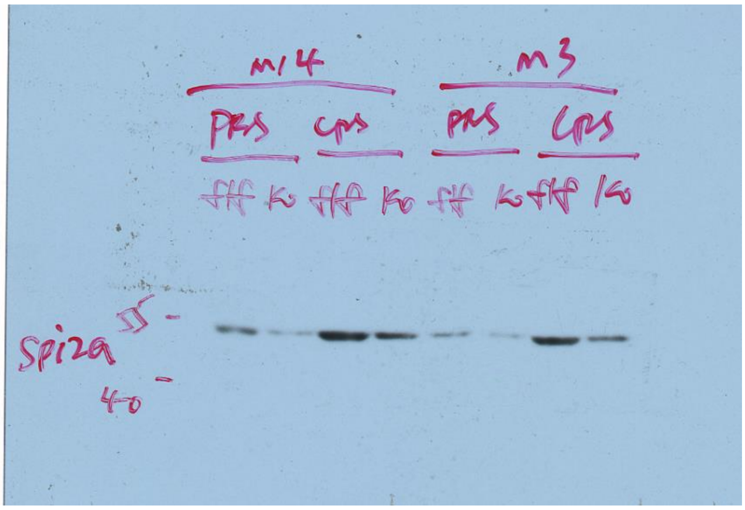

Supplementary figure 3d

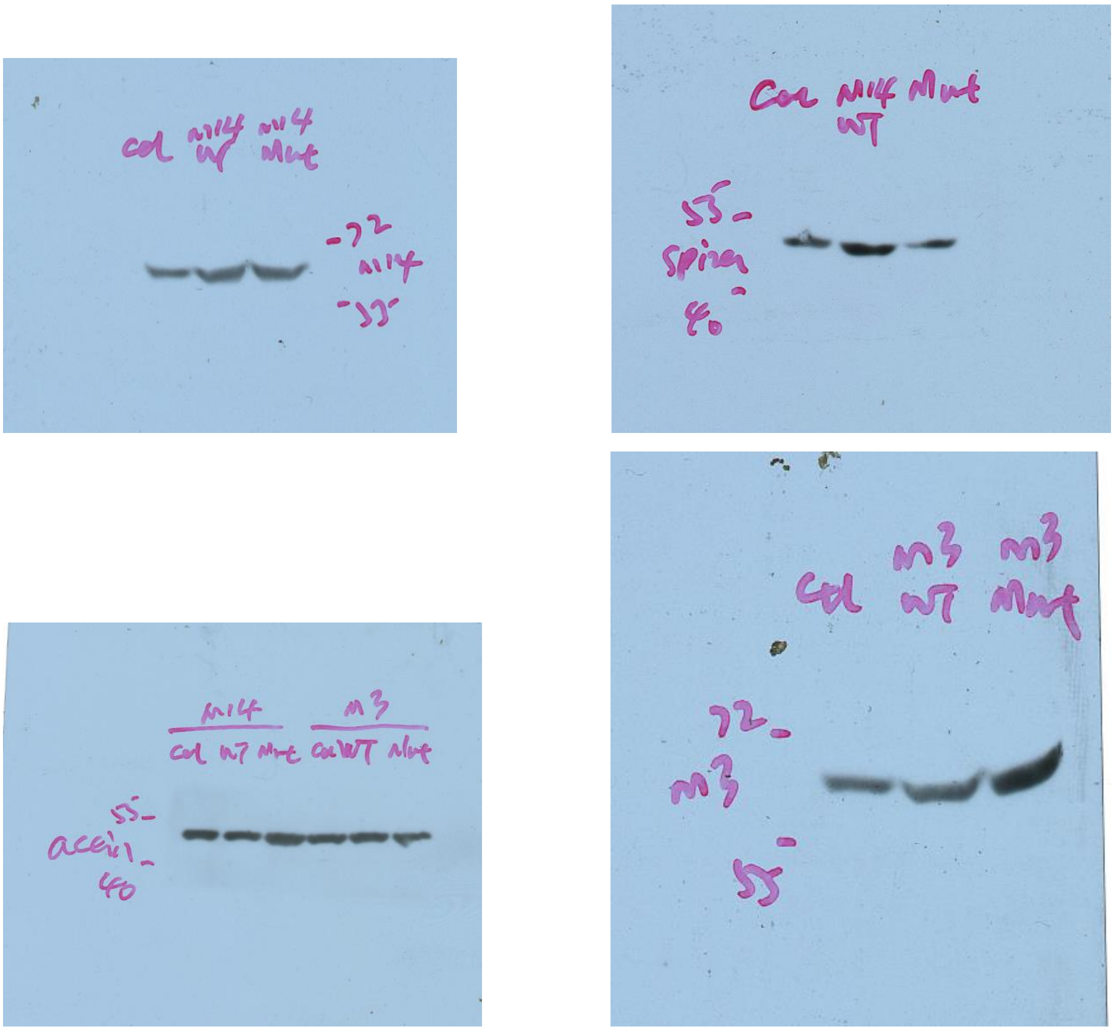

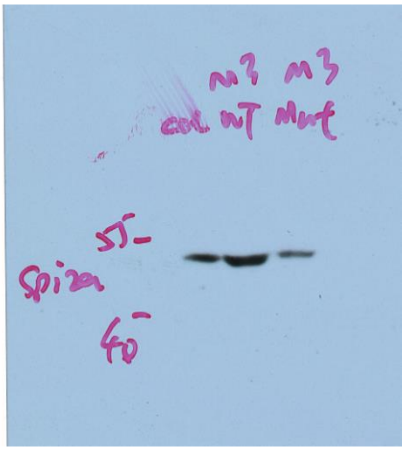

Supplementary figure 3i

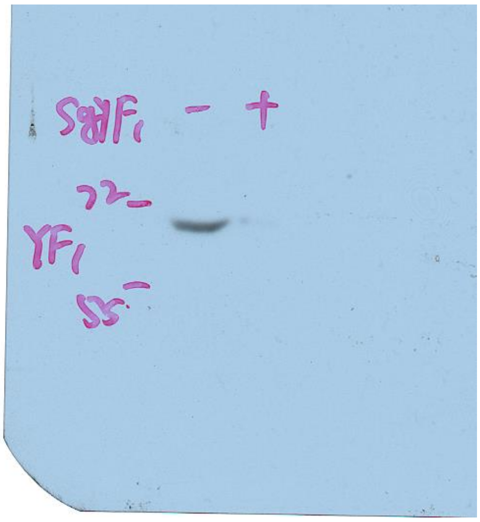

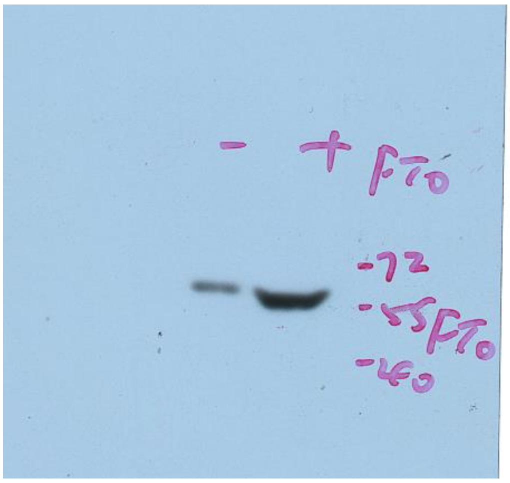

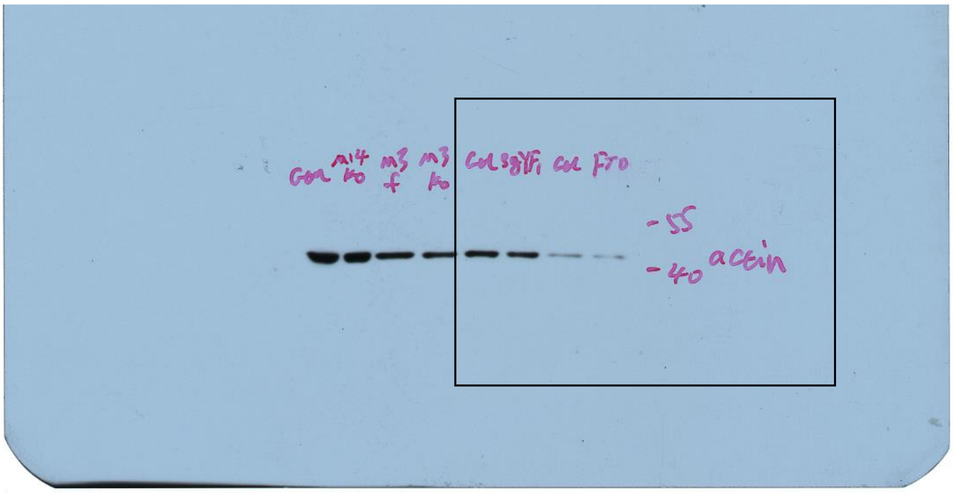

Supplementary figure 3j

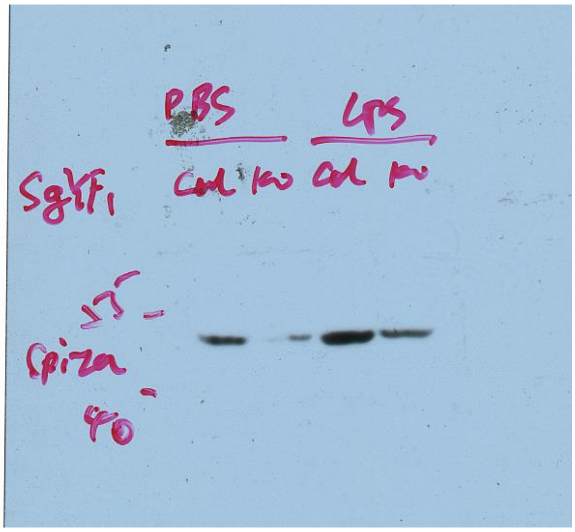

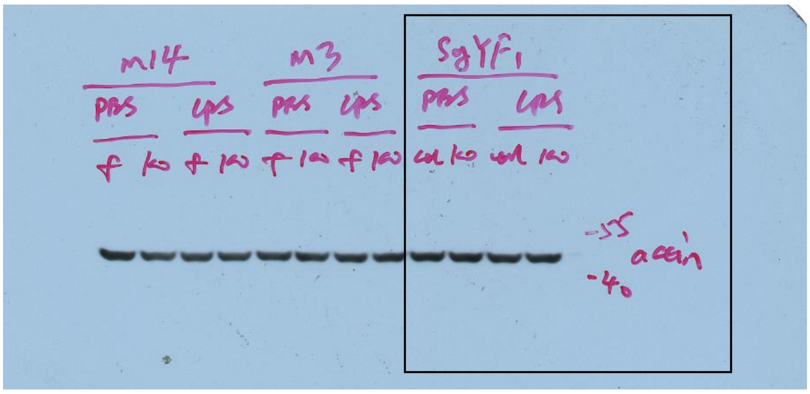

Supplementary figure 3l

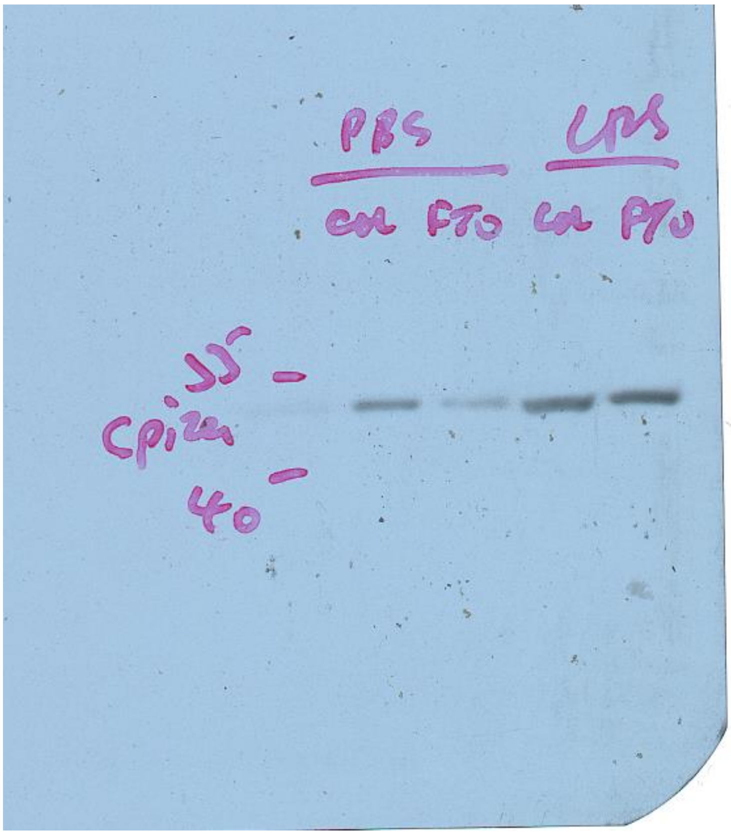

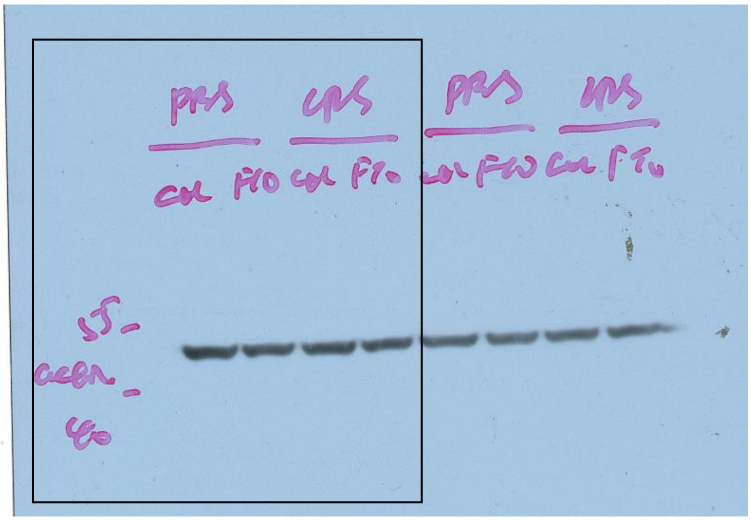

Supplementary figure 4a

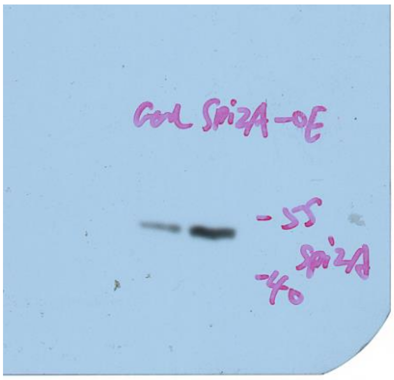

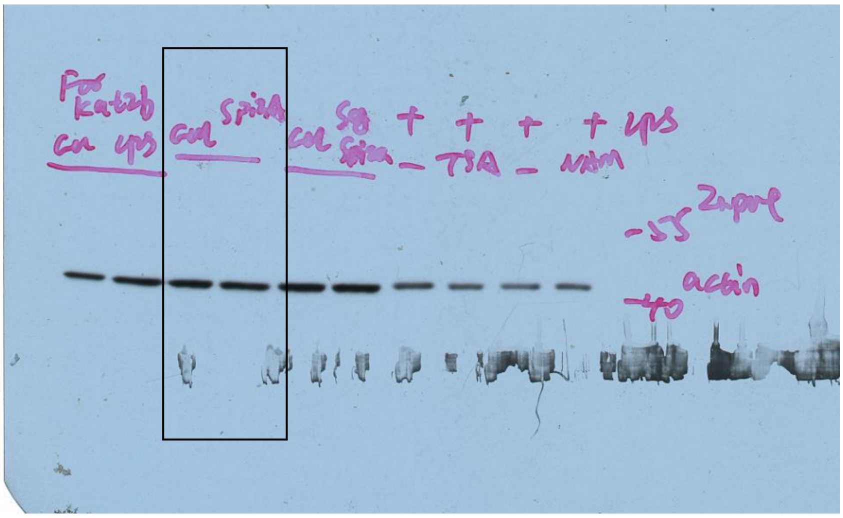

Supplementary figure 4d

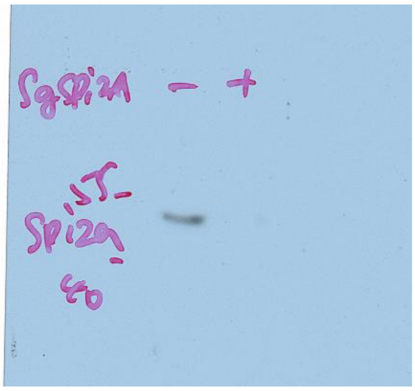

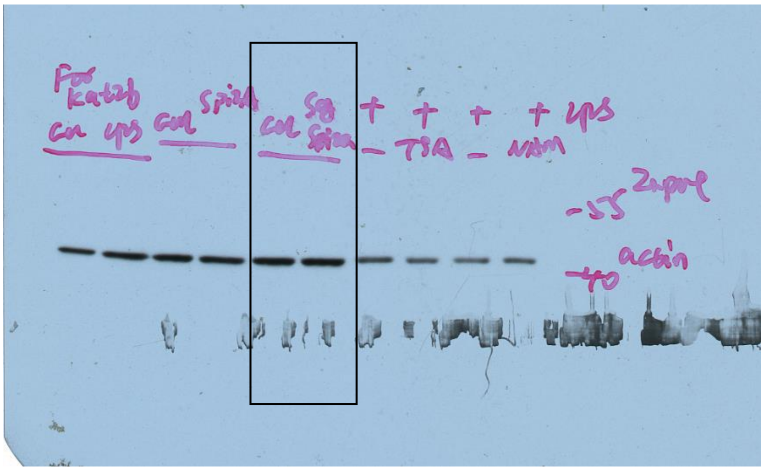

Supplementary figure 4h

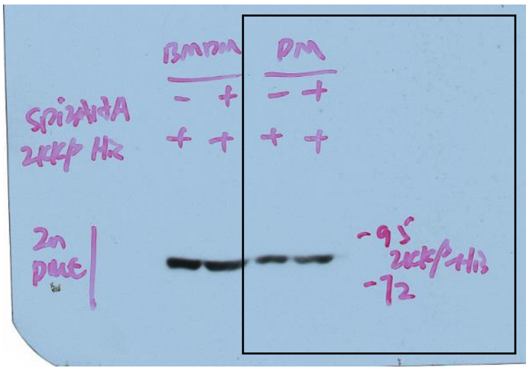
BMDM
PM
- + - +
+ + + +
-95
-72

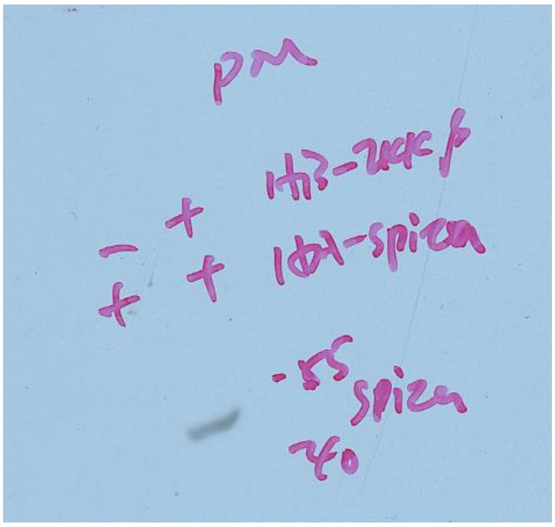
PM
- +
+ +
-55
40

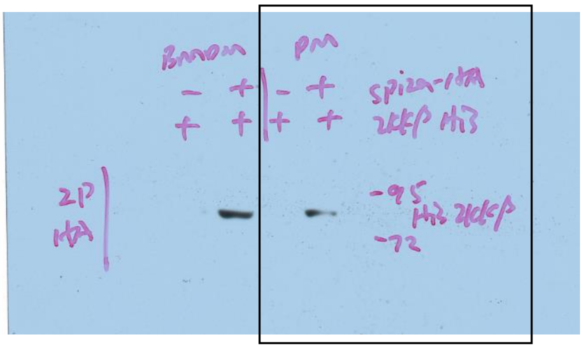
BMDM
PM
- + - +
+ + + +
-95
-72

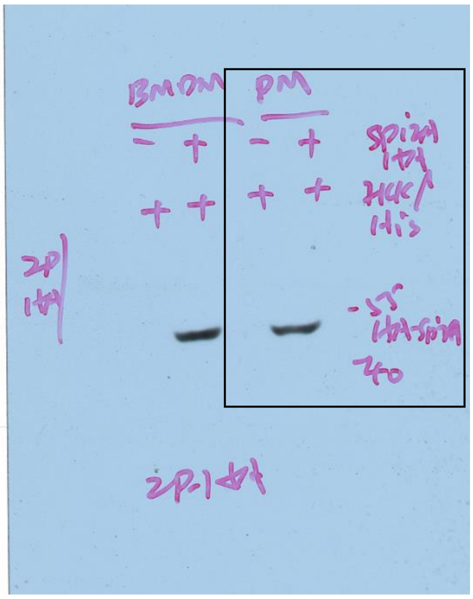
BMDM
PM
- + - +
+ + + +
-55
40

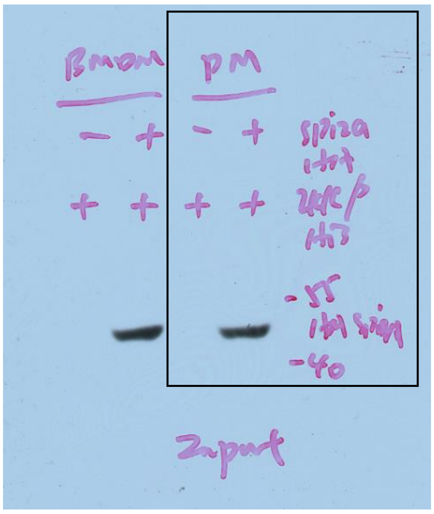
BMDM
PM
- + - +
+ + + +
-55
-40

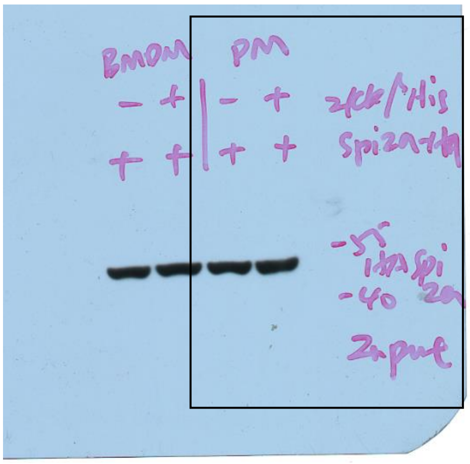
BMDM
PM
- + - +
+ + + +
-55
-40

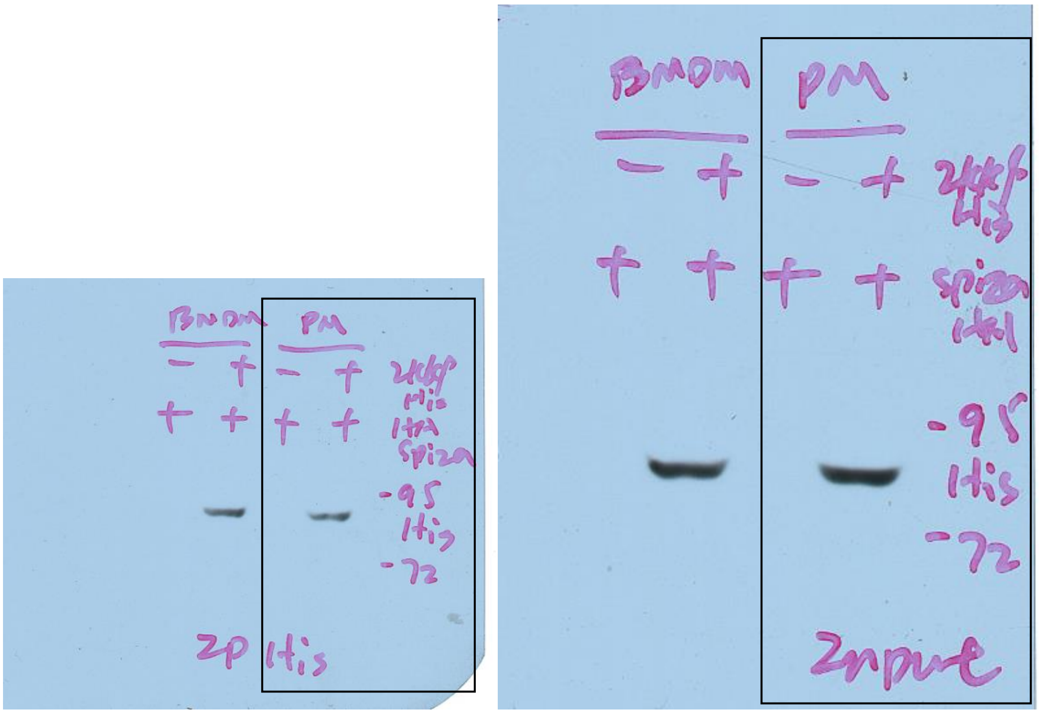

Supplementary figure 10c

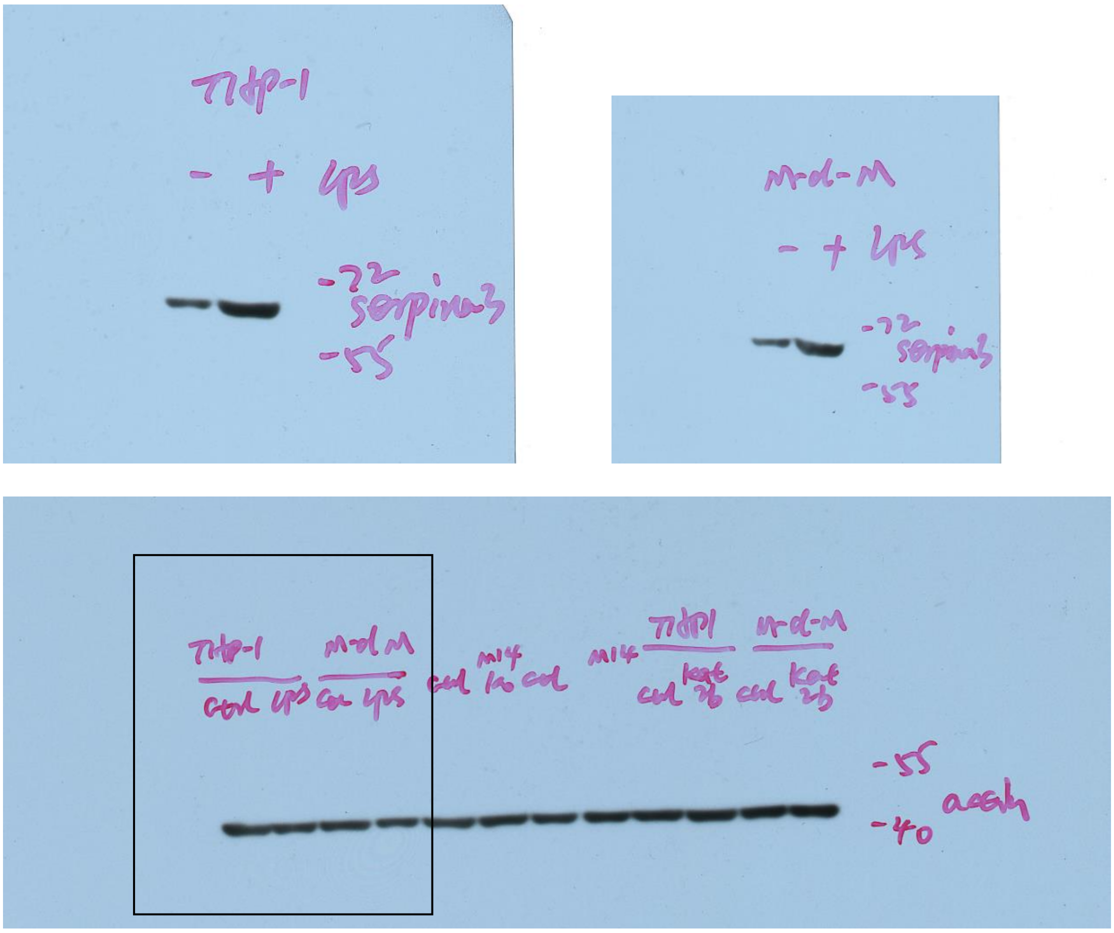

Supplementary figure 10d

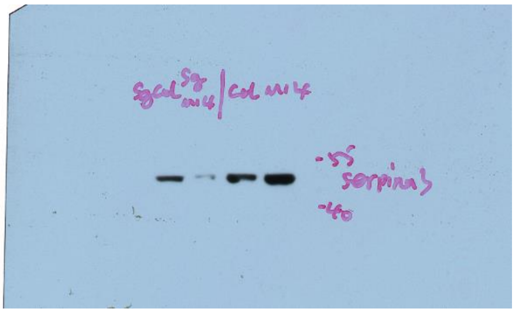

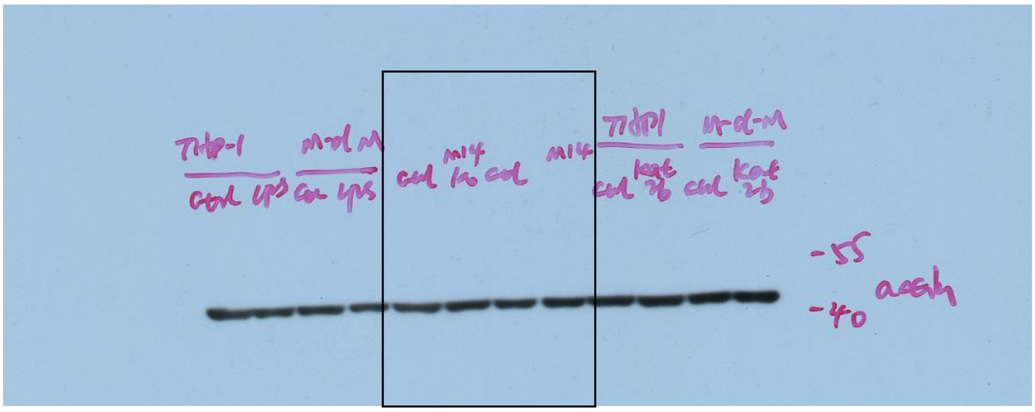

Supplementary figure 10i

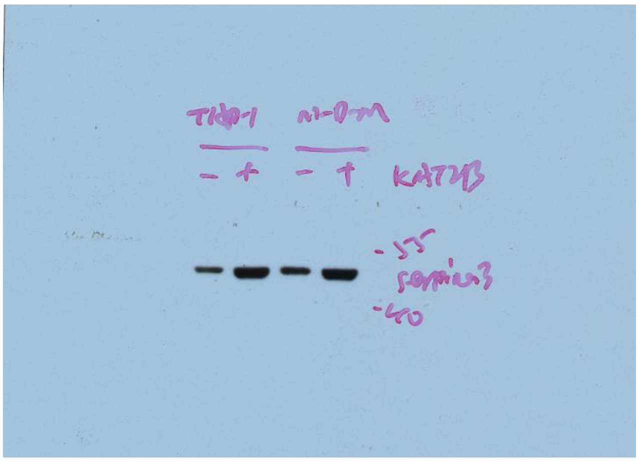

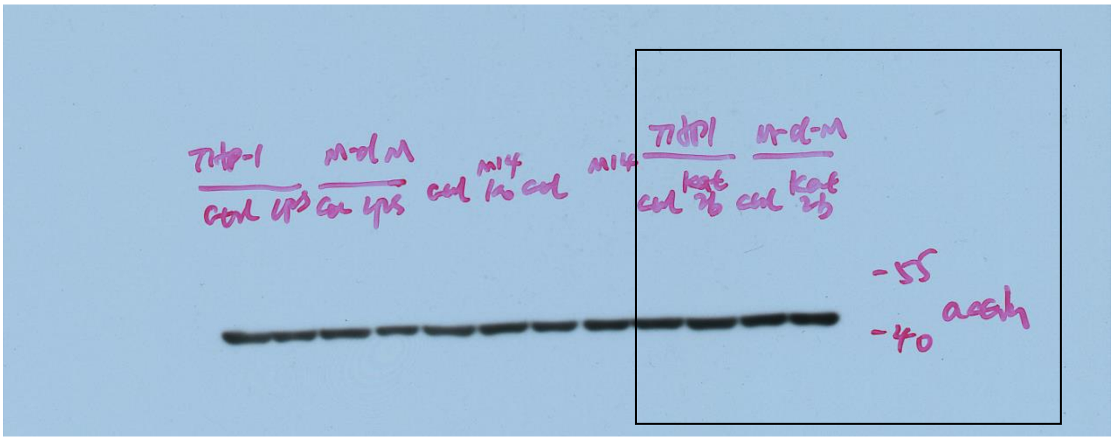

Supplementary figure 10j

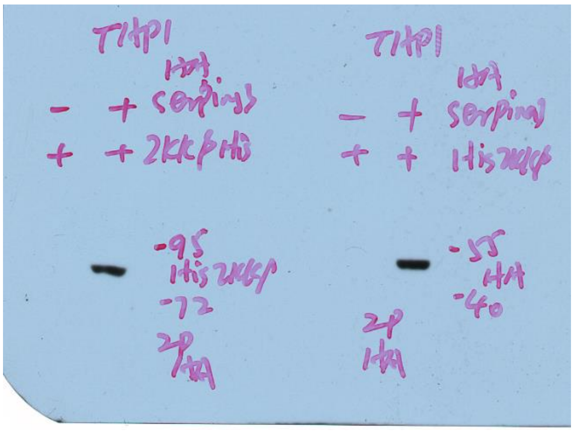
THP1
HA serpina3
- +
+ + His
-95
-72
IP HA
THP1
HA serpina3
- +
+ +
-55
HA
-40
IP HA

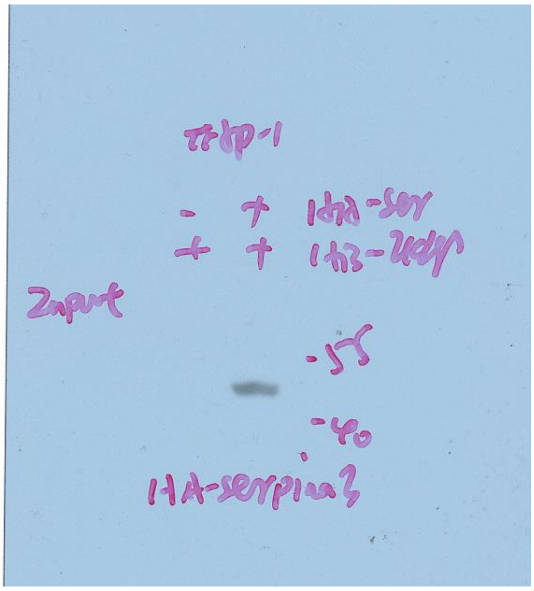
THP-1
- + HA-ser
+ +
Input
-55
-40
HA-serpina3

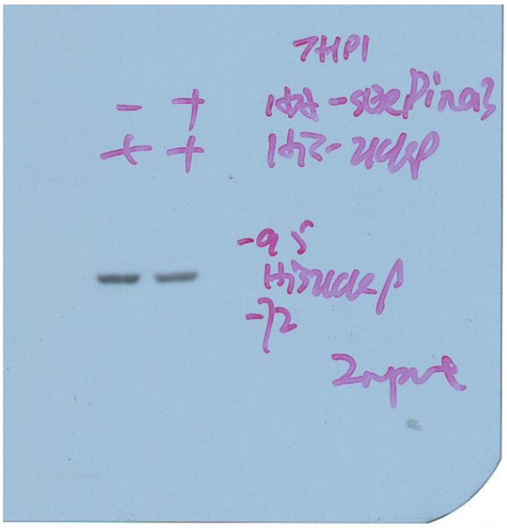
THP1
- + HA-serpina3
+ +
-95
-72
Input

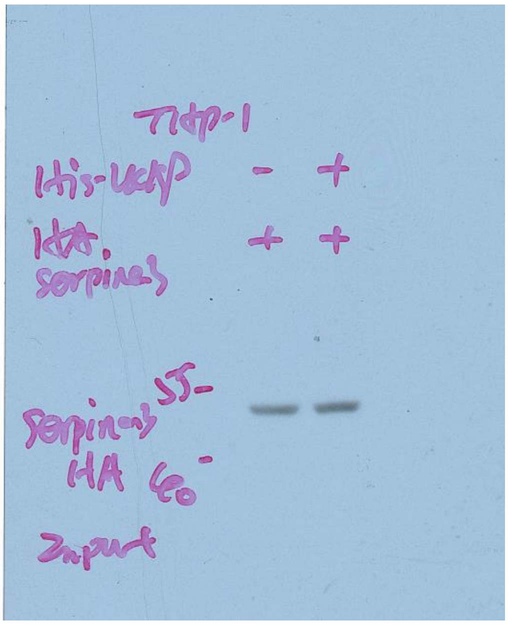
THP-1
- +
+ +
HA serpina3
55-
serpina3
HA 40-
Input

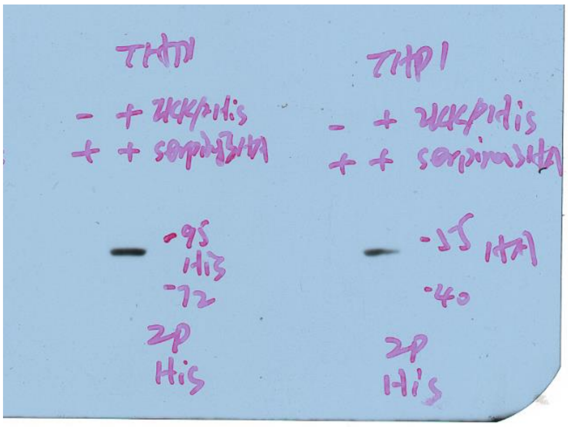
THP1
- +
+ +
-95
His
-72
IP His
THP1
- +
+ +
-55 HA
-40
IP His

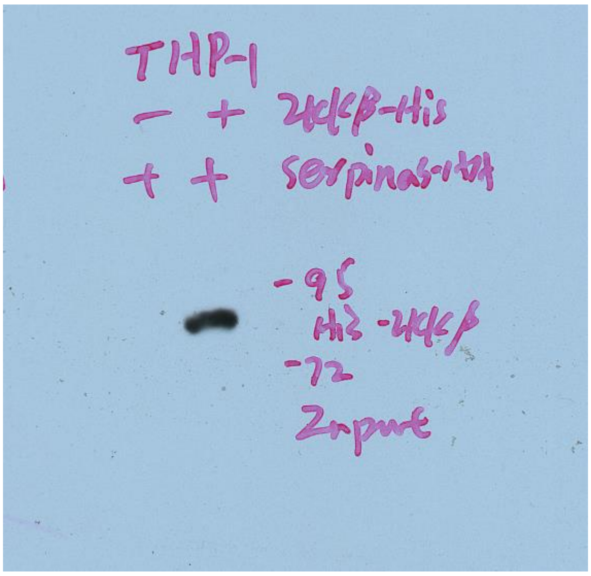

THP-1
- +
+ +
-95
-72
